# Degradation of Toxins and Metabolites of Cyanobacteria and Micropollutants during Biological Sand Filtration

**DOI:** 10.1021/acs.est.5c16532

**Published:** 2026-03-16

**Authors:** Valentin Rougé, Anne Dax, Oliver Köster, Urs von Gunten, Elisabeth M.-L. Janssen

**Affiliations:** † 28499 Eawag, Swiss Federal Institute of Aquatic Science and Technology, 8600 Dübendorf, Switzerland; ‡ Zurich Water Supply, 8021 Zurich, Switzerland; § School of Architecture, Civil and Environmental Engineering (ENAC), École Polytechnique Fédérale de Lausanne (EPFL), 1015 Lausanne, Switzerland

**Keywords:** cyanotoxin, drinking water treatment, enzymes, kinetic, biodegradation, biotransformation products

## Abstract

Cyanobacteria produce complex mixtures of secondary metabolites (cyano-metabolites), some of which are toxic and pose a growing concern for water utilities. While physical treatments such as filtration can efficiently remove cells, their lysis can release dissolved cyano-metabolites. This study investigated the efficiency of laboratory-scale sand filtration to abate 19 cyano-metabolites representing various structural classes. Furthermore, abatement of cyano-metabolites in a full-scale sand filtration is presented. Most cyano-metabolites showed abatement similar to or higher than the biodegradable benchmark micropollutants atenolol, paracetamol, and valsartan. Among cyano-metabolites, anabaenopeptins and cyanopeptolins had the highest abatement, while cyclamides and microcystin-LR had the lowest abatement. Abiotic controls and formation trends of 10 identified biotransformation products demonstrated that biodegradation played a major role in their removal. Laboratory-scale sand filters showed a sharp increase in biodegradation efficiency within days due to their adaptation to cyano-metabolites. Increasing the contact time and temperature both enhanced the abatement of most compounds, which could be kinetically modeled. High cyano-metabolite concentrations suppressed their own relative abatement, possibly due to metabolic enzyme inhibition or saturation. These findings suggest that sand filtration can serve as a dual-barrier against cyano-metabolites, including particle removal and biodegradation. However, biodegradation will be affected by the temperature and cyano-metabolite intake dynamics.

## Graphical abstract

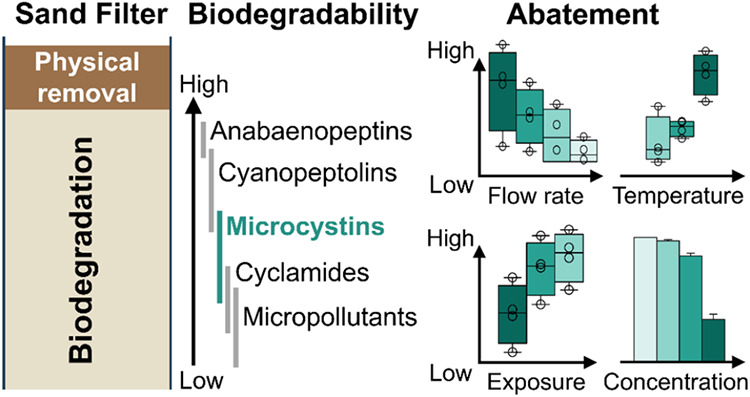

## Introduction

Cyanobacteria are among the most ubiquitous organisms on the globe, comprising almost 2000 identified species living in freshwater, terrestrial, or marine environments.^1,2^ Some cyanobacteria species can form dense blooms that can significantly degrade water quality, primarily by increasing turbidity and depleting oxygen through biomass decomposition after blooms subside. Additionally, they can release a complex mixture of metabolites (i.e., cyano-metabolites), some of which are recognized as toxins.^3^ These blooms have been increasing in intensity and frequency worldwide in the last decades and regularly impact water resources used for drinking water production.^3,4^ Cyano-metabolites can therefore enter water treatment plants^5^ and may even end up in the finished drinking water if no appropriate treatment is in place, requiring temporary safety warnings to the population.^6,7^ Recognizing the human health concerns, the World Health Organization (WHO) proposes chronic, lifetime, and acute short-term drinking water guideline values for four cyanobacterial toxins: microcystin-LR (MC-LR), cylindrospermopsin, anatoxin-a, and saxitoxin.^8^ Therefore, water suppliers need to account for the presence of cyano-metabolites in raw water, anticipate the probable intensification of blooming events, and develop mitigation strategies at the source and/or appropriate treatment.

Cyano-metabolites exhibit considerable chemical diversity, with 3084 compounds identified to date.^9,10^ While microcystins are among the most extensively studied, this class alone comprises 310 known congeners. Other classes such as anabaenopeptins and cyanopeptolins are also commonly detected, often at concentrations equal to or exceeding those of microcystins.^5,11–13^ While only four compounds are recognized as toxins by the WHO, other cyano-metabolites are reported to exhibit various bioactivities including inhibition of metabolic enzymes. For example, anabaenopeptins can inhibit phosphatases and carboxypeptidases,^14,15^ while cyanopeptolins can inhibit serine proteases.^12^

The removal of cyano-metabolites from drinking water by physical and chemical processes has mainly been studied for a few microcystin congeners.^16–19^ These studies led to the general consensus that physical treatment as a first step is the most effective measure since cyano-metabolites primarily enter the plant within cyanobacterial cells. Other practical considerations may, however, require oxidation as a first step, such as prevention of biofilm, algae, or mussel growth in the raw water intake pipes.^20^ Despite the fact that oxidation, notably ozonation, is efficient in degrading a vast range of cyano-metabolites such as microcystins, cylindrospermopsin, or anabaenopeptins,^21–23^ it can lead to counter-productive effects, i.e., the leaching of cyano-metabolites from damaged cells to the water phase without their subsequent degradation.^18^ In addition, ozonation can produce oxygen-rich byproducts such as aldehydes and carbonyls from reactions with the dissolved organic matter and phytoplankton, some of which can be toxic and can promote bacterial regrowth, especially in systems without disinfectant residuals in distribution systems.^24–26^ These drawbacks prompted a systematic use of a subsequent biofiltration step, such as rapid sand filtration after ozonation, which can also be potentially efficient to remove organic micropollutants and some of their transformation products.^27–30^ So far, (biological) sand filtration led to mixed results for the removal of microcystins from the water phase, ranging from no removal,^31,32^ to complete removal after acclimation periods ranging from a few to 200 days, depending notably on the history of the applied sand.^33–35^ Notably, Ho et al. observed a complete removal of microcystin-LA (MC-LA) and MC-LR within 4 min, suggesting a rapid biodegradation. Furthermore, the degradation in rapid sand filters has been studied for only a few microcystin congeners and saxitoxin. Other biodegradation studies focused on individual bacterial strains,^36–38^ while a few investigated water bodies, biofilms, and batch sand systems,^39–41^ but all largely focused only on selected microcystins. A recent study covered a broader range of cyano-metabolites, with their degradation in surface waters and enriched biofilm suspensions ranging from half-lives of a few hours to weeks, depending on the concentration of the biofilm and the type and concentration of cyano-metabolites.^42^ Due to the limited available literature data, it is not possible to infer about the efficiency of (biological) sand filtration for the removal of a wider range of cyano-metabolites during water treatment.

In this study, the biodegradation of 19 cyano-metabolites was assessed during (biological) sand filtration. The selected cyano-metabolites represent various structural classes including microcystins, anabaenopeptins, cyanopeptolins, and cyclamides. The effect of the chemical structure and concentrations of cyano-metabolites, as well as the contact time and temperature (4–21 °C) on abatement kinetics, was investigated in preozonated lake water. Identification of biotransformation products (bioTPs) and abiotic control experiments were performed to further confirm biodegradation. To ensure the relevance and transferability of the findings, a set of micropollutants with known biodegradabilities was included as benchmark compounds. An additional goal of adding some micropollutants was to generate novel biodegradation data that was limited or nonexistent, notably for sand filters. Finally, results from laboratory-scale experiments were compared with a sampling campaign in a full-scale drinking water treatment plant.

## Material and Methods

### Chemicals and Reagents

The purity and suppliers of all chemicals and solvents are provided in Table S1 (Supporting Information 1, SI1). Aerucyclamide A was previously isolated from *Microcystis aeruginosa* PCC7806, purified, and kept in DMSO.^43^ Stock solutions of anabaenopeptin A and anabaenopeptin B were standardized by their molar absorption coefficients ε_278_ = 4190 and 2300 M^–1^ cm^–1^, respectively, in methanol.^44^ Stock solutions of the micropollutants were prepared in ultrapure water (Arium Pro, Sartorius, 18.7 MΩ cm). Ozone (O_3_) stock solutions were prepared by sparging O_3_/O_2_ gas mixtures produced by an O_3_ generator (BMT 803 BT, BMT Messtechnik, Berlin, Germany) in ultrapure water cooled in an ice bath. The concentrations of the O_3_ stock solution (0.9–1.3 mM) were measured spectrophotometrically at 260 nm in a 1 cm quartz cuvette (ε_260_ = 3200 M^–1^ cm^–1^).^45^

### Cyanobacterial Cultures and Extraction

*Microcystis aeruginosa* PCC7806 and *Planktothrix rubescens* K-0576 were obtained from the Pasteur Culture Collection (PCC) and the Norwegian Culture Collection of Algae (NORCCA), respectively. Cyanobacteria were cultivated, and their biomass was extracted and purified by solid-phase extraction as previously described.^22,46^ A ^15^N *P. rubescens* batch was also cultivated to be used as an internal standard (Text S1.1, SI1).

### Drinking Water Treatment Plant Sampling

The drinking water treatment plant (DWTP) of Zürich in Lengg (Switzerland) was sampled in January 2024, following the cyano-metabolites along the treatment train (see Scheme S1, SI1). Due to the mixing of Lake Zürich during the winter and a depth of the intake pipe at 30 m,^47^ cyanobacteria typically reach the plant in January (see the seasonal cyano-metabolite intake over the period of 2010–2024 in Figure S1, SI1). Raw water (pH = 8.4 ± 0.2, [DOC] = 1.5 ± 0.1 mgC L^–1^) and sand used for the laboratory-scale experiments were also sampled at DWTP Lengg in February 2024 and January 2025. Details on the sampling are provided in Text S1.2 (SI1).

### Sand Column Experiments

Laboratory-scale sand filtration experiments were conducted with an Omnifit glass column (2.5 cm inner diameter, 5 cm height, Diba Industries). Two experimental sequences were performed, 6 months apart, hereafter referred to as “column #1” and “column #2.” Sand sampled from the DWTP was packed in the glass column between 10 μm-pore PTFE frits after purging the trapped air. The resulting column pore volumes and dispersions were measured using a NaCl tracer (see details in Text S2, SI1). The column was wrapped in aluminum foil to protect from light and conditioned for 2 weeks by pumping lake water, freshly ozonated with 0.5 mgO_3_ L^–1^ (0.3 gO_3_g DOC^–1^, no residual O_3_ remaining), with a M480 HPLC pump (Gynkotek, Germany) at 0.25 (column #1) or 0.5 mL min^–1^ (column #2). After the conditioning period, experiments were conducted with ozonated lake water spiked with two cyanobacterial extracts and 13 micropollutants: acesulfame, atenolol, carbamazepine, diclofenac, gabapentin, lamotrigine, molinate, paracetamol, sucralose, tramadol, triclosan, valsartan, and valsartan acid (see details and selection criteria in Text S1.4, SI1). The list of all micropollutants, identified cyano-metabolites, and their concentrations are shown in Tables S2 and S3, and compound structures are shown in Schemes 1, S2 and S3 (SI1). Experiments were conducted at various flow rates (0.25–4.0 mL min^–1^), temperatures (4–21 °C), and spiked concentrations (between 0.1–0.6 and 0.6–6.3 μg L^–1^ for micropollutants and 1.9–19 mg_biomass_ L^–1^ for cyano-metabolites, see individual compound concentrations in Tables S2 and S3). In column #2, an abiotic control experiment was also conducted after extracting, autoclaving (two times, 121 °C and 2 bar for 15 min), and refilling the sand column. It is important to note that in the case of column #1, pretests were also conducted at high cyano-metabolite concentrations (192 mg_biomass_ L^–1^, i.e., ten times higher than the highest concentration spiked in column #2) and 0.6–6.3 μg L^–1^ of micropollutants between the conditioning and the final experiments. Further details on the experiments are provided in Text S1.4, and the conditions and timeline of the different experiments are provided in Tables S4 and S5 (SI1). For simplicity, each experimental condition was labeled by a letter code A-M, followed by a number if conducted multiple times in the same column (e.g., C1, C2···). All relative abatement data are provided in Tables S6 and S7 (SI1).

**Scheme 1:**
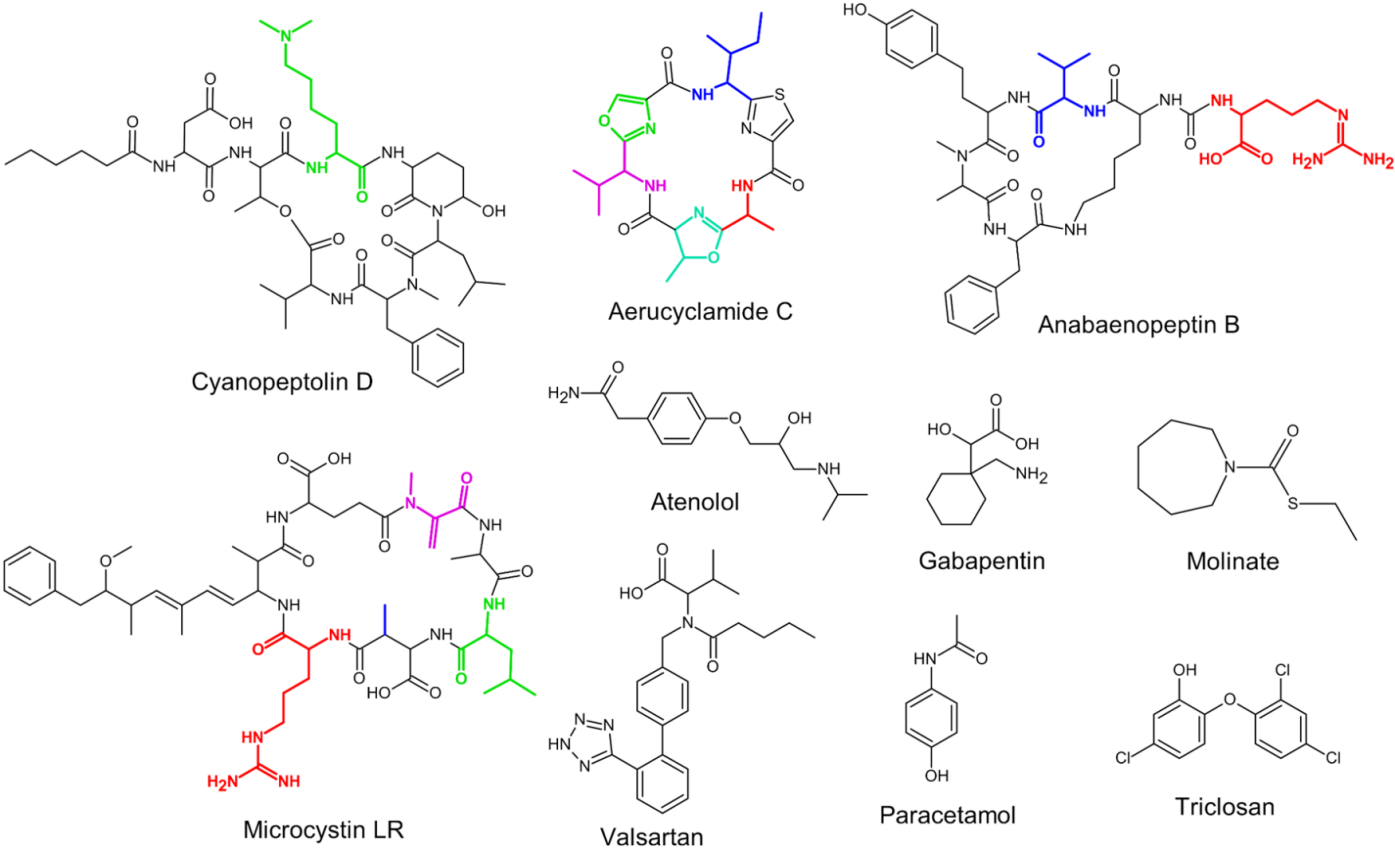
Structures of Selected Cyano-Metabolites and Micropollutants^a^

a Highlighted moieties represent the parts that can vary in other cyano-metabolites of the same class identified in this study. All of the other cyano-metabolite and micropollutant structures are provided in Schemes S2 and S3 (SI1).

### Analyses of Cyano-Metabolites, Micropollutants, and BioTPs

Cyano-metabolites from the water treatment plant sampling were analyzed by an online solid-phase extraction HPLC-HRMS/MS method described elsewhere.^11,48^ Cyano-metabolites, micropollutants, and their bioTPs from sand column experiments were analyzed by HPLC (Dionex Ultimate 3000 RS pump, Thermo Fischer Scientific) with an Atlantis T3 C18 column (3 μm, 3.0 × 150 mm, with the corresponding VanGuard precolumn, Waters), coupled to a high-resolution tandem mass spectrometer (HRMS/MS, Exploris, ThermoFisher Scientific). Details for the HPLC and HRMS/MS methods are provided in Text S3 (SI1). The monitored cyano-metabolites were previously identified by MS^2^ annotation.^22^ Identified bioTPs are given in Table S11 (SI1), and their MS^2^ annotations are given in a separate spreadsheet (SI2).

### Statistical Analyses

The abatement of micropollutants and cyano-metabolites in laboratory sand columns, as well as the effect of temperature, was assessed by kinetic modeling. Details of the kinetic modeling are provided in Text S4.1 (SI1), including the determination of apparent first-order rate constants k and activation energies Ea. The bias introduced by the ideal plug-flow approximation used for the kinetic assessment was evaluated and is discussed in Text S4.2 (SI1). The statistical comparison between zero- and first-order kinetic fits is presented in Text S4.3 (SI1). In addition, the compound-specific differences between given experiments were assessed using two-sample Welch’s *t* tests with α = 0.05 (results shown in Table S7).^49^ Finally, similarities in compound abatement behavior were assessed using hierarchical clustering based on Euclidean distances and the average-linkage (UPGMA) algorithm, a classical method originally introduced in numerical taxonomy.^50^ Details and explanations of this method are provided in Text S4.4 (SI1).

## Results and Discussion

### Fate of Cyano-Metabolites in the Selected Drinking Water Treatment Plant

The cyano-metabolites were monitored along the treatment train of the DWTP Lengg, Switzerland. The four main cyano-metabolites entering the DWTP with the raw water were the microcystin [d-Asp^3^,(E)-Dhb^7^]­MC-RR and the three anabaenopeptins, anabaenopeptin A, anabaenopeptin B, and oscillamide Y. They mostly entered the DWTP associated with the cyanobacterial biomass, with a particulate-phase concentration of 2.00 μg L^–1^ (2.38 nM) for the total anabaenopeptins and 0.44 μg L^–1^ (0.43 nM) for [d-Asp^3^,(E)-Dhb^7^]­MC-RR (filled bars, Figure 1). By comparison, only 0.12 μg L^–1^ (0.14 nM) of anabaenopeptins and 0.05 μg L^–1^ (0.05 nM) of [d-Asp^3^,(E)-Dhb^7^]­MC-RR were present in the water phase (patterned bars, Figure 1). After the preozonation step, the aqueous concentration increased by 1.15 and 0.22 μg L^–1^ for anabaenopeptins and [d-Asp^3^,(E)-Dhb^7^]­MC-RR, respectively. This increase in aqueous concentration was equivalent to the decrease in particulate-phase concentration, i.e., by 1.00 μg L^–1^ for anabaenopeptins and by 0.21 μg L^–1^ for [d-Asp^3^,(E)-Dhb^7^]­MC-RR. Overall, preozonation could not abate cyano-metabolites but rather transferred them from particulate to aqueous phase. Consequently, a mixture of dissolved and particulate cyano-metabolites reached the top of the sand filter (Figure 1). Within the first 40 cm of the sand filter, i.e., in the pumice stone layer, most of the particulate cyano-metabolites were removed (from 1.26 to 0.19 μg L^–1^), while the total aqueous concentrations only slightly decreased from 1.53 to 1.25 μg L^–1^. Beyond 40 cm, i.e., in the quartz sand layer, the aqueous concentration of cyano-metabolites gradually decreased to 0.05 μg L^–1^, yielding >90% cyano-metabolites abatement across the whole sand filter.

The decrease in particulate cyano-metabolite concentrations in the top layer of the sand filter is expected due to physical removal of the particles. The subsequent abatement of dissolved cyano-metabolites in the deeper layers of the sand filter may be due to biodegradation, which is further investigated in this study. A previous pilot study in the same DWTP revealed biological activity of a pilot-scale sand filter, demonstrating partial abatement of the biodegradable micropollutants atenolol, paracetamol, and valsartan.^28^ Valsartan was also present in the raw water, reaching the top of the rapid sand filter during the 2024 sampling, and although not quantified, its peak area decreased by about 60% through the filter (crosses in Figure 1), consistent with the partial abatement previously observed in the pilot plant.^28^ With ≥ 90% abatement, the removal of the cyano-metabolites was more efficient than that of valsartan. In addition, four known bioTPs of anabaenopeptin A, anabaenopeptin B, and oscillamide Y (ana-TP679, ana-TP636, ana-TP693, and ana-TP650, see Table S11 for structures) were formed within the sand filter (circles and triangles in Figure 1), further supporting an abatement of cyano-metabolite via biotransformation. In the following, we investigated parameters affecting biotransformation in laboratory sand column experiments.

### Sand Column Experiments

#### Effect of Flow Rate

The abatement of cyano-metabolites at different water flow rates in the sand column filled with sand from the DWTP is presented in Figure 2a as box plots, categorized by their class including anabaenopeptin, microcystin, cyanopeptolin, and cyclamide, as well as planktocyclin as a single compound. The abatements of biodegradable micropollutants are shown in Figure 2b as a bar chart. Other spiked micropollutants (acesulfame, carbamazepine, diclofenac, lamotrigine, sucralose, tramadol, and valsartan acid) did not exhibit measurable abatement and are not shown. At a flow rate of 1 mL min^–1^, >90% of anabaenopeptins, 75% of planktocyclin, 30–78% of cyanopeptolins, <10% to 57% of microcystins, and <15% of cyclamides were removed in column #2. The limited abatement observed for MC-LR in column #2 (<10%) is consistent with the limited abatement previously observed for MC-LR within the first 2 days of exposure of a sand filter to microcystins.^34^ Only a handful of micropollutants had measurable removal (Figure 2b). At 1 mL min^–1^, 78% of triclosan was removed, while atenolol, paracetamol, and valsartan abatement ranged between 14 and 35%. Gradually increasing the flow rate decreased the abatement of all compounds in both columns (Figure 2). This effect is expected since a higher flow rate reduces the residence time of the compounds in the column and, in the case of biodegradation, the contact time between compounds and biofilm and/or enzymes. It is worth mentioning that a higher flow rate could, in theory, lead to similar observations in the case of adsorption, if the used bed volumes are nearing the breakthrough. In-depth discussion on evidence for biodegradation versus adsorption is provided in the section “Further experimental evidence of biodegradation.”

**Figure 1:**
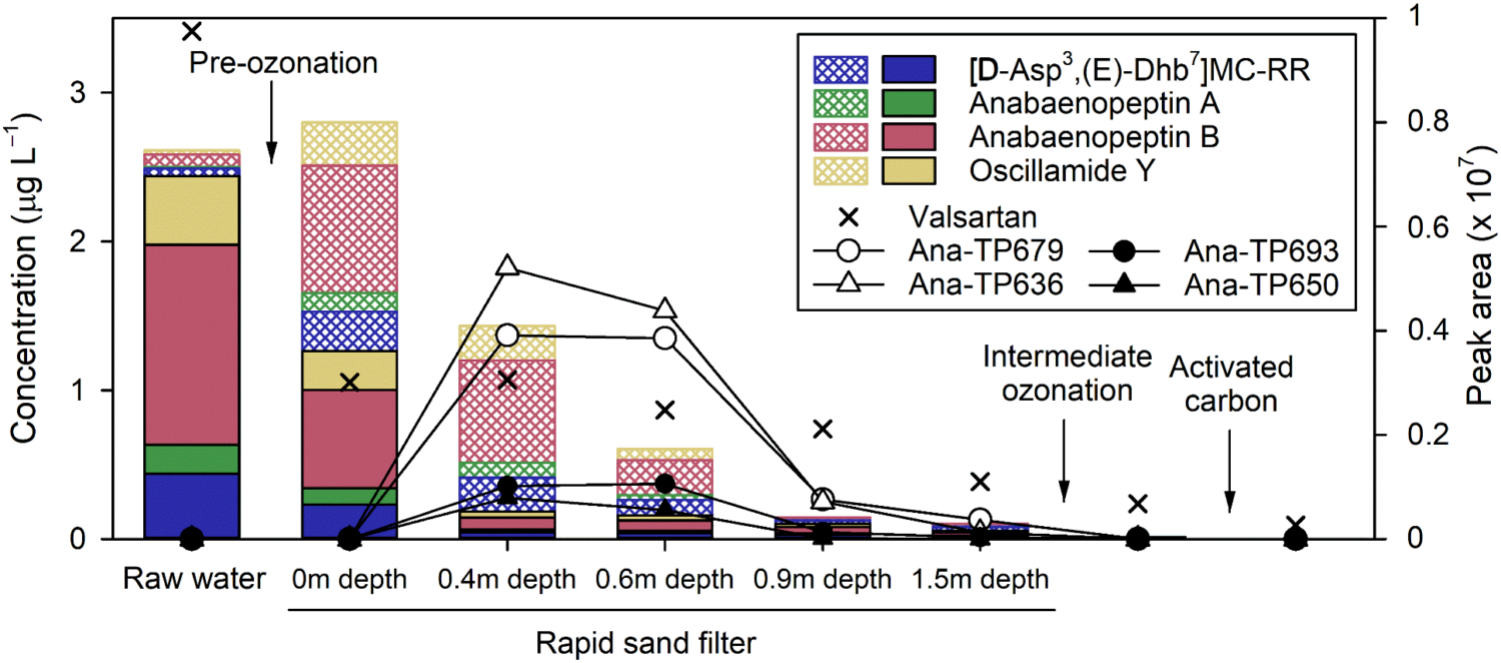
Concentrations in μg L^–1^ of anabaenopeptin A, anabaenopeptin B, oscillamide Y, and [d-Asp^3^,(E)-Dhb^7^]­MC-RR across the treatment train of the drinking water treatment plant Lengg (Zürich, Switzerland). Filled bars represent the particulate-phase concentrations, while patterned bars represent the aqueous-phase concentrations. Aqueous abundances of valsartan (crosses) and of four anabaenopeptin bioTPs ana-TP679, ana-TP636, ana-TP693, and ana-TP650 (circles and triangles) are shown as peak areas. Conditions: [DOC_raw water_] = 1.5 mgC L^–1^, temperature = 7 °C, [preozonation] = 0.5 mgO_3_ L^–1^, sand vertical velocity = 1.2 m h^–1^, and [intermediate ozonation] = 0.3 mgO_3_ L^–1^.

**Figure 2:**
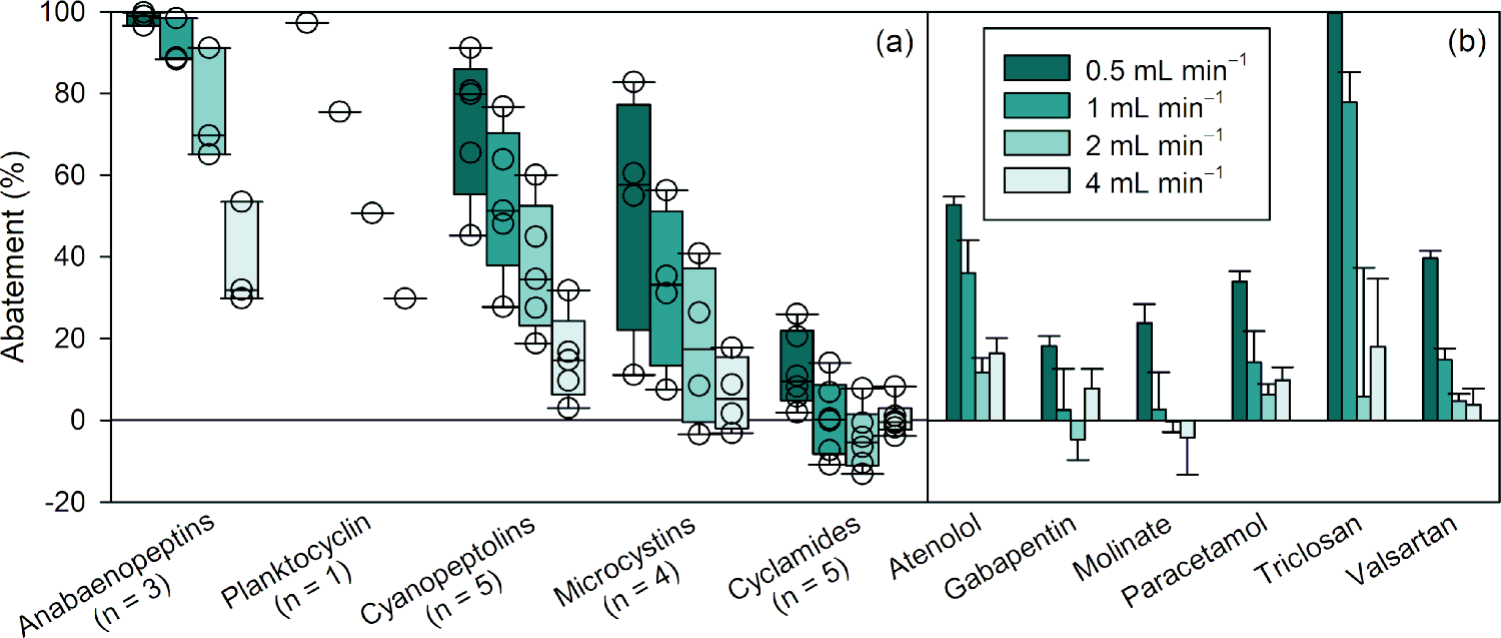
Effect of flow rate on the relative abatement of (a) cyano-metabolites and (b) micropollutants in laboratory sand column #2 (experiments labeled B, C1, D, and E1, see Table S4, SI1). Cyano-metabolites are grouped by class in box plots, with n representing the number of individual compounds in each class. All data are provided in Table S7 (SI1). Experimental conditions: temperature = 21 °C, [cyano-metabolites] = 19.2 mg_biomass‑equivalent_ L^–1^, [micropollutants] = 0.6–6.3 μg L^–1^.

The same conditions in column #1 tested 6 months prior to column #2 led to significantly higher removal for cyano-metabolites, while no clear trend was observed for micropollutants (Figure S10 for column #1 results and Table S7 for statistical tests, SI1). The origin of the higher abatement observed in column #1, due to adaptation upon pre-exposure to cyano-metabolites, is discussed in the section “Effect of column exposition to cyano-metabolites and micropollutants”.

#### Temperature Effect

Water treatment processes are rarely conducted at the standard temperatures used for laboratory experiments. The sampled DWTP operates at a water temperature of around 7 °C. When decreasing the temperature of the water and column, the abatement of cyano-metabolites and micropollutants also decreased (Figure 3). From 21 to 11 °C, the abatement decreased by 45–49% for anabaenopeptins, 35–62% for cyanopeptolins, 29% for MC-LR, 43–55% for the other microcystins, 19–28% for cyclamides, 19% for planktocyclin, and 18–27% for atenolol and valsartan. Decreasing the temperature to 4 °C further decreased the abatement of cyano-metabolites and micropollutants. Similar temperature effects were observed in column #1 (Figure S11, SI1). Such an effect is expected as enzymatic/chemical reactions are sensitive to temperature, notably due to its effect on molecular kinetic energy, which affects the frequency and intensity of collisions between enzymes and substrates.^51^ Only triclosan abatement was not affected or even slightly increased with decreasing temperature.

**Figure 3:**
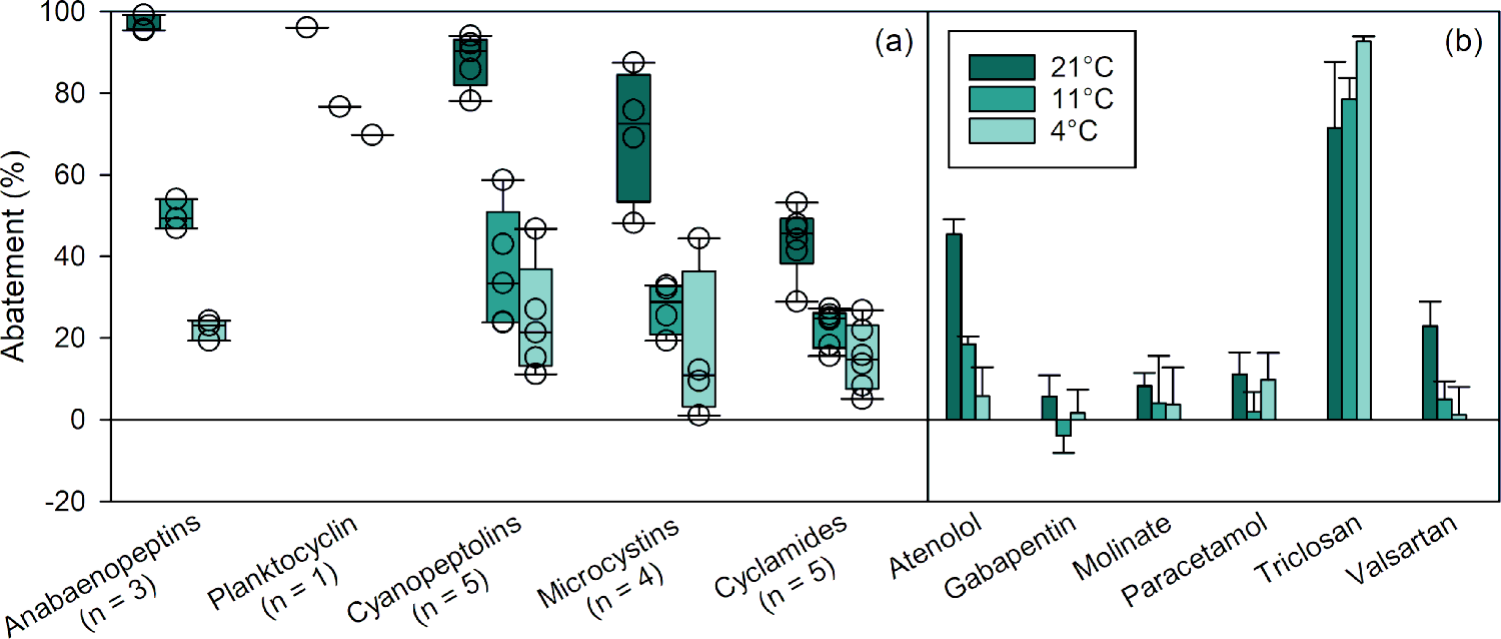
Effect of temperature on the relative abatement of (a) cyano-metabolites and (b) micropollutants in laboratory sand column #2 (experiments labeled C3, F, and G, see Table S4, SI1). Cyano-metabolites are grouped by class in box plots, with n representing the number of individual compounds in each class. All data are provided in Table S7 (SI1). Experimental conditions: flow rate = 1 mL min^–1^, [cyano-metabolites] = 19.2 mg_biomass‑equivalent_ L^–1^, and [micropollutants] = 0.6–6.3 μg L^–1^.

#### Effect of Column Exposition to Cyano-Metabolites and Micropollutants

As discussed in the section “Effect of flow rate,” the cyano-metabolite abatement in column #1 was consistently higher than in column #2. This was attributed to the pre-exposure of column #1 to cyano-metabolites. In column #1, pre-experiments were conducted for 7 days at high cyano-metabolite concentrations (192 mg_biomass‑equivalent_ L^–1^) and with 0.6–6.3 μg L^–1^ micropollutants (same concentrations as for column #1) before acquiring the data presented in this study. In contrast, experiments with column #2 were conducted without pre-exposure (see experimental timeline in Table S5, SI1). To assess the adaptation, the same experiment (flow rate: 1 mL min^–1^, 21 °C) was repeated three times in column #2 after 1, 4, and 25 days of exposure to cyano-metabolites and micropollutants. The corresponding results are shown in Figure 4, along with the analogous experiment conducted in column #1.

Between 1 and 4 days of operation, the abatement increased by 11–28% for cyclamides, 17–60% for cyanopeptolins, 27–32% for microcystins, and 20% for planktocyclin (Figure 4a). Anabaenopeptin removal was already at >90% after 1 day of operation. The increase in abatement between 1 and 4 days was statistically significant (α = 0.05) for all of the cyano-metabolites except planktocyclin and microcyclamide 7806A (see Table S7, SI1). Further increase in abatement between 4 and 25 days was only significant for microcystins and cyclamides, likely due to a maximized abatement already reached after 4 days for anabaenopeptins and cyanopeptolins. Conversely, no clear trend was observed for micropollutants (Figure 4b). The increase in cyano-metabolites abatement with operation time was confirmed in other experiments at a higher flow rate (Figure S12, SI1), especially for anabaenopeptins, for which removal was not maximized.

The progressive increase in cyano-metabolite abatement upon exposure points to an adaptation of the columns. A similar improvement was generally observed within a cyano-metabolite class, while the concentration of the cyano-metabolites within one class largely varied (Table S3, SI1). For instance, [d-Asp^3^,(E)-Dhb^7^]­MC-RR concentration was 287 nM (294 μg L^–1^), while the other microcystins had individual concentrations of 3–12 nM (3–12 μg L^–1^) (Table S3, SI1). Despite these concentration discrepancies, the abatement of all microcystins increased to a similar extent between 1 and 4 days of exposure (27–32%) (Figure 4). Similar observations were made for the other cyano-metabolite classes, i.e., similar abatement increase despite different individual concentrations (Figure 4 and S12, Table S3, and SI1). Although the presence of specific degradation genes could not be verified, enzymes such as microcystinase A are known to enable the catabolic cleavage of several microcystins.^52,53^ The identification of microcystin bioTPs expected from microcystinase A suggests that analogous mechanisms occur in the column (see section “Identification of biotransformation products”). Similarly, other cyano-metabolites may share common enzymes, e.g., peptidases potentially produced by various organisms in the sand column rather than specialized degraders, that can contribute to their degradation, at least within the same cyano-metabolite class. Their additive concentrations, including potentially unidentified cyano-metabolites, may have contributed to triggering an increase in enzyme expression. Conversely, the known biodegradable moieties in the selected micropollutants largely differ, i.e., primary amide for atenolol,^54^ aromatic cycle for diclofenac,^55^ primary amine/carboxylic acid for gabapentin,^56^ thiocarbamate for molinate,^57^ secondary aromatic amide for paracetamol,^58^ anisole/tertiary amine for tramadol,^59^ and secondary amide for valsartan.^54^ Micropollutants, therefore, likely do not share many common enzymes, and their individual concentrations may have been below thresholds needed to induce the enzymes responsible for their degradation.^60^ The mechanisms and specific enzymes behind the column adaptation to cyano-metabolites remain to be investigated. The abundance of genes of enzymes suspected to be responsible for the degradation of MC-LR has previously been shown to increase upon exposure to MC-LR.^61^ Future studies combining column experiments with the bacterial community and functional gene dynamic analysis could therefore provide insights into the adaptation to cyano-metabolites, although interpretation will remain challenging for compounds beyond microcystins.

**Figure 4:**
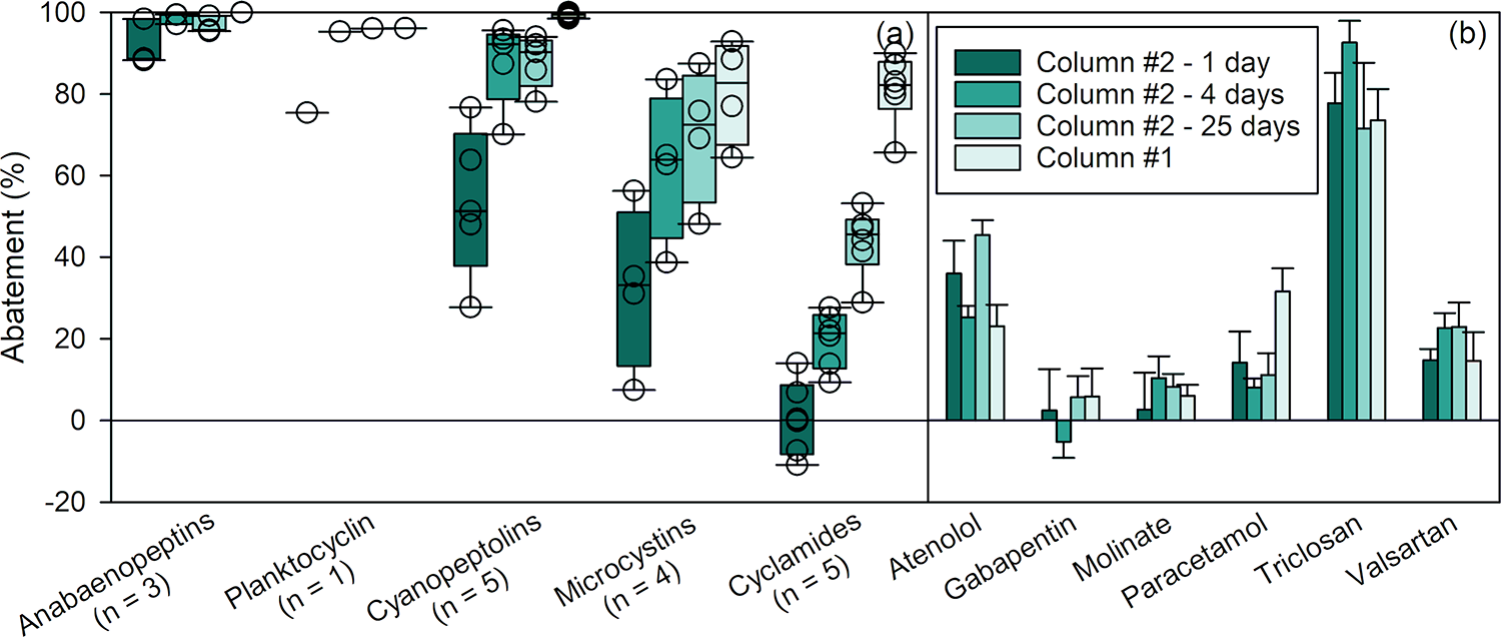
Relative abatements of (a) cyano-metabolites and (b) micropollutants in column #2 and after different operation times. The same experiment was repeated 1, 4, and 25 days after the start of the column #2 experiments (experiments labeled C1, C2, and C3, see Table S4, SI1). The same experiment was conducted once in column #1 after a week of pre-exposure to cyano-metabolites micropollutants (experiment labeled C). Cyano-metabolites are grouped by class in box plots, with n representing the number of individual compounds in each class. All data are provided in Table S7 (SI1). Experimental conditions: flow rate = 1 mL min^–1^, temperature = 21 °C, [cyano-metabolites] = 19.2 mg_biomass‑equivalent_ L^–1^, and [micropollutants] = 0.6–6.3 μg L^–1^.

#### Effect of Cyano-Metabolite Concentration

The effect of cyano-metabolite and micropollutant concentrations on their degradation is shown in Figure 5 for selected compounds. While repeated or continuous exposure to cyano-metabolites enhanced their removal, higher concentrations of cyano-metabolites inhibited their removal. This was evident when gradually diluting the spiked cyano-metabolites and micropollutants (Figure 5). Because of the adaptation of the column, undiluted replicates that were run before (replicate 1) and after (replicate 2) diluted samples are shown. Upon a 2-fold dilution of cyano-metabolites and micropollutants, the abatement of cyanopeptolin D, aerucyclamide A, microcystins, and planktocyclin increased significantly compared to both undiluted replicates (α = 0.05, see statistical test results in Table S7, SI1). Due to the adaptation, the increase observed compared to the undiluted replicates 1 and 2 is likely overestimated and underestimated, respectively.

This concentration-dependent abatement is consistent with a previous study conducted in batch reactors using biofilms grown in surface water.^42^ By comparison, micropollutant abatement was not significantly enhanced by the 2-fold dilution (α = 0.05, Table S7, SI1). Additionally, varying the concentration of micropollutants while keeping cyano-metabolite concentrations at the same level led to <10% difference in abatement for all compounds, except for molinate and paracetamol (Figure S13, SI1).

Increasing relative abatement upon dilution of substrates (cyano-metabolite or micropollutant) suggests two likely scenarios: saturation of enzymes and/or inhibition. Enzyme saturation occurs at high substrate concentrations when all of the enzymatic sites are occupied, making the substrate conversion rate independent of its concentration. In this case, a 2-fold dilution would therefore lead to a doubled relative abatement. Upon 2-fold dilution, the relative abatement of cyanopeptolin D, [d-Asp^3^,(E)-Dhb^7^]­MC-RR, and MC-LR increased by factors of 1.1–3.3, 1.3–2.4, and 1.4–7.1, respectively, depending on the undiluted replicate used. The expected increase factor, therefore, falls in between the observed ranges. Further investigation of enzyme saturation via kinetic fitting is discussed in the next section.

Enzyme inhibition by a broad spectrum of compounds is well-documented and can be another cause for decreased relative abatement at higher cyano-metabolite concentrations. The limited influence of substrate concentration on the abatement of micropollutants suggests that the general microbial activity was not significantly inhibited. Instead, specific extracellular enzymes involved in cyano-metabolite degradation may have been affected. Several cyano-metabolite classes are known to inhibit certain enzymes such as carboxypeptidases, phosphatases, or serine proteases.^14,62,63^ Similar inhibition might occur for cyano-metabolite-degrading enzymes such as peptidases.^42,53^

Whether the observed effects result from enzyme saturation, inhibition, or a combination of both remains to be elucidated for individual compounds. Nevertheless, it is evident that increasing cyano-metabolite concentrations can reduce the efficiency of their abatement, without significantly affecting micropollutants degradation.

### Kinetic Considerations

The abatement of cyano-metabolites and micropollutants was kinetically modeled to gain insights into their degradation within the sand filter and to derive quantitative parameters, i.e., apparent first-order abatement rate constants (*k*_app_) and activation energies (*E*_a_), for comparison with the literature. It is important to note that the kinetic fitting and determined empirical parameters may be influenced by a combination of processes. They include multiple enzyme kinetics, mass transfer, sorption/desorption, and other physicochemical interactions, which cannot be disentangled with our experimental setup.^64^ The kinetic order and *k*_app_ are discussed hereafter, and the determination of apparent activation energies is discussed in SI1 (Text S4.5).

#### Kinetic Order

Considering that the abatement observed in the sand column is driven by enzymatic reactions, first-order kinetics may be applied as long as the substrate concentration is low enough relatively to the enzymes.^51,65^ If the substrate concentration is too high, enzymes may become saturated, and kinetics become zero-order.^51^ Using the abatement observed at various residence times (Figures 2 and S10, SI1), apparent first- or zero-order rate constants *k*_app_ can be calculated (see details in Text S4.1, SI1). The bias introduced by the ideal plug-flow approximation was simulated and was expected to be minor (minimal loss of linearity and <50% underestimation of *k*, Text S4.2, SI1).^65^ For the first-order, reasonable correlations were obtained for most compounds (*R*_2_ > 0.8 for 20 and 15 compounds in columns #1 and #2, respectively, Table S8, SI1) as long as sufficient data points were available (10% < abatement <99.9%). In comparison, zero-order gave worse fits, suggesting that complete saturation of enzymes did not occur (details are provided in Text S4.3, SI1). The concentration effect shown in Figure 5 may, therefore, result principally from inhibition rather than saturation. However, partial saturation cannot be dismissed (substrate concentration near the Michaelis constant *K*_m_), leading to a mixed kinetic order, which cannot be further elucidated by our experimental data.

**Figure 5:**
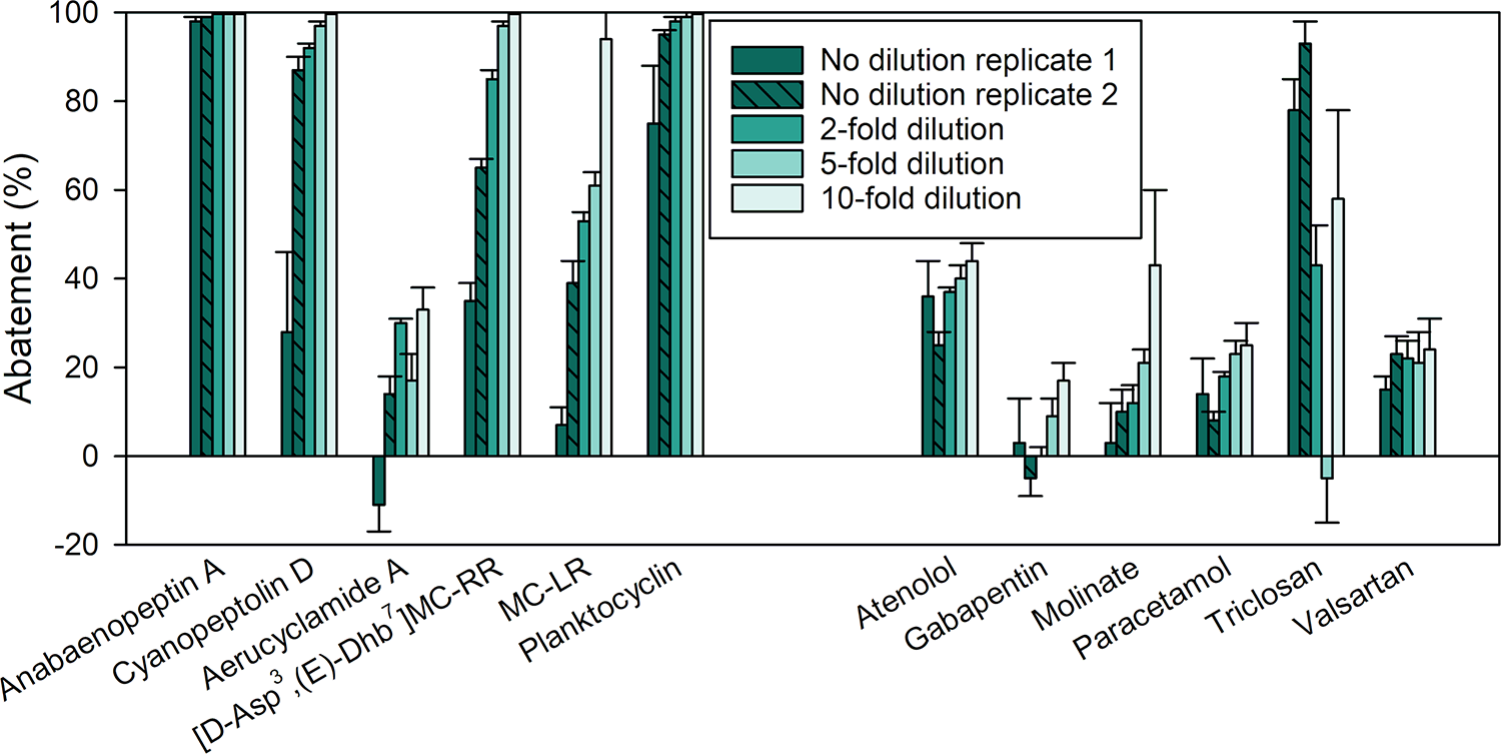
Relative abatement of selected cyano-metabolites and micropollutants at different cyano-metabolite and micropollutant concentrations (experiments labeled C1, C2, I, J1, and K, see Table S4, SI1). “No dilution” refers to the default concentration, e.g., cyano-metabolites from 19.2 mg_biomass‑equivalent_ L^–1^ extract and 0.6–6.3 μg L^–1^ of micropollutants. The mixture was then diluted up to 10-fold in ozonated lake water. All data are provided in Table S7 (SI1). Experimental conditions: flow rate = 1 mL min^–1^ and temperature = 21 °C.

#### Determination of Apparent First-Order Abatement Rate Constant

In column #1, the apparent first-order abatement rate constants (*k*_app_) were ∼2 × 10^–2^ s^–1^ for anabaenopeptins, ∼9 × 10^–3^ s^–1^ for cyanopeptolins, and ∼(5–30) × 10^–4^ s^–1^ for cyclamides and microcystins (Table S8, SI1). These *k*_app_ were between 3- and 15-fold lower for column #2 due to a lack of pre-exposure to cyano-metabolites (see section “Effect of column exposition to cyano-metabolites and micropollutants”). *k*_app_ determined in column #2 may therefore reflect a more intrinsic baseline capacity to degrade cyano-metabolites. By comparison, micropollutants had similar *k*_app_ values in both columns, with less than a 3-fold difference. Atenolol, paracetamol, gabapentin, molinate, and valsartan had *k*_app_ ranging between 1 and 6 × 10^–4^ s^–1^ (Table S8, SI1).

The *k*_app_ of paracetamol and molinate was about 1 order of magnitude lower compared to a previous biological sand filtration study (2–4 × 10^–3^ s^–1^).^27,66^ The faster removal of paracetamol and molinate observed in the previous study can partly be attributed to the longer operation time of the filter (one year) and the finer sand used (0.45 mm compared to 0.7–1.2 mm used in our study). These experimental differences likely promoted biomass growth, acclimation to the target compounds, as well as contact between compounds and microbial enzymes. So far, kinetics for cyano-metabolite abatement in sand filters have not been measured. However, a recent study investigated the biodegradation of cyano-metabolites by river-grown biofilms.^42^ Expectedly, *k*_app_ values were between 1 and 3 orders of magnitude higher for the sand filter than for river-grown biofilm batch kinetics (at equivalent cyano-metabolite concentration). This is likely due to a higher biomass density and surface area in sand filters. Despite the differences in *k*_app_, the relative degradation of the different cyano-metabolites was consistent between sand filters and river-grown biofilms: anabaenopeptins and cyanopeptolins degrade faster than microcystins and cyclamides (Figure 2, Table S8, SI1).^42^ Moreover, the faster abatement of microcystins and anabaenopeptins compared to valsartan in the column experiment is consistent with the observations from the full-scale drinking water treatment plant (Figure 1).

### Abatement Trends across Compound Classes

Across experiments, consistent patterns for biodegradability appeared with anabaenopeptins being removed the fastest, followed by cyanopeptolins, microcystins, and cyclamides. By comparison, micropollutants, except triclosan, were removed at rates comparable to microcystins or cyclamides in the first days, but their abatement gradually became lower than that of all cyano-metabolites over time.

To evaluate whether the above trends were statistically robust, hierarchical clustering was performed and visualized as a dendrogram (Figure 6). Significant clusters (AU *p*-value ≥ 95%) with the greatest Euclidean distance are shown in different colors, representing the largest possible clusters. The first cluster (purple) consisted of all microcystins except MC-LR. The second cluster (red) contained all cyclamides together with MC-LR. The third cluster (green) comprised all micropollutants except triclosan (compounds showing no abatement were omitted from this analysis), and the fourth cluster (blue) grouped anabaenoptins, cyanopeptolins, and plantocyclin. Triclosan did not cluster with any compound.

**Figure 6:**
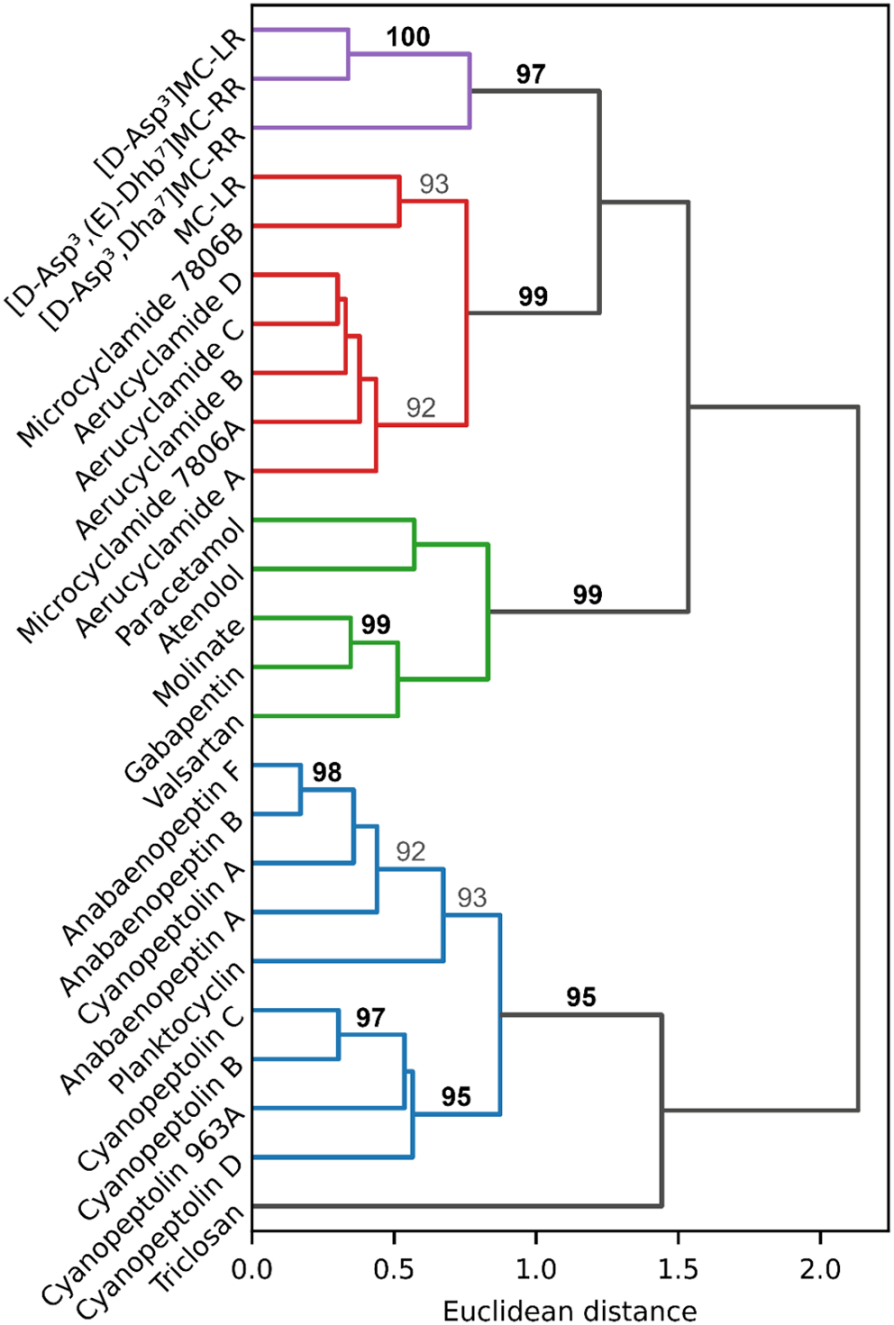
Dendrogram of the hierarchical clustering of cyano-metabolites and micropollutants using abatement data across all experiments. The strength of clusters is represented by the AU *p*-value. Only AU *p*-values representing strong (≥95%, shown in bold) and moderate (90–94%, shown in gray) significance are shown.

This clustering statistically confirmed the separation in abatement behavior between cyano-metabolites and micropollutants, as well as the general cyano-metabolite class-specific behavior discussed above, with some nuances. MC-LR clustered with cyclamides instead of microcystins. The robustness of this grouping was high (AU *p*-value = 99%), while the clustering of the microcystin cluster (purple) with the cyclamides/MC-LR cluster (red) was not significant (AU *p*-value <90%, not shown). This indicates that the structural similarities between MC-LR and the other microcystins did not translate into similar abatement within the studied set of cyano-metabolites.

The similar abatement of anabaenopeptins may be linked to their side chain attached by a ureido bond. This has previously been suggested to be a favorable biodegradation attack site,^42^ for which transformation products were detected in this study (see section “Identification of biotransformation products”). The similarly high abatement of cyanopeptolins is suggested to be due to the presence of an ester, which is a favorable site of attack for enzymatic reaction.^67^ Nevertheless, no transformation products supporting an ester hydrolysis have been found to date. For microcystins, the presence of arginine has previously been suggested to be important in enhancing their biodegradability.^42^ This is consistent with the well-known degradation pathway of several microcystins, in which the arginine neighboring the adda group is the primary enzymatic attack site.^68,69^ The significantly lower biodegradability of MC-LR compared to [d-Asp^3^,(E)-Dhb^7^]­MC-RR, [d-Asp^3^, Dha^7^]­MC-RR, and [d-Asp^3^]­MC-LR, all of which contain at least one arginine, remains, however, unexplained. The methylation of the aspartic acid in MC-LR, the only modification between MC-LR and [d-Asp^3^]­MC-LR, may play a role in the lower abatement of MC-LR. However, our data are insufficient to support this hypothesis. For cyclamides, little is known apart from a reported cyclic peptide bond hydrolysis,^37^ which, if occurring during the present column experiments, does not appear to be initially favorable.

Overall, this clustering clearly supports that cyano-metabolite abatement was influenced by structural similarities. Specific structural features are important in overcoming the general biodegradation resistance granted by the cyclic nature of these peptides.^70^ Cyano-metabolites within the same class share some of these key structural features, allowing the abatement of a given compound to be preliminarily inferred from measurements of other cyano-metabolites within that class.

### Further Experimental Evidence of Biodegradation

The target compound abatement observed in the sand column is hypothesized to be mostly due to biodegradation. However, processes such as adsorption could also be partially responsible for the abatement observed in Figures 2–5. In sand columns with little adsorption capacity, breakthrough is usually expected to occur fast. But our experiments used <90 bed volumes, potentially low enough to observe adsorption. To further evaluate the role of abiotic processes on the abatement of cyano-metabolites and micropollutants, the abatement was compared before and after autoclaving the sand. In addition, the formation of identified bioTPs was compared before and after autoclaving.

#### Cyano-Metabolite and Micropollutant Abatement in Control Experiments with Autoclaved Sand

Overall, autoclaving of the sand efficiently mitigated the abatement of all compounds, except for triclosan, aerucyclamide D, and planktocyclin (Figure 7 and S14, SI1). These results suggest that biodegradation responsible for the abatement of most compounds was efficiently neutralized by autoclaving. The efficient abatement of triclosan before and after autoclaving suggests that it is mostly driven by adsorption in both cases. This is supported by a decrease in abatement over time (circles in Figure 7a), characteristic of an adsorption breakthrough, and by the lack of a temperature effect on its removal (Figure 3b). For aerucyclamide D and planktocyclin, the removal is hypothesized to be mainly due to an abiotic oxygenation of the methionine moiety to sulfoxide (Text S5, SI1). For all other compounds, some abatement was observed in the first 2 h of operation time (first sampling point corresponding to 12 bed volumes), which then progressively decreased to ≤10% abatement after 5 h except for cyanopeptolins and atenolol, which were still abated by 19–50% (Figure 7b–e and S14, SI1). As discussed for triclosan, the decrease in abatement as a function of the experiment duration hints at a breakthrough of an adsorption process. This suggests that some initial adsorption occurred after autoclaving, which became negligible after 5 h for most compounds. It is important to note that no breakthrough-like behavior was observed across all experiments before autoclaving, except for triclosan (data not shown). Therefore, if adsorption occurred, it was likely not a major process.

The high abatement remaining after 5 h for cyanopeptolins and atenolol remains unclear (Figures 7b and S14, SI1). Despite being relatively polar, high sorption coefficients in biofilm (>1 L g^–1^) have been reported for atenolol,^71^ likely linked, in part, to its protonated state (p*K*_a_ of secondary amines is around 9–10). Previous studies further confirm the higher adsorption of atenolol compared to micropollutants such as carbamazepine or diclofenac.^72^ In comparison, cyanopeptolins are neutral at pH < 9 (except for cyanopeptolin 963A, which is negatively charged), and their sorption to biofilm is unknown. The extent of adsorption of cyanopeptolins and atenolol in experiments before autoclaving could not be quantified, but the absence of a breakthrough-like abatement trend suggests that biodegradation remains the main removal process. To further investigate the role of biodegradation in cyano-metabolite and micropollutant abatement, bioTPs were identified and monitored.

**Figure 7:**
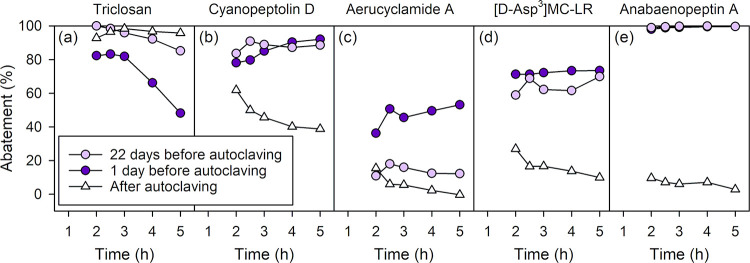
Effect of autoclaving on the relative abatement of triclosan and selected cyano-metabolites in column #2. The relative abatement is shown as a function of the experiment time for (a) triclosan, (b) cyanopeptolin D, (c) aerucyclamide A, (d) [d-Asp^3^]­MC-LR, and (e) anabaenopeptin A. The abatement is shown 1 day and 22 days before autoclaving the column (circles) and after autoclaving (triangles) (experiments labeled C3, C2, and M, see Table S4, SI1). Experimental conditions: flow rate = 1 mL min^–1^, temperature = 21 °C, [cyano-metabolites] = 19.2 mg_biomass‑equivalent_ L^–1^, and [micropollutants] = 0.6–6.3 μg L^–1^.

#### Identification of Biotransformation Products

To further demonstrate that biotransformation occurred and is responsible for the main cyano-metabolite and micropollutant abatement in the column before autoclaving, 7 known and two newly identified bioTPs were monitored (Text S6 for details, structures provided in Table S11, SI1, and MS^2^ annotations in SI2). Two anabaenopeptin bioTPs, ana-TP679 (dealkylation-like of the ureido bond) and ana-TP636 (hydrolysis at the ureido bond), as well as four microcystin bioTPs, MC-TP980 (arginase reaction product), MC-TP614, MC-TP543, and MC-TP460 (peptide hydrolysis) were previously reported.^41,42,61,68^ In addition, one microcystin bioTP, MC-TP885 (peptide hydrolysis), and one cyanopeptolin bioTP, cyanopeptolin-TP818, were newly identified. The known bioTPs of atenolol and gabapentin, atenolol acid (amide hydrolysis) and gabapentin-lactam (intramolecular cyclization and dehydration), respectively, were also detected.^56,73^

The formation of three bioTPs, namely, atenolol acid, ana-TP636, and MC-TP614 is shown in Figure 8 as a function of the relative abatement of their respective precursors for all column experiments. The formation of bioTPs generally increased with the decrease of the corresponding precursors. For atenolol acid (Figure 8a), the formation was nearly linearly correlated to the relative atenolol abatement for all experiments in both columns (shown in blue and orange). By comparison, the formation of ana-TP636 and MC-TP614 dropped significantly beyond precursor abatement thresholds of about 90% and 70%, respectively (Figure 8b,c). The decrease in bioTP formation at high precursor abatement likely indicates that these bioTPs are further degraded. Similar thresholds were observed for ana-TP679 (about 90%), MC-TP885 (about 50%), MC-TP980 (about 80%), and cyanopeptolin-TP818 (about 60%), while no decreasing trend was observed for MC-TP460, MC-TP543, and gabapentin-lactam (Figures S16–18, SI1). In contrast, none of the bioTPs were formed after autoclaving, while precursors were still partially abated (black squares, Figure 8 and Figures S16–18, SI1). The bioTP formation in the bioactive sand column experiments but not in the autoclaved control supports the hypothesis that the remaining cyano-metabolite abatement after autoclaving was due to adsorption.

Additionally, the formation of ana-TP636 and ana-TP679 (bioTPs from anabaenopeptin A, anabaenopeptin B) and their subsequent abatement were also observed in the DWTP. Laboratory-scale column may therefore be representative of the full-scale sand filtration in this regard (Figure 1). Another anabaenopeptin, oscillamide Y, was present in the DWTP but not in our laboratory-scale experiments. Its expected bioTPs, i.e., following the same enzymatic reactions as anabaenopeptin A and B bioTPs, were ana-TP693 (dealkylation-like of the ureido bond) and ana-TP650 (hydrolysis at the ureido bond), both of which were also detected in the DWTP (Figure 1). Finally, expected bioTPs from [d-Asp^3^,(E)-Dhb^7^]­MC-RR were not found in the DWTP because they were either likely below the limit of detection or below the *m*/*z* cutoff used for field samples (450).

## Practical Implications

Biological sand filtration not only physically removes cyanobacterial cells but also degrades dissolved cyano-metabolites, acting as a dual-barrier process in drinking water treatment for this class of compounds. The extent of the abatement of cyano-metabolites in sand filters depends on the biological activity, operating conditions, water quality, and temperature, as well as the cyano-metabolite structures and their intake dynamic. Laboratory-scale sand filtration suggests that many cyano-metabolites are more readily biodegraded compared to benchmark synthetic micropollutants such as atenolol, paracetamol, or valsartan. Temperature and contact time greatly affect cyano-metabolite abatement. Therefore, seasonal variations in temperature have a significant effect on cyano-metabolite abatement and need to be considered when assessing their degradation. This parallels the water disinfection efficiency, which is also strongly temperature-dependent. While the temperature of the treated water is given by seasonal effects, the contact times can be adjusted within certain boundaries by operational parameters and/or the design of the sand filters. The rapid sand filters of the sampled DWTP were designed for a contact time of roughly 1 h in a normal production mode, which provided complete abatement of the cyano-metabolites despite the low temperature of 7 °C. However, higher water demand may lead to contact times lower than the design criteria, which may be insufficient for complete cyano-metabolite abatement. The risk for significant cyano-metabolite concentrations in the selected DWTP is unlikely due to low intake concentrations and the extensive multibarrier treatment (intermediate ozonation, biological activated carbon filtration, slow sand filtration) after the rapid sand filtration. Other DWTPs may, however, be exposed to more intense blooming events and/or have a less advanced treatment train. The physical removal of cyanobacterial cells as a first treatment step, especially during intense blooming events, is the best practice to prevent high aqueous-phase concentrations of cyano-metabolites from entering DWTPs. However, when substantial cyanobacterial cell lysing occurs, e.g., due to preozonation, biological sand filtration can be an efficient barrier for cyano-metabolite abatement. Its efficiency should nevertheless be evaluated case-by-case, notably to account for the dynamic patterns of cyano-metabolites in the source water. Cyano-metabolite concentrations, the rate at which bloom-related intake rises, and the potential persistence of biodegradation capacity between successive bloom events may all affect cyano-metabolite abatement and require a careful assessment. Additionally, while abating cyano-metabolites is an important requirement for water utilities, the formation of biological transformation products (bioTPs) also needs to be accounted for. Biotransformation can reduce the toxicity of cyano-metabolites, but their toxic potential may also be preserved at least in part, particularly when only minor modifications occur.^68,74^ BioTPs detected in laboratory-scale sand columns mirrored those observed under full-scale conditions, providing a valuable method to identify compounds that practitioners need to monitor. Further elucidation of biodegradation pathways and bioTP toxicity is nevertheless needed to refine risk assessment and support water utilities in managing cyano-metabolite contamination.

**Figure 8:**
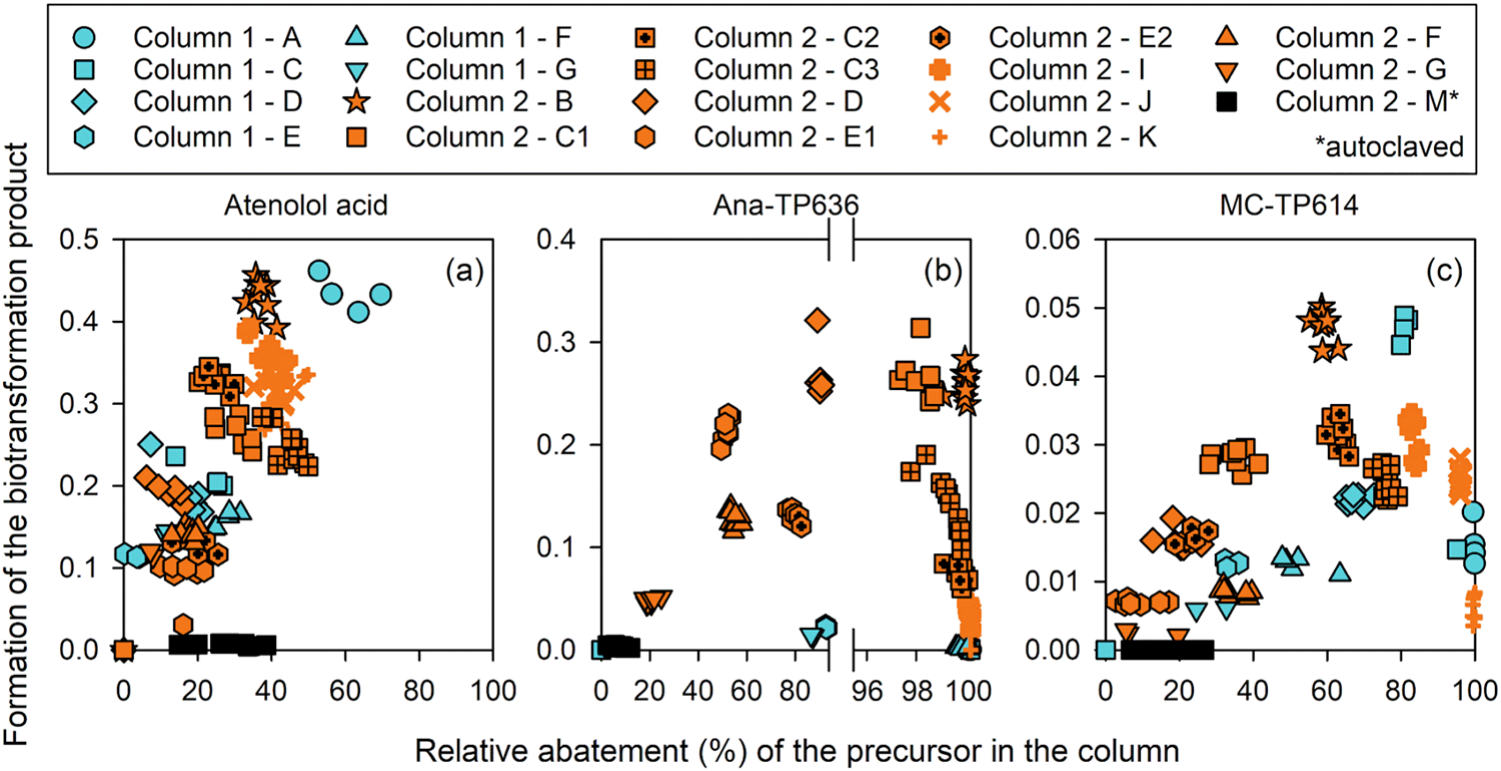
Formation of cyano-metabolite bioTPs during experiments in columns #1 and #2 as a function of precursor abatement. Formation of (a) atenolol acid, (b) ana-TP636, and (c) MC-TP614 as a function of the relative abatement of (a) atenolol, (b) anabaenopeptin A, and (c) [d-Asp^3^,(E)-Dhb^7^]­MC-RR. The areas of the product are normalized to the area of the precursor before the column. All experiments labeled from A-M are shown as individual data sets (see Table S4 for details corresponding to each experiment label, SI1).

## Supplementary Material





## Notes

Additional details on experiments and kinetic modeling, supplementary results, and information on the structure of micropollutants, cyano-metabolites, and identified bioTPs ^2^ spectra annotation (PDF)
SI2 is a spreadsheet containing the confirmation of bioTP structures by MS (XLSX)
