# Supplementary material for: Degradation of Toxins and Metabolites of Cyanobacteria and Micropollutants during Biological Sand Filtration
Source: Environ Sci Technol. 2026 Mar 16;60(12):9647–59. doi: 10.1021/acs.est.5c16532 (PMC13045012; doi:10.1021/acs.est.5c16532)
Supplement: Supplementary file 1 [file es5c16532_si_001.pdf]

SUPPORTING INFORMATION

Degradation of toxins and metabolites of cyanobacteria and micropollutants during biological sand filtration

Valentin Rougé,[a*] Anne Dax,[b] Oliver Köster,[b] Urs von Gunten,[a,c] Elisabeth M.-L. Janssen[a]*

[a] Eawag, Swiss Federal Institute of Aquatic Science and Technology, 8600 Dübendorf, Switzerland

[b] Zurich Water Supply, 8021 Zurich, Switzerland

[c] School of Architecture, Civil and Environmental Engineering (ENAC), École Polytechnique Fédérale de Lausanne (EPFL), 1015, Lausanne, Switzerland

*co-corresponding authors
phone: +41 58 765 5428, E-mail: elisabeth.janssen@eawag.ch
phone: +41 58 765 5799, E-mail: valentin.rouge@eawag.ch

This PDF includes:
7 texts, 11 tables, 3 schemes and 18 figures in 58 pages for further information addressing materials, experimental procedures and additional data.

Table of contents

Texts

Text S1. Experimental details **S5**

Text S1.1. Culture and extraction of ^{15}*N Planktothrix rubescens* **S5**

Text S1.2. Sampling of the drinking water treatment plant **S5**

Text S1.3. Extraction of particulate-bound cyano-metabolites **S6**

Text S1.4. Further details on sand column experiments **S7**

Text S2. Measurement of the pore volume and Péclet number **S8**

Text S3. Cyano-metabolite and micropollutant analysis and identification **S10**

Text S4. Kinetic modelling **S11**

Text S4.1. Apparent rate constant and activation energy determination **S11**

Text S4.2. Estimation of the effect of dispersion on kinetic rate constant and activation energy determination **S12**

Text S4.3. Comparing first-order and zero-order fitting **S14**

Text S4.4. Hierarchical clustering **S15**

Text S4.5. Apparent activation energies **S16**

Text S5. Oxygenation of aerucyclamide D and planktocyclin to aerucyclamide D-sulfoxide and planktocyclin-sulfoxide **S17**

Text S6. Identification of biotransformation products **S17**

Text S7. Identification of cyanopeptolin-TP818 .... **S19**

Tables

Table S1. Reagent suppliers and purity **S21**

Table S2. List of the 13 micropollutants used **S22**

Table S3. List of cyano-metabolites identified in *Planktothrix* and *Microcystis* extracts **S23**

Table S4. Experimental conditions used in the sand column experiments and corresponding label **S26**

Table S5. Timeline of experiments in column #1 and column #2 .... **S27**

Table S6. Average relative abatement expressed in % for each experiment conducted in column #1 .... **S28**

Table S7. Average relative abatement expressed in % for each experiment conducted in column #2 .... **S29**

Table S8. Apparent first-order rate constants *k* for the abatement of cyano-metabolites and micropollutants in the sand columns **S31**

Table S9. Activation energies Ea for the abatement of cyano-metabolites and micropollutants in the sand columns **S32**

Table S10a. Comparison of R^2 and p-value for first- and zero-order kinetic fits **S34**

Table S10b. Result of two-sided t-tests assessing the null hypothesis “intercept is significantly different from 1 (for zero-order) or 0 (for first-order)”, with a significance level α = 0.05 .. **S35**

Table S11. Identified products formed in the sand column **S37**

Schemes

Scheme S1. Scheme of the drinking water treatment train of Lengg (Zürich, Switzerland) . **S41**

Scheme S2. Structures of identified cyano-metabolites **S42**

Scheme S3. Structures of used micropollutants **S43**

Figures

Figure S1. Concentration of [D-Asp^3,(E)-Dhb^7]MC-RR entering the drinking water treatment plant Lengg between 2010 and 2024 .... **S43**

Figure S2. Fitting of the salt tracer breakthrough for column #1 (a) and column #2 (b) **S44**

Figure S3. Representation of the analogy between batch reactor and column experiments . **S44**

Figure S4. Examples of correlations used to calculate first-order k in column #1 and #2 ... **S45**

Figure S5. Examples of correlations used to calculate Ea in column #1 and #2 .... **S45**

Figure S6. Simulation of concentration first-order concentration decrease across the sand column for ideal and non-ideal plug-flow systems **S46**

Figure S7. Examples of simulation of ln(C/C_0) vs time plots for ideal and non-ideal plug-flow systems **S46**

Figure S8. Simulated effect of non-ideal plug flow on first-order linear regression **S47**

Figure S9. Simulated effect of non-ideal plug flow on zero-order linear regression **S47**

Figure S10. Effect of flow rate on the relative abatement of cyano-metabolites (a) and micropollutants (b) in laboratory sand column #1 .... **S48**

Figure S11. Effect of the temperature on the relative abatement of cyano-metabolites (a) and micropollutants (b) in laboratory sand column #1 .... **S49**

Figure S12. Effect of exposure time on the abatement of cyano-metabolites (a) and micropollutants (b) in column #2 .... **S50**

Figure S13. Relative abatement of selected cyano-metabolites and micropollutants at fixed cyano-metabolite concentration and varying micropollutant concentrations........ **S51**

Figure S14. Relative abatement over time of all compounds before and after autoclaving in column #2 .... **S52**

Figure S15. Peak area of the sulfoxide form of aerucyclamide D and planktocyclin against the peaks area of corresponding non-oxidized cyano-metabolite **S53**

Figure S16. Formation of gabapentin-lactam (a) and ana-TP679 (b) as a function of the relative abatement of their precursor........ **S54**

Figure S17. Formation of MC-TP460 (a) and MC-TP980 (b) as a function of the relative abatement of [D-Asp^{3},(E)-Dhb^{7}]MC-RR........ **S55**

Figure S18. Formation of cyanopeptolin-TP818 as a function of the relative abatement of cyanopeptolin D **S56**

Text S1. Experimental details

Text S1.1. Culture and extraction of ^{15}N Planktothrix rubescens

A batch of *Planktothrix rubescens* was cultured in the same conditions as previously described,[1] but replacing $Na(^{14}NO_3)$ with $Na(^{15}NO_3)$ in the medium and the biomass was harvested after three inoculation cycles before extraction and semi-purification by semi-preparative-HPLC. HPLC-fractions were concentrated by vacuum-assisted evaporation (Syncore® Analyst R-12, BÜCHI Labortechnik AG, 40°C, 120 rpm, 20 mbar), resuspended in ethanol and stored at -20°C to generate a ^{15}N-labeled standard mixures of cyano-metabolites used as an internal standard for the sand column experiments. Complete labeling of all nitrogen atoms in the cyanopeptides was achieved and no unlabeled peptides with (i.e., with only ^{14}N) remained as verified by LC-MS analysis.

Text S1.2. Sampling of the drinking water treatment plant

Due to the mixing of Lake Zürich over the winter and a water intake depth at 30 m, cyanobacteria typically reach the drinking water treatment plant (DWTP) in January at a temperature of 7°C (see the seasonal cyano-metabolite intake over the period 2010-2024 in Figure S1, SI1). The DWTP has a primary ozonation step followed by rapid sand filtration, an intermediate ozonation followed by activated carbon filtration and a slow sand filtration (see Scheme S1). Around 0.5 and 0.3 $mgO_3\ L^{-1}$ was dosed in the primary and intermediate ozonation, respectively, on the day of sampling. Grab samples were taken in glass Schott bottles of the raw water (inflow) and after each step following the treatment train by roughly accounting for the corresponding hydraulic residence time of each treatment step. In addition, several grab samples were taken across the depth of one of the rapid sand filters. Samples after ozonation steps were quenched with 50 µM buten-3-ol. All samples were directly filtered on site through 0.7 µm Whatman® GF/F glass fiber filters to prevent the leaching of cyano-metabolites from damaged cells. Glass fiber filters were then extracted to obtain the particulate-phase cyano-

metabolite concentrations. Filtrates and filters were stored at -20°C until their extraction (for glass fiber filters) and analysis. Raw water at the inflow was sampled again in February 2024 and January 2025 for the laboratory column experiments. The pH of raw water ranged between 8.3-8.6 and was not significantly affected by ozonation nor by cyano-metabolite and micropollutant spiking. The dissolved organic carbon content was 1.5 ± 0.1 mgC L^{-1} for the raw lake water, 1.2 ± 0.2 mgC L^{-1} for the ozonated lake water and 1.5 ± 0.1 mgC L^{-1} for the spiked ozonated lake water. The sand used in this study was sampled from the quartz sand layer of one of the rapid sand filter beds of the same DWTP. Sand filters consist of a top layer of pumice stone (about 50 cm in depth, with a grain diameter of around 2-3 mm) followed by a layer of quartz sand (about 80 cm in depth, with grain diameter of around 0.7-1.2 mm). The sand filter had been in operation for at least 10 years and preventively backflushed every two weeks. The vertical velocity of the sand was 1.2 m h^{-1} (i.e., around 1.1 h contact time) on the day of sampling and can vary between 1.2 – 6 m h^{-1} depending on the production level. The sampled sand was kept in tap water in an open container at 4°C until being used for two experimental batches, 5 and 11 months later.

Text S1.3. Extraction of particulate-phase cyano-metabolites

The glass fiber filters were extracted by adding 10 mL of 70:30% v/v methanol/nanopure water solution and vortex mixing (Vortex Genie 2) for 10 s, followed by agitation in an ultrasonic water bath (VWR Ultrasonic Cleaner USC-THD) operated at 40 °C and at maximum power for 30 min. The extracts were then centrifuged for 10 min at 4000 g at room temperature (Heraeus Megafuge 1.0), and 8 mL of the extract were transferred into a glass vial. This extraction procedure was repeated, and the resulting extract was combined with the first round of extraction. The 16 mL extracts were concentrated at 40 °C under a gentle stream of nitrogen gas (0.5–3.0 L min^{-1} over a 2 h ramp) using a Turbovap LV (Biotage, Sweden) to remove most of the methanol. After the evaporation, when approximately 30% of the initial volume remained, the solution was transferred to a new glass vial and gravimetrically adjusted

with nanopure water to approximately 5 g (exact masses were noted). These biomass extracts were stored at −20 °C. Biomass extracts and aqueous samples were freshly thawed and centrifuged for 10 min at 4000 g at room temperature (Heraeus Megafuge 1.0) and diluted with nanopure water (10 to 500-fold) before analysis.

Text S1.4. Further details for sand column experiments

Raw water was ozonated with 0.5 mgO_3 L^{-1} (0.3 gO_3 $gDOC^{-1}$) before being fed to the laboratory-scale sand filter columns within one week of ozonation. The conditioning flow rates for column #1 (0.25 mL min^{-1}) and column #2 (0.5 mL min^{-1}) corresponded to the slowest flow rate used in the respective column experiments.

To spike the ozonated lake water, aliquots of cyanobacteria extracts stored in 85:15 methanol:water were evaporated to dryness under vacuum (Syncore Analyst R-12, BÜCHI Labortechnik AG, 55°C, 60 rpm, 80 min at 150 mbar, 20 min at 90 mbar, and 80 min at 20 mbar) to ensure complete removal of methanol. *Planktothrix rubescens* K-0576 was chosen as it is the dominant strain in Lake Zürich and *Microcystis aeruginosa* PCC7806 was selected because it produces globally-relevant cyano-metabolites that differ from those of Planktothrix rubescens, notably additional microcystins, cyanopeptolins and cyclamides. The extracts were then redissolved in the ozonated lake water to which micropollutants were then added. The micropollutants were prepared directly in ultrapure water (Arium Pro, Sartorius, 18.7 MΩcm). They were selected based on three factors: their known biodegradability (either in sand filtration or wastewater treatment), their frequent detection in the DWTP selected for this study, and their measurability in one analytical method together with cyano-metabolites. Carbamazepine, acesulfame and sucralose were selected as recalcitrant, diclofenac as a moderately biodegradable to recalcitrant compound, valsartan and tramadol as moderately biodegradable, paracetamol, molinate and atenolol as readily biodegradable.[2-4] Triclosan was also added and despite having some biodegradability, its removal is expected to be governed by sorption.[4, 5]

The individual concentrations of micropollutants were based on their sensitivities during analysis. Micropollutant concentrations ranged from 0.1-0.6 µg L^{-1} (for the lowest concentration range) to 0.6-6.3 µg L^{-1} (for the highest concentration range). The concentrations of cyanobacteria extracts were equivalent to 1.2-12.3 $mg_{biomass}$ L^{-1} of *Planktothrix rubescens* and 0.69-6.9 $mg_{biomass}$ L^{-1} of *Microcystis aeruginosa* (1.9-19.2 $mg_{biomass\text{-}equivalent}$ L^{-1} total). The list of all micropollutants, identified cyano-metabolites and their concentrations are shown in Tables S2 and S3. Compound structures are shown in Schemes 1 (main manuscript), S2 and S3.

Experiments were conducted at various flow rates (0.25-4.0 mL min^{-1}), temperatures (4-21°C) and spiked concentrations (see Table S4 for details). The resulting velocity range (0.03-0.5 m h^{-1} for 0.25-4 mL min^{-1}) was closer to a slow sand filter than a rapid sand filter.[6] This range was however selected to minimize the required experimental volumes and, consequently, the amount of cyanobacterial extract used, as well as the pressure limitation of the Omnifit glass column. Each experiment lasted from 4-7 h and included an equilibration period, which corresponded to the time needed for 10 bed volumes to pass through the column (e.g., 2 h at 1 mL min^{-1}). After the equilibration, samples were taken every 0.5 or 1 h during a 3-4 h period (between 3 and 6 sampling points) to which internal standards for micropollutants and ^{15}N-labeled standard mix of cyano-metabolites were spiked and analyzed within 24 h (see Tables S2 and Table S3, for the list of internal standards). At the end of each experiment, the column was fed with ozonated lake water without cyano-metabolites/micropollutants until the next experiment. Sample filtration before analysis was evaluated on samples with 0.45 µm PTFE filters (Merck) but was then avoided because of suspected adsorption of some analytes. The stability of samples without filtration was tested and no change in concentrations was observed for any of the analytes over 48 h until analysis.

Text S2. Measurement of the pore volume and Péclet number

NaCl was used as a non-reactive salt tracer to evaluate the pore volume as well as Peclet number of the columns.[7] The breakthrough curves, shown as C/C_0 as a function of time in Figure S2, reflect the transport behavior of the tracer through the porous medium. To analyze the flow characteristics, the experimental data were fitted using the analytical solution of the one-dimensional advection–dispersion equation (ADE):[7]

$$\frac{dC}{dt} + u\frac{dC}{dx} = D\frac{d^2C}{dx^2} \qquad \text{Eq(S1)}$$

With *u* pore velocity (m s^{-1}) and *D* the axial dispersion coefficient (m^2 s^{-1}) defined as follows:

$$u = \frac{Q}{A\times\varepsilon} \qquad \text{Eq(S2)}$$

$$D = \frac{u\times L}{Pe} \qquad \text{Eq(S3)}$$

With *Q* the volumetric flow rate (m^3 s^{-1}), A the cross-section area (m^2), ε the porosity, *L* the total length of the column (m) and *Pe* the Péclet number. This model accounts for both convective transport and axial dispersion and predicts the outlet concentration as a function of time based on two key parameters: the porosity (ε) and the Péclet number (*Pe*). The model fitting was performed by minimizing the squared difference between the experimental and predicted concentrations using nonlinear optimization, allowing simultaneous estimation of ε and *Pe*. The pore volumes were then calculated by multiplying the empty bed volume by the porosity. The fitting is shown in Figure S2. The fitted ε values were similar in the two columns, 0.44-0.45, and are within the expected range for sand.[8] The resulting pore volumes are 11.2 and 10.7 mL for column #1 and #2, respectively. *Pe* for column #1 and #2 was 13 and 23, respectively. This indicates that the advection (vertical transport via the fluid movement) is dominant in these columns but some dispersion (spreading and mixing of the solute) remains, making the columns non-ideal plug flow systems.

Text S3. Cyano-metabolite and micropollutant analyses and identification

Cyano-metabolites were analyzed by HPLC (Dionex Ultimate 3000 RS pump, Thermo Fischer Scientific) with Atlantis T3 C18 column (3 µm, 3.0 × 150 mm, with the corresponding VanGuard® precolumn, Waters), coupled to a high-resolution tandem mass spectrometer (HRMS/MS, Exploris, ThermoFisher Scientific). 100 µL samples were injected and eluted using nanopure water and MeOH, both acidified with formic acid (0.1%). A gradient elution was carried out at a flow rate of 0.3 mL min^{-1}: MeOH at 2% for 1.5 min, increasing to 95% between 1.5 and 18.5 min, and kept at 95% between 18.5 and 30.5 min before returning to 2%. For the last experiments (experiments G, F, C3 and M in column 2, see Table S4), this elution was shortened as follows: MeOH at 2% for 1 min, increasing to 95% between 1 and 16 min, and kept at 95% between 16 and 23 min before returning to 2%. HRMS/MS used electrospray ionization (ESI) with 320°C capillary temperature, 275°C vaporizer temperature and both positive and negative ionization modes with 3.5 kV and 2.5 kV electrospray voltage, respectively. Full scan from *m/z* 100 to 1100 was used with a nominal resolution of 120000 at *m/z* 250. Internal mass calibration was done on each run start (EASY-IC) to prevent mass drift overtime. Top 3 data-dependent acquisition high-resolution product ion spectra were obtained by normalized collision energies for HCD of 30% at a resolving power of 15000 at *m/z* 200, 1 *m/z* isolation window and 2s dynamic exclusion time. A slight variation was made for a method used for transformation product screening, with a top 6 data-dependent acquisition, a dynamic exclusion of 4s and a list exclusion of 25 *m/z* present at significant intensity. Skyline 22.2 (MacCoss LabSoftware) was used for the integration of the peaks. Within the software, MS^1 filtering settings were the same as the measurement settings, i.e., *m/z* scan range between 110 and 1100, and resolving power of 120,000 at *m/z* 250. The cumulated integration of the peak areas of up to M+3 isotopes was used. The internal standards used are indicated in Table S2 for micropollutants and Table S3 for cyano-metabolites. For micropollutants, most of the internal standards were the isotopically label equivalent, except for molinate and triclosan for which

atorvastatin-d5 and valsartan-13C5,15N were used, respectively. The cyano-metabolites, anabaenopeptin A, anabaenopeptin B and [D-Asp^3,(E)-Dhb^7]MC-RR had their ^{15}N-isotopically labelled equivalent extracted from a ^{15}N-labelled *Planktothrix rubescens* biomass (see Text S1.1). For the other cyano-metabolites as well as molinate and triclosan, various labelled micropollutants or cyano-metabolites were used (see Tables S2 and S3). In this case, the internal standard was selected based on the best correlation between internal standard and cyano-metabolite peak areas across multiple spiked raw lake water samples.

Text S4. Statistical analyses

Text S4.1. Determination of apparent rate constants and activation energies

Under the steady-state assumption, enzymatic reactions can be described by the Michaelis-Menten equation:

$$v = \frac{V_{\mathrm{max}} \times \mathrm{C}}{K_{\mathrm{m}} + \mathrm{C}} \qquad \text{Eq(S4)}$$

With v the reaction rate, V_{max} the maximum reaction velocity, K_{m} Michaelis constant and C the concentration of the substrate. When C is low enough (<< K_{m}), i.e., well below the saturation point of the enzyme, the enzymatic reaction follows a first-order kinetic:[9]

$$\ln\left(\frac{\mathrm{C}}{\mathrm{C_0}}\right) = -k\mathrm{t} \qquad \text{Eq(S5)}$$

With k the first-order rate constant, C_0 the initial substrate concentration and t the time.

Conversely, when C is high enough (>> K_{m}), the enzymatic reaction rate becomes independent from C and the reaction follows a zero-order kinetic:

$$\frac{\mathrm{C}}{\mathrm{C_0}} = -\frac{k}{\mathrm{C_0}}\mathrm{t} \qquad \text{Eq(S6)}$$

In the context of column experiments, the time corresponds to the average residence time of a compound at a given flow rate calculated from the pore volume (Text S2), and C and C_0 to the substrate concentrations at the outlet and inlet, respectively (a representation of the analogy

between plug-flow reactor and our column experiments is shown in Figure S3 for first-order). To use Eq(S5) or Eq(S6), the sand columns were considered as ideal plug-flow reactors. This is in practice not the case as axial dispersion occurs, indicated by Péclet numbers of 13 and 23 for column #1 and #2, respectively (see details in Text S2). The effect of the dispersion on kinetic fitting was evaluated for both first and zero-order and deemed minimal for our purpose (see Text S4.2). The statistical comparison between first- and zero-order for best fit of the experimental data is discussed in Text S4.3. Apparent first-order rate constants (k_{app}) were then obtained from the linear regressions of ln(C/C_0) vs time using Eq(S5) (see examples in Figure S4 and values in Table S8). In addition to k_{app}, the effect of the temperature on enzymatic reactions can be quantified through the calculation of the activation energy (E_a) of a given reaction by the Arrhenius equation via Eq(S7):[9]

$$\ln(k) = \ln(A) - \frac{Ea}{R} \times \left(\frac{1}{T}\right) \qquad \text{Eq(S7)}$$

With R the universal gas constant (8.314 J mol^{-1} K^{-1}), T the temperature (in K) and A the pre-exponential factor. The Arrhenius equation is expected to be valid for enzymatic reactions within the temperature range used herein.[10] Here, due to limited available datasets, k could not be measured from the linear regression of Eq(S7). However, by rearranging Eq(S7), ln(C/C_0) can be directly correlated to 1/T for a fixed contact time:

$$ln\left(-ln\left(\frac{C}{C_0}\right)\right) = -\left(\frac{Ea}{R}\right) \times \frac{1}{T} + \ln(A) + \ln\,(t) \qquad \text{Eq(S8)}$$

Correlation examples are provided in Figure S5 and E_a values are given in Table S9.

Text S4.2. Estimation of the effect of dispersion on rate constant

For the determination of degradation kinetics, the sand columns were considered as ideal plug-flow systems, which allows using Eq(S5) and Eq(S6). In reality, the sand columns are not ideal plug flow systems and significant dispersion likely occurs, quantified here by Péclet

numbers of 13 and 23 for column #1 and #2, respectively (see Text S2). Examples of the effect of dispersion on the concentration profile through our columns considering a first-order abatement kinetic is simulated in Figure S6. The simulation is based on the steady-state advection–dispersion–reaction equation:[11]

$$u\frac{dC}{dx} = D\frac{d^2C}{dx^2} - kC \qquad \text{Eq(S9)}$$

With u the pore velocity (m s^{-1}), D the axial dispersion coefficient (m^2 s^{-1}) calculated from the Péclet number, and k the apparent first-order rate constant (s^{-1}). The dispersion is expected to inhibit the observed abatement, although no strong effect should be observed for Péclet numbers > 10 (Figure S6). For further evaluation, the effect of dispersion on k_{app} determination was also modelled. Several linear regressions of $\ln(C/C_0)$ vs t were simulated for different flow rates (i.e., different pore contact time), and at different Péclet numbers using Eq (S6) and (S9). The resulting simulated $\ln(C/C_0)$ vs time plots were used to obtain the corresponding k_{app} (examples are shown in Figure S7). Then, the R^2, k_{app} and y-intercept obtained for non-ideal plug-flow systems were compared to the ideal system (Figure S8). R^2 remained high even at low Pe (i.e., high dispersion) ($R^2 > 0.98$) and high k_{app}, which leads to the highest deviation from linearity (Figure S8a). For $Pe > 10$, k_{app} was underestimated by less than 40% for a k_{app} of 0.01 s^{-1}, and less than 20% for a k_{app} of 0.001 s^{-1} (Figure S8b). This variation is reasonable considering the typical variation of rate constants, and the complexity of the sand column system. Additionally, the ideal plug-flow approximation can lead to a slight negative shift of the y-intercept (Figure S7a). Treating the column as a batch reactor remains a large approximation due to other potential processes that could not be evaluated here such as the non-uniformity of the system or sorption/desorption processes.

The effect of dispersion on zero-order fitting was also evaluated by using Eq(S6) and Eq(S10):

$$u\frac{dC}{dx} = D\frac{d^2C}{dx^2} - k \qquad \text{Eq(S10)}$$

Unlike for first-order, the ideal plug-flow approximation is not expected to affect linearity nor y-intercept (Figure S9).

Text S4.3. Comparing first-order and zero-order fittings

The fitting of the experimental data set was compared between the zero-order and first-order fitting using two parameters: R^2 and y-intercept. R^2 were compared by calculating the difference between zero and first-order (Table S10a) while the y-intercept was compared by testing whether it was significantly different from the expected y-intercept (1 and 0 for zero- and first-order, respectively, see Eq(S5) and Eq(S6)) using a two-sided t-test with a significance level $\alpha = 0.05$.

R^2 for first-order were higher than for zero-order for 13 and 11 compounds in columns #1 and #2, respectively (Table S10a). The rest of the compounds i.e., 7 and 3 compounds for columns #1 and #2, respectively, had similar R^2 for both orders (< 0.01 difference). For the y-intercepts, it is worth noting that the plug-flow approximation is expected to lead to a slight negative shift of the y-intercept for first-order (Figure S7), which was consistently observed for all compounds with $R^2 > 0.8$ (y-intercept values not shown). Conversely, no deviation from the expected y-intercept is expected for zero-order (Text S4.2). The statistical test results are shown in Table S10b only for compounds that showed good correlations ($R^2 \geq 0.8$). A "FALSE" indicates that the intercept is not significantly different from the expected y-intercept and hence hints at a better fit. A limitation of this method is that both zero- and first-order may yield a FALSE or a TRUE. In 20 out of 34 tests both first- and zero-order yielded the same statistical response while 13 tests concluded that only first-order led to a non-significant y-intercept. Another way to compare y-intercept is to calculate the ratio of the *p*-value between the zero- and first-order. A higher *p*-value indicates a less significant difference from the expected y-

intercept, and therefore a better fit. In column #1, a higher *p*-value was found for first-order for all compounds but atenolol, gabapentin, molinate and paracetamol, for which *p*-values were within a factor 10 for both zero- and first-order (Table S10b). In column #2, anabaenopeptins and cyanopeptolins had a higher *p*-value for first-order, except cyanopeptolin 963A. For microcystins, [D-Asp^3]MC-LR had a higher *p*-value with zero-order while they were the same for both orders for [D-Asp^3,(E)-Dhb^7]MC-RR and higher with first-order for [D-Asp^3,Dha^7]MC-RR (MC-LR was not tested because of a poor correlation). Finally, atenolol had a higher *p*-value with first-order while they were within an order of magnitude for molinate. Overall, these results suggest that first-order better represent the abatement of most compounds. It cannot be excluded that a mixed order occurs, and that the complexity of the column system introduced a bias in these statistical tests.

Text S4.4. Hierarchical clustering

Hierarchical clustering was performed on compounds based on Euclidean distances using the average-linkage (UPGMA) algorithm, a classical method originally introduced in numerical taxonomy.[12] Pairwise Euclidean distances are calculated across all experiments for each compound pair using Eq(S11):

$$d(\text{compound}_1, \text{compound}_2) = \sqrt{\sum_{j=1}^{y}\left(\text{abatement}_{1,\text{j}} - \text{abatement}_{2,\text{j}}\right)^2} \quad \text{Eq(S11)}$$

In Eq(S11), compound 1 and compound 2 are two given compounds of our dataset and j represents one of the y experiments conducted. Experiments with incomplete dataset (compounds below limit of detection) were removed, i.e., experiment J1, J2, K and L (see Table S7). Additionally, micropollutants without significant abatement across all experiments were removed from the analysis (i.e., acesulfame, carbamazepine, diclofenac, lamotrigine, sucralose, tramadol and valsartan acid). Once all Euclidian distances are calculated for all compound pairs, UPGMA merges compounds or clusters based on the average of all pairwise distances between

them. Compounds with smaller Euclidean distances across experiments are merged first, indicating similar abatement patterns, while more dissimilar compounds merge at higher Euclidean distance. The resulting hierarchical relationships are visualized as a dendrogram (Figure 6). To assess the statistical robustness of the hierarchical clustering of compounds, multiscale bootstrap resampling (10,000 iterations) was performed using the pvclust package in R.[13] This approach repeatedly reruns UPGMA on randomly resampled subsets of the data (i.e., using part of the compounds) and evaluates how often each cluster observed in the original dataset is reproduced. The sampling size varies from 50% to 140% of the real dataset and a same compound dataset may appear multiple times. The frequency with which a cluster appears across these resampled datasets is reported as the approximately unbiased (AU) *p*-value. Clusters that consistently appear across these resampled datasets are considered statistically robust, with AU *p*-values ≥ 95% typically indicating strong support and AU p-values between 90-94% indicating moderate support.

Text S4.5. Apparent activation energies

The effect of the temperature was also quantified via the calculation of apparent activation energies (E_a) (see details on their determination in Text S4.1). Large discrepancies were however found between column #1 and #2 (Table S9). While *Ea* values determined in column #1 were consistent with the typical range of E_a expected for enzymatic reactions (20-60 kJ mol^{-1}),[9, 14] those determined in column #2 were significantly higher. The calculated apparent E_a should be interpreted with caution due to limited experimental data (more temperature points and a proper determination of k_{app} at each temperature would be required, see Text S4.1) and the complexity of the column system. Beyond the intrinsic enzymatic reaction *Ea*, other processes such as sorption/desorption or enzyme availability may have contributed to the observed temperature effect.

Text S5. Oxygenation of aerucyclamide D and planktocyclin to aerucyclamide D-sulfoxide and planktocyclin-sulfoxide

Sulfoxide forms of aerucyclamide D and planktocyclin were initially present in the mixture due to the easy oxygenation of methionine during cyanobacteria cultivation and/or extraction. Before autoclaving, the peak areas of sulfoxide forms were consequently decreasing in the column, alongside the peak area of their precursors (black bars, Figure S15). Conversely, the peak areas of these sulfoxide forms increased in the column after autoclaving (white bars, Figure S15). This data suggests that aerucyclamide D and planktocyclin are rapidly oxygenated to sulfoxide in the column, and that before autoclaving the sulfoxide form can be further (bio)degraded. Before autoclaving, the simultaneous formation and degradation of the sulfoxide forms likely occurred, with the degradation being more prominent. It is important to note that while planktocyclin abatement was similar before and after autoclaving, the abatement of aerucyclamide D was increased by autoclaving from 44 ± 7% to > 90%. This increase may suggest that the abiotic oxygenation in the column of methionine was enhanced after autoclaving, although the reason is unknown.

Text S6. Identification of biotransformation products

For atenolol, triclosan, valsartan, molinate, gabapentin, paracetamol, anabaenopeptin A, anabaenopeptin B, [D-Asp^3,Dhb^7]MC-RR , [D-Asp^3]MC-LR and MC-LR, the biodegradation products (bioTPs) found in the literature were used as suspect list.[15-21] For microcystins, more bioTP suspects were added based on expected pathways, i.e., peptide hydrolysis products. For cyanopeptolins, no bioTP have yet been reported and expected products from arginase or esterase were not found. Therefore, further efforts for bioTP identification were made. The identification process consisted in selecting compounds that were formed in the column and

containing one or more MS^2 fragments characteristic of cyanopeptolins. No effort was made for the identification of cyclamide bioTPs. Altogether, 7 bioTPs previously reported were identified (atenolol acid, gabapentin lactam, MC-TP980, MC-TP614, MC-TP543, Ana-TP679 and Ana-TP636),[15, 21, 22] and 3 new bioTPs were identified herein for the first time (MC-TP885, MC-TP460 and cyanopeptolin-TP818). The product information, structure and identification confidence level are given in Table S11. The confidence level is based on a universal system going from 1 (highest confidence, confirmed by a standard) to 5 (lowest confidence, only exact mass) that describes the level of identification of a given compound.[23] Compound annotation details are given in a separate spreadsheet (SI2). Atenolol acid, MC-TP614, MC-TP460, MC-TP543 and MC-TP885 result from amide hydrolysis, gabapentin- lactam from intramolecular cyclization and dehydration, MC-TP980 from guanidine degradation by arginase followed by hydrolysis,[24] ana-TP679 from a dealkylation-like reaction at the ureido bond and ana-TP636 via hydrolysis of the ureido bond.

MC-TP614, MC-TP543 and MC-TP460 are consistent with the enzymatic pathway previously suggested for MC-LR, i.e., linearization of the microcystin through hydrolysis of the amide bond between adda and arginine, followed by further degradation of the linear peptide via further amide bond hydrolysis.[15] MC-TP980 and MC-TP885 suggest that other pathway can also occur for [D-Asp^3,(E)-Dhb^7]MC-RR, i.e., arginine degradation before peptide linearization (MC-TP980) and peptide linearization at a different site of the microcystin (MC-TP885), i.e., either at the amide bond between the aspartic acid and the arginine, or between the arginine and the alanine (see SI2 for detailed structure identification).

Other potential products were detected for valsartan, gabapentin, molinate, cyanopeptolins and microcystins, but without good enough MS^2 information they could not be confirmed and therefore are not reported. For cyanopeptolin-TP818, a tentative structure is given based on MS^1 and MS^2 information (see Text S7).

Text S7. Identification of cyanopeptolin-TP818

Cyanopeptolin-TP818 was primarily detected as $[M\text{-}H_2O+H]^+$, i.e., *m/z* 801.4395 while $[M+H]^+$ (*m/z* 819.4503) was also detected but with a signal 50 times lower:

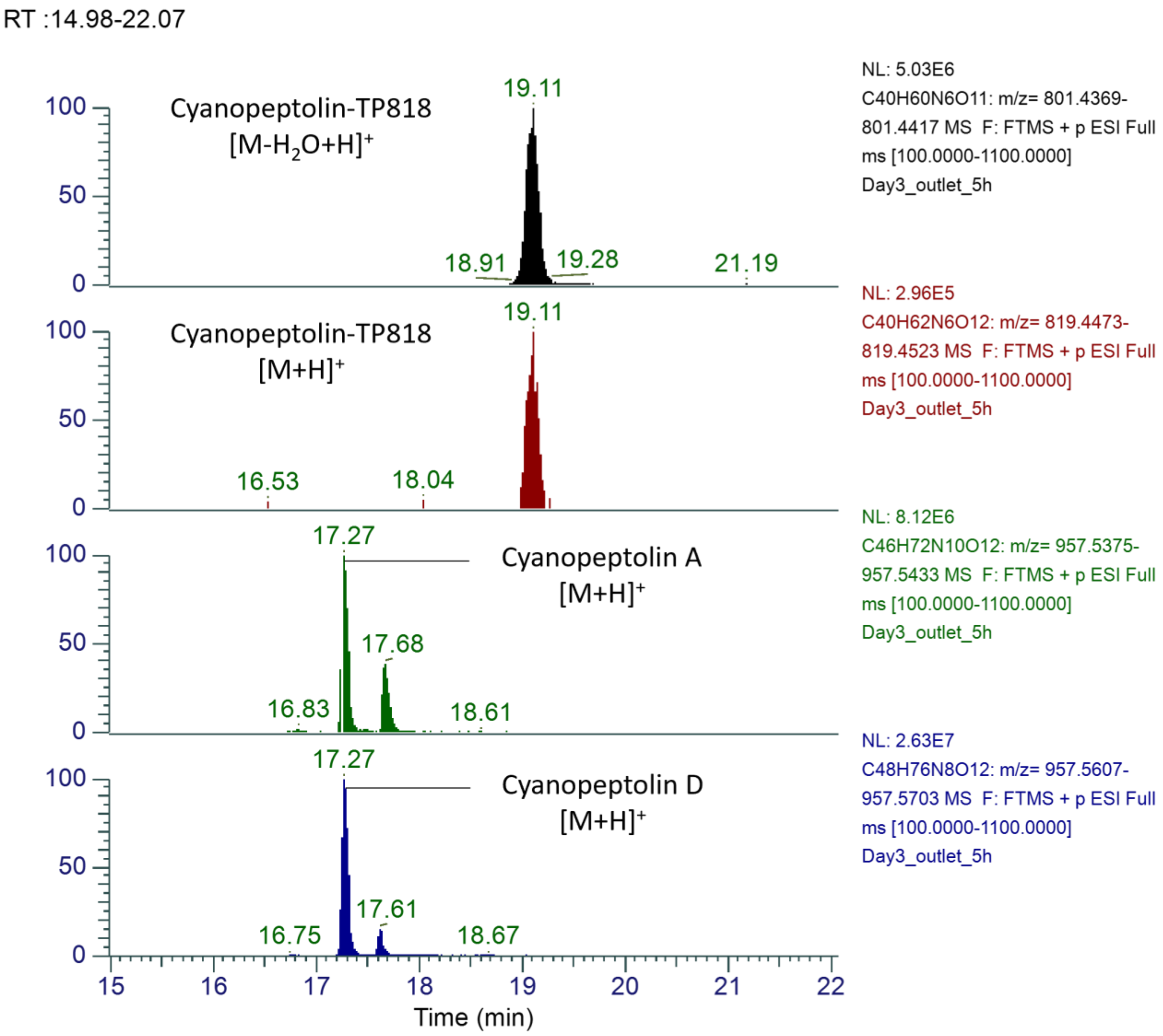

The exact mass measured across 49 samples for $[M\text{-}H_2O+H]^+$ was 801.4395 ± 0.0003 and corresponds to $C_{40}H_{61}N_6O_{11}$, with a -0.27 ppm shift. Other molecular formulas within 1 ppm mass error were all excluded for having more C or more N than cyanopeptolin precursors. The tentative molecular formula for $[M+H]^+$ was therefore $C_{40}H_{63}N_6O_{12}$, which has a theoretical *m/z* of 819.4498, < 1 ppm difference from the measured *m/z*. The $[M+H]^+$ tentative

molecular formula corresponds to a net loss of $C_6H_{10}N_4$ from cyanopeptolin A, or $C_8H_{14}N_2$ from cyanopeptolin D (the two main cyanopeptolins).

The loss of water, while not occurring extensively in MS^1, is also very prominent in all cyanopeptolins during MS^2 fragmentation (see cyanopeptolin A-D annotation in reference [25]). This suggests that water loss is further facilitated in cyanopeptolin-TP818 compared to the precursor, leading to almost complete in-source fragmentation. Additionally, the +1.8 min retention time shift of cyanopeptolin-TP818 compared to cyanopeptolin A-D (they all elute at the same retention time) suggests an increased hydrophobicity.

The MS^2 of $[M-H_2O+H]^+$ of cyanopeptolin-TP818 was acquired at hcd30 and compared to the $[M+H]^+$ MS^2 of cyanopeptolin A at hcd45 to identify its structure (see details in SI2). The tentative structure corresponds to the loss of the arginine residue via the hydrolysis of the amide bonds:

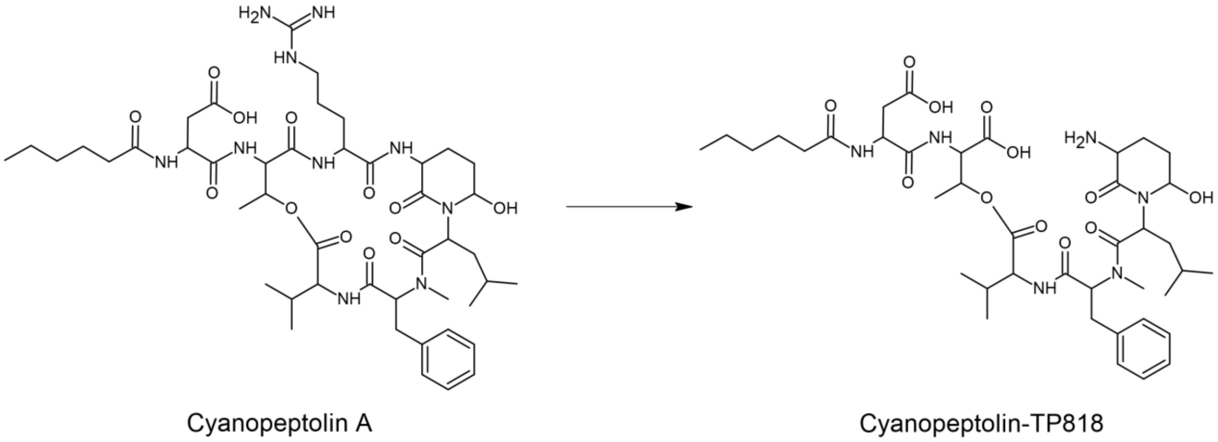

The loss of arginine was also observed in another newly identified product MC-TP885 (Text S6). As the arginine residue is the only part that changes to lysine derivatives in cyanopeptolins B-D (Scheme S2), cyanopeptolin-TP818 could in theory be formed from either of the cyanopeptolins A-D. The formation of an additional carboxylic acid may explain the facilitated H_2O loss of cyanopeptolin-TP818 compared to its precursor(s) as it is a known moieties undergoing facile H_2O loss.[26] Additionally, the loss of the arginine (for cyanopeptolin A) or lysine derivative (for cyanopeptolin B-D) may explain the observed retention time shift.

Predicted logD (via Chemaxon JChem software) of cyanopeptolin-TP818 is 0.5-1 unit higher than those of cyanopeptolin A-D at pH 2, i.e., the pH of the HPLC eluent.

It is important to note that cyanopeptolin-TP818 is tentative and that a better MS^2 fragmentation pattern would be required to confirm its structure. Although the cleavage of the ester bond would be expected to be a primary site of enzymatic attack, no modification of cyanopeptolin A-D starting by the ester hydrolysis could lead to cyanopeptolin-TP818 tentative molecular formula without extensive molecular reorganization. Ester hydrolysis may still occur as a primary pathway while its product not identified.

Table S1. Reagent suppliers and purity

Chemical	Supplier, purity	Chemical	Supplier, purity
Methanol	Fischer scientific, Optima LC-MS grade	Acesulfame	Sigma-Aldrich (99.9%) As acesulfame potassium
Formic acid	Sigma Aldrich, ≥ 98%	Atenolol	TCI Europe (98%)
Microcystin-LR	Enzo life science, >95% (HPLC area)	Carbamazepine	TCI Europe (97%)
[D-Asp^3]Microcystin-LR	Enzo life science, >95% (HPLC area)	Diclofenac	Sigma-Aldrich (99.9%) as diclofenac sodium salt
[D-Asp^3,(E)-Dhb^7]Microcystin-RR	CyanoBiotech GmbH, >95% (HPLC area)	Gabapentin	TRC Canada (99.5%)
Cyanopeptolin A	CyanoBiotech GmbH, >90% (HPLC area)	Lamotrigine	TCI Europe (98%)
Cyanopeptolin D	CyanoBiotech GmbH, >90% (HPLC area)	Molinate	Fluka (97%)
Anabaenopeptin A	CyanoBiotech GmbH, >90% (HPLC area)*	Paracetamol	Sigma-Aldrich (99%)
Anabaenopeptin B	CyanoBiotech GmbH, >90% (HPLC area)*	Sucralose	TCI Europe (98%)
Oscillamide Y	CyanoBiotech GmbH, >90% (HPLC area)	Tramadol	Sigma-Aldrich (99.9%) as tramadol hydrochloride
		Triclosan	HPC Standards GmbH (99.9%)
		Valsartan	TCI Europe (98%)
		Valsartan acid	TRC Canada (96%)

*Standardized by UV absorbance.[27]

Table S2. List of selected micropollutants. Concentrations varied depending on the experiments (see Table S4). The higher range is the range used in most experiments (experiments A-H, L and M).

Compound Name	CAS #	Molecular Formula	Retention time (min)	Ion precursors monitored	Internal standard used	Concentration range used (μg L^{-1}) / (nM)
Acesulfame	33665-90-6	C4H5N1O4S1	9.2	$[M-H]^-$	Acesulfame-D4	0.24-2.41 / 1.5-14.8
Atenolol	29122-68-7	C14H22N2O3	8.3	$[M+H]^+$	Atenolol-D7	0.13-1.25 / 0.5-4.7
Carbamazepine	298-46-4	C15H12N2O	17.3	$[M+H]^+$	Carbamazepin-D8	0.13-1.25 / 0.5-5.3
Diclofenac	15307-86-5	C14H11Cl2N O2	20.3	$[M+H]^+$/$[M-H]^-$	Diclofenac-D4	0.21-2.09 / 0.7-7.1
Gabapentin	60142-96-3	C9H17NO2	10.2	$[M+H]^+$	Gabapentin-D4	0.13-1.27 / 0.7-7.4
Lamotrigine	84057-84-1	C9H7N5Cl2	12.6	$[M+H]^+$	Lamotrigin-13C3,d3	0.12-1.18 / 0.5-4.6
Molinate	2212-67-1	C9H17NOS	19.8	$[M+H]^+$	Atorvastatin-d5*	0.11-1.12 / 0.6-6.0
Paracetamol	103-90-2	C8H9NO2	10.1	$[M+H]^+$	Paracetamol-d4	0.63-6.30 / 4.2-41.7
Sucralose	56038-13-2	C12H19Cl3O8	13.0	$[M-H]^-$	sucralose-D6	0.62-6.16 / 1.6-15.5
Tramadol	27203-92-5	C16H25N1O2	12.0	$[M+H]^+$	Tramadol-D6	0.06-0.63 / 0.2-2.4
Triclosan	3380-34-5	C12H7Cl3O2	21.4	$[M-H]^-$	Valsartan-13C5,15N*	0.25-2.46 / 0.9-8.5

Table S2 continued.

Compound Name	CAS #	Molecular Formula	Retention time (min)	Ion precursors monitored	Internal standard used	Concentration range used (µg L^{-1}) / (nM)
Valsartan	137862-53-4	C24H29N5O3	19.2	$[M+H]^+$/$[M-H]^-$	Valsartan-13C5,15N	0.22-2.23 / 0.5-5.1
Valsartan acid	164265-78-5	C14H10N4O2	16.1	$[M+H]^+$/$[M-H]^-$	Valsartan acid-D4	0.22-2.21 / 0.8-8.3

*For micropollutants without a corresponding isotopically labelled standard, another internal standard was selected based on the best match in signal variation across multiple control samples.

Table S3. List of cyano-metabolites identified in *Planktothrix* and *Microcystis* extracts. Given concentrations are for experiments for which the extracted equivalent of 12.3 $mg_{biomass}$ L^{-1} of *Planktothrix rubescens* and 6.9 $mg_{biomass}$ L^{-1} of *Microcystis aeruginosa* were spiked (19.2 $mg_{biomass\text{-}equivalent}$ L^{-1} total). Lower cyanobacteria biomass concentrations were also used in some experiments (see Table S4). When no bioreagent was available, the bioreagent of a structurally similar compound was used.

Compound Name (*Cyanobacterial strain*)	CyanometDB ID # [28]	Molecular Formula	Retention time (min)	Ion precursors monitored	Internal standard used	Concentration* (µg L^{-1}) / (nM)
**Anabaenopeptin**						
Anabaenopeptin A *(Planktothrix)*	760	$C_{44}H_{57}N_7O_{10}$	17.5	$[M+H]^+$, $[M-H]^-$	Anabaenopeptin A-N15	34 / 40
Anabaenopeptin B *(Planktothrix)*	867	$C_{41}H_{60}N_{10}O_9$	14.5	$[M+H]^+$, $[M+2H]^{2+}$	Anabaenopeptin B-N15	4.8 / 5.7

Table S3 continued.

Compound Name (*Cyanobacterial strain*)	CyanometDB ID # [28]	Molecular Formula	Retention time (min)	Ion precursors monitored	Internal standard used	Concentration* (µg L^{-1}) / (nM)
Anabaenopeptin F *(Planktothrix)*	805	$C_{42}H_{62}N_{10}O_9$	15.0	$[M+H]^+$, $[M+2H]^{2+}$	Anabaenopeptin B-N15***	0.07 / 0.08**
**Cyanopeptolin**						
Cyanopeptolin 963A *(Microcystis)*	555	$C_{49}H_{69}N_7O_{13}$	20.1	$[M+H]^+$, $[M-H]^-$	Anabaenopeptin A-N15***	3.8 / 3.9**
Cyanopeptolin A *(Microcystis)*	640	$C_{46}H_{72}N_{10}O_{12}$	17.3	$[M+H]^+$, $[M-H]^-$	Anabaenopeptin A-N15***	23 / 24
Cyanopeptolin B *(Microcystis)*	642	$C_{46}H_{72}N_8O_{12}$	17.3	$[M+H]^+$, $[M-H]^-$	Anabaenopeptin A-N15***	6.3 / 6.8**
Cyanopeptolin C *(Microcystis)*	603	$C_{47}H_{74}N_8O_{12}$	17.3	$[M+H]^+$, $[M-H]^-$	Anabaenopeptin A-N15***	12 / 13**
Cyanopeptolin D *(Microcystis)*	571	$C_{48}H_{76}N_8O_{12}$	17.3	$[M+H]^+$, $[M-H]^-$	Anabaenopeptin A-N15***	25 / 26
**Cyclamide**						
Aerucyclamide A *(Microcystis)*	1466	$C_{24}H_{34}N_6O_4S_2$	20.4	$[M+H]^+$	Diazepam-D5***	80 / 150
Aerucyclamide B *(Microcystis)*	1472	$C_{24}H_{32}N_6O_4S_2$	20.9	$[M+H]^+$	Diazepam-D5***	3.6 / 6.7**
Aerucyclamide C *(Microcystis)*	1469	$C_{24}H_{32}N_6O_5S$	20.8	$[M+H]^+$	Diazepam-D5***	22 / 43**
Aerucyclamide D *(Microcystis)*	1402	$C_{26}H_{30}N_6O_4S_3$	19.8	$[M+H]^+$	Diazepam-D5***	8.1 / 15**
Microcyclamide 7806A *(Microcystis)*	1471	$C_{24}H_{34}N_6O_6S$	15.7	$[M+H]^+$, $[M-H]^-$	Clozapine-D8***	0.6 / 1.0**

Table S3 continued.

Compound Name (*Cyanobacterial strain*)	CyanometDB ID # [28]	Molecular Formula	Retention time (min)	Ion precursors monitored	Internal standard used	Concentration* (µg L^{-1}) / (nM)
Microcyclamide 7806B *(Microcystis)*	1465	$C_{24}H_{34}N_6O_6S$	18.4	$[M+H]^+$, $[M-H]^-$	Valsartan-13C5,15N***	2.6 / 4.9**
**Microcystin**						
$[D\text{-}Asp^3,(E)\text{-}Dhb^7]$MC-RR *(Planktothrix)*	1962	$C_{48}H_{73}N_{13}O_{12}$	15.2	$[M+2H]^{2+}$, $[M-H]^-$, $[M-2H]^{2-}$	$[D\text{-}Asp^3,(E)\text{-}Dhb^7]$MC-RR-N15	294 / 287
$[D\text{-}Asp^3,Dha^7]$MC-RR *(Planktothrix)*	1884	$C_{47}H_{71}N_{13}O_{12}$	15.1	$[M+H]^+$, $[M+2H]^{2+}$, $[M-H]^-$	$[D\text{-}Asp^3,(E)\text{-}Dhb^7]$MC-RR-N15**	3.1 / 3.1**
$[D\text{-}Asp^3]$MC-LR *(Microcystis and Planktothrix)*	1953	$C_{48}H_{72}N_{10}O_{12}$	17.3	$[M+H]^+$, $[M+2H]^{2+}$, $[M-H]^-$	Bezafibrate-D4***	5.8 / 5.9
MC-LR *(Microcystis and Planktothrix)*	1823	$C_{49}H_{74}N_{10}O_{12}$	17.3	$[M+H]^+$, $[M+2H]^{2+}$, $[M-H]^-$	Bezafibrate-D4***	12 / 12
**Other**						
Planktocyclin *(Planktothrix)*	939	$C_{39}H_{60}N_8O_8S$	19.9	$[M+H]^+$, $[M-H]^-$	Atorvastatin-d5***	N/A

* The concentration is given for 19.2 $mg_{biomass\text{-}equivalent}$ L^{-1}. Individual cyano-metabolite concentrations are then proportional to the biomass concentration spiked in other experiments.

** Quantified using a bioreagent of a structurally similar cyano-metabolite

*** For micropollutants without a corresponding isotopically labelled standard, an internal standard was selected based on the best match in signal variation across multiple control samples.

Table S4. Experimental conditions for the sand column experiments. Each experiment was labelled by a letter (A-M), associated with a number if repeated in the same column (e.g., C1, C2…). Individual micropollutant and cyano-metabolite concentrations are given in Table S2 and S3. The timeline of the experiments is shown in Table S5.

Experiment	Temperature (°C)	Cyano-metabolite concentration* (as total $mg_{biomass-equivalent}$ L^{-1})	Micropollutant concentration** range (µg L^{-1})	Flow rate (mL min^{-1})
**Column #1**				
A	21	19.2	0.6-6.3	0.25
C	21	19.2	0.6-6.3	1
D	21	19.2	0.6-6.3	2
E	21	19.2	0.6-6.3	4
F	11	19.2	0.6-6.3	1
G	4	19.2	0.6-6.3	1
H	21	192	0.6-6.3	0.25
**Column #2**				
B	21	19.2	0.6-6.3	0.5
C1, C2, C3	21	19.2	0.6-6.3	1
D	21	19.2	0.6-6.3	2
E1, E2	21	19.2	0.6-6.3	4
F	11	19.2	0.6-6.3	1
G	4	19.2	0.6-6.3	1
I	21	9.6	0.5-3.1	1
J1, J2	21	3.8	0.2-1.2	1
K	21	1.9	0.1-0.6	1
L	21	3.8	0.6-6.3	1
M***	21	19.2	0.6-6.3	1

* Individual cyano-metabolite concentrations are given in Table S3

** Individual micropollutant concentrations are given in Table S2

*** Sand was autoclaved

Table S5. Timeline of experiments in columns #1 and #2. The experimental conditions corresponding to the experimental label are shown in Table S4.

<table>
<tr><th colspan="2">Column #1</th><th colspan="2">Column #2</th></tr>
<tr><th>Days</th><th>Experiment</th><th>Days</th><th>Experiment</th></tr>
<tr><td rowspan="6">1-6</td><td rowspan="6">Pre-tests at high cyanometabolite concentrations (equivalent to 192 $mg_{biomass}$ L^{-1}) and 0.6-6.3 µg L^{-1} micropollutants</td><td>1</td><td>B/E1</td></tr>
<tr><td>2</td><td>C1/D</td></tr>
<tr><td>3</td><td>I/J1</td></tr>
<tr><td>4</td><td>K</td></tr>
<tr><td>5</td><td>C2/E2</td></tr>
<tr><td>6</td><td>J2</td></tr>
<tr><td>7</td><td>H</td><td>7</td><td>L</td></tr>
<tr><td>8</td><td>A/C</td><td rowspan="5">8-24</td><td rowspan="5">Pause with feeding the column with cyanometabolite from 0.1$mg_{biomass}$ L^{-1} and 0.05-0.3 µg L^{-1} micropollutants</td></tr>
<tr><td>9</td><td>D/E</td></tr>
<tr><td>10</td><td>F</td></tr>
<tr><td>11</td><td>G</td></tr>
<tr><td></td><td></td></tr>
<tr><td></td><td></td><td>25</td><td>G</td></tr>
<tr><td></td><td></td><td>26</td><td>F</td></tr>
<tr><td></td><td></td><td>27</td><td>C3</td></tr>
<tr><td></td><td></td><td>28</td><td>Sand autoclaving</td></tr>
<tr><td></td><td></td><td>29</td><td>M</td></tr>
</table>

Table S6. Average relative abatement expressed in % for each experiment (labelled by letters) conducted in column #1. The uncertainty is the standard deviation of 3 to 6 sampling points. See Table S4 for the experimental condition of each experiment.

**Cyano-metabolites**	A	C	D	E	F	G	H
Anabaenopeptins							
Anabaenopeptin A	100 ± 0	100 ± 0	100 ± 0	93 ± 0	100 ± 0	100 ± 0	100 ± 0
Anabaenopeptin B	100 ± 0	100 ± 0	99 ± 0	90 ± 1	100 ± 0	100 ± 1	100 ± 0
Anabaenopeptin F	100 ± 0	100 ± 0	100 ± 0	94 ± 0	100 ± 0	100 ± 2	100 ± 0
Cyanopeptolins							
Cyanopeptolin 963A	100 ± 0	100 ± 0	98 ± 3	87 ± 7	98 ± 2	68 ± 7	96 ± 2
Cyanopeptolin A	99 ± 0	99 ± 0	98 ± 0	91 ± 1	98 ± 0	85 ± 2	98 ± 0
Cyanopeptolin B	100 ± 0	100 ± 0	98 ± 1	89 ± 1	99 ± 2	85 ± 1	98 ± 1
Cyanopeptolin C	99 ± 0	99 ± 0	98 ± 0	87 ± 2	98 ± 0	81 ± 1	96 ± 1
Cyanopeptolin D	97 ± 0	98 ± 0	97 ± 0	83 ± 1	97 ± 1	71 ± 4	90 ± 3
Cyclamides							
Aerucyclamide A	99 ± 0	83 ± 11	62 ± 3	41 ± 0	57 ± 1	38 ± 3	37 ± 10
Aerucyclamide B	96 ± 2	90 ± 4	79 ± 8	56 ± 9	76 ± 5	64 ± 5	48 ± 8
Aerucyclamide C	88 ± 1	81 ± 10	67 ± 3	49 ± 0	59 ± 1	52 ± 1	50 ± 4
Aerucyclamide D	100 ± 0	87 ± 6	65 ± 3	45 ± 1	64 ± 2	50 ± 2	50 ± 6
Microcyclamide 7806A	87 ± 2	80 ± 12	65 ± 3	43 ± 3	69 ± 2	58 ± 3	51 ± 4
Microcyclamide 7806B	93 ± 2	66 ± 18	48 ± 4	28 ± 4	37 ± 3	12 ± 5	16 ± 6
Microcystins							
[D-Asp^3,(E)-Dhb^7]MC-RR	100 ± 0	88 ± 8	68 ± 3	34 ± 2	54 ± 6	27 ± 6	43 ± 6
[D-Asp^3,Dha^7]MC-RR	100 ± 0	93 ± 7	76 ± 3	43 ± 1	68 ± 6	37 ± 5	61 ± 6
[D-Asp^3]MC-LR	100 ± 0	77 ± 16	58 ± 4	37 ± 1	44 ± 3	28 ± 3	32 ± 5
MC-LR	92 ± 3	64 ± 20	47 ± 3	31 ± 3	39 ± 3	22 ± 5	11 ± 10
Unclassified							
Planktocyclin	98 ± 0	96 ± 1	88 ± 1	67 ± 4	85 ± 1	90 ± 1	99 ± 0
**Micropollutants**							
Acesulfame	2 ± 3	7 ± 8	4 ± 4	9 ± 13	-6 ± 5	7 ± 3	4 ± 3
Atenolol	55 ± 4	23 ± 5	17 ± 5	3 ± 2	28 ± 3	12 ± 0	58 ± 6
Carbamazepine	6 ± 14	0 ± 21	-15 ± 4	-7 ± 1	2 ± 3	2 ± 5	19 ± 3
Diclofenac	9 ± 4	2 ± 4	1 ± 4	5 ± 8	0 ± 4	-2 ± 3	0 ± 7
Gabapentin	22 ± 5	6 ± 7	0 ± 3	2 ± 3	8 ± 4	5 ± 4	11 ± 6
Lamotrigine	0 ± 5	-1 ± 6	0 ± 5	2 ± 1	1 ± 3	-11 ± 5	1 ± 2
Molinate	23 ± 1	6 ± 3	-7 ± 4	-16 ± 1	-5 ± 3	3 ± 7	12 ± 3
Paracetamol	60 ± 5	32 ± 6	18 ± 5	2 ± 5	24 ± 4	12 ± 3	52 ± 3
Sucralose	0 ± 8	-2 ± 4	-1 ± 4	-2 ± 4	1 ± 4	2 ± 4	1 ± 3
Tramadol	-1 ± 5	0 ± 6	-4 ± 6	-1 ± 3	-11 ± 5	-2 ± 3	9 ± 8
Triclosan	95 ± 2	74 ± 8	64 ± 7	33 ± 2	77 ± 12	78 ± 1	98 ± 1
Valsartan	24 ± 3	15 ± 7	14 ± 5	10 ± 3	12 ± 5	7 ± 3	14 ± 6
Valsartan acid	0 ± 11	2 ± 10	-7 ± 5	-4 ± 7	0 ± 5	1 ± 3	-3 ± 5

Table S7. Average relative abatement expressed in % for each experiment (labelled by letters) conducted in column #2. The uncertainty is the standard deviation of 3 to 6 sampling points. See Table S4 for the experimental condition of each experiment. Statistical significance between given experiments was assessed using a two-sample Welch's *t*-test with $\alpha = 0.05$.[29]

**Cyano-metabolites**	B	C1	C2	C3	D	E1	E2	J1	J2	F	G	I	K	L	M
Anabaenopeptins															
Anabaenopeptin A	100 ± 0	98 ± 1[a]	99 ± 0[d]	99 ± 1	91 ± 1[b]	53 ± 2[c]	80 ± 2[f]	100 ± 0	100 ± 0	54 ± 2	19 ± 3	100 ± 0	100 ± 0	100 ± 0	6 ± 3[h]
Anabaenopeptin B	97 ± 1	88 ± 1[a]	97 ± 1[d]	95 ± 1[d]	70 ± 1[b]	32 ± 3[c]	62 ± 4[f]	100 ± 0	100 ± 0	49 ± 2	24 ± 4	99 ± 0[d,e]	100 ± 0	100 ± 0	7 ± 3[h]
Anabaenopeptin F	99 ± 1	89 ± 4[a]	99 ± 3[d]	96 ± 3[d]	65 ± 5[b]	30 ± 7[c]	76 ± 10[f]	N.D.	N.D.	47 ± 4	23 ± 5	93 ± 0	N.D.	N.D.	9 ± 6[h]
Cyanopeptolins													N.D.	N.D.	
Cyanopeptolin 963A	79 ± 4	51 ± 10[a]	70 ± 8[d]	78 ± 5[d]	27 ± 13[b]	15 ± 8[c]	30 ± 17	N.D.	N.D.	24 ± 3	11 ± 9	83 ± 8[d]	N.D.	N.D.	47 ± 17[h]
Cyanopeptolin A	89 ± 1	77 ± 7[a]	94 ± 2[d]	94 ± 1[d]	60 ± 11[b]	32 ± 8[c]	68 ± 6[f]	97 ± 1[i]	100 ± 0[g,i]	59 ± 5	47 ± 6	93 ± 0[d]	100 ± 0[g]	N.D.	61 ± 3[h]
Cyanopeptolin B	81 ± 1	64 ± 10[a]	96 ± 4[d]	92 ± 3[d]	45 ± 9[b]	17 ± 7[c]	66 ± 8[f]	N.D.	N.D.	43 ± 4	27 ± 6	96 ± 2[d]	N.D.	N.D.	61 ± 12[h]
Cyanopeptolin C	65 ± 3	48 ± 13[a]	92 ± 1[d]	90 ± 4[d]	35 ± 11[b]	10 ± 10[c]	57 ± 7[f]	N.D.	N.D.	33 ± 5	21 ± 6	94 ± 1[d]	N.D.	N.D.	39 ± 4[h]
Cyanopeptolin D	40 ± 6	28 ± 18[a]	87 ± 3[d]	86 ± 6[d]	19 ± 13[b]	3 ± 10[c]	45 ± 7[f]	97 ± 1[i]	100 ± 0[g,i]	24 ± 9	15 ± 8	92 ± 1[d,e]	100 ± 0[g]	N.D.	52 ± 11[h]
Cyclamides															
Aerucyclamide A	6 ± 4	-11 ± 6[a]	14 ± 4[d]	41 ± 6[d,e]	-10 ± 4[b]	0 ± 6[c]	5 ± 3	22 ± 6	54 ± 0[g,i]	18 ± 3	8 ± 6	30 ± 1[d,e]	36 ± 5[g]	51 ± 0	2 ± 8[h]
Aerucyclamide B	11 ± 8	0 ± 0[a]	28 ± 1[d]	47 ± 6[d,e]	-13 ± 0[b]	1 ± 3[c]	N.D.	20 ± 11	N.D.	27 ± 4	16 ± 10	34 ± 1[d,e]	N.D.	N.D.	17 ± 12[h]
Aerucyclamide C	8 ± 7	0 ± 0[a]	22 ± 4[d]	48 ± 5[d,e]	-7 ± 0[b]	-2 ± 4[c]	N.D.	24 ± 8	50 ± 0[g,i]	24 ± 4	22 ± 6	31 ± 3[d,e]	33 ± 11[g]	60 ± 0	19 ± 11[h]
Aerucyclamide D	26 ± 5	7 ± 6[a]	21 ± 4[d]	44 ± 7[d,e]	-1 ± 3[b]	8 ± 2[c]	5 ± 5	41 ± 9	65 ± 0[g,i]	25 ± 2	14 ± 6	39 ± 1[d,e]	N.D.	76 ± 0	100 ± 0
Microcyclamide 7806A	21 ± 8	14 ± 0[a]	25 ± 28	53 ± 9[d]	8 ± 0[b]	-4 ± 5[c]	-4 ± 15	39 ± 30	N.D.	26 ± 6	27 ± 10	17 ± 10	N.D.	N.D.	-14 ± 10[h]
Microcyclamide 7806B	2 ± 9	-7 ± 9[a]	9 ± 5[d]	29 ± 6[d,e]	-4 ± 7[b]	-1 ± 9[c]	0 ± 9	17 ± 6	46 ± 4[g,i]	16 ± 7	5 ± 8	24 ± 3[d,e]	N.D.	46 ± 4	0 ± 8[h]
Unclassified															
Planktocyclin	97 ± 0	75 ± 13[a]	95 ± 1	96 ± 2[d]	51 ± 16[b]	30 ± 12[c]	58 ± 5[f]	99 ± 1	100 ± 0i	77 ± 8	70 ± 10	98 ± 1[d,e]	100 ± 0	100 ± 0	100 ± 0

Table S7 continued.

Cyano-metabolites	B	C1	C2	C3	D	E1	E2	J1	J2	F	G	I	K	L	M
Microcystins															
[D-Asp^{3},(E)-Dhb^{7}]MC-RR	60 ± 5	35 ± 4[a]	65 ± 2[d]	76 ± 2[d,e]	26 ± 7[b]	9 ± 6[c]	24 ± 3[f]	97 ± 1[i]	100 ± 0[g,i]	33 ± 2	1 ± 16	85 ± 2[d,e]	100 ± 0	100 ± 0	15 ± 7[h]
[D-Asp^{3},Dha^{7}]MC-RR	83 ± 2	56 ± 3[a]	84 ± 3[d]	87 ± 2[d]	41 ± 3[b]	18 ± 5[c]	34 ± 1[f]	N.D.	N.D.	32 ± 9	44 ± 13	97 ± 1[d,e]	N.D.	N.D.	-20 ± 9[h]
[D-Asp^{3}]MC-LR	55 ± 3	31 ± 3[a]	63 ± 3[d]	69 ± 2[d,e]	8 ± 6[b]	2 ± 5[c]	18 ± 4[f]	92 ± 1[i]	N.D.	26 ± 3	12 ± 8	80 ± 1[d,e]	N.D.	N.D.	15 ± 8[h]
MC-LR	11 ± 5	7 ± 4[a]	39 ± 5[d]	48 ± 4[d,e]	-3 ± 5[b]	-3 ± 3[c]	12 ± 4[f]	61 ± 3[i]	95 ± 2[g,i]	19 ± 3	9 ± 7	53 ± 2[d,e]	94 ± 6[g]	95 ± 2	8 ± 4[h]
**Micropollutants**															
Acesulfame	-1 ± 8	7 ± 10	2 ± 6	-2 ± 3	-13 ± 17	-3 ± 8	-2 ± 1	-2 ± 4	5 ± 10	-5 ± 4	-2 ± 2	6 ± 3	-8 ± 5	-4 ± 1	-1 ± 3
Atenolol	53 ± 2	36 ± 8	25 ± 3	45 ± 4[e]	12 ± 4	16 ± 4	19 ± 0	40 ± 3	34 ± 6	19 ± 2	6 ± 7	37 ± 1[e]	44 ± 4	32 ± 6	26 ± 12
Carbamazepine	0 ± 6	-1 ± 3	2 ± 4	4 ± 6	-7 ± 2	-5 ± 9	-15 ± 0	-7 ± 3	-4 ± 7	7 ± 3	-1 ± 6	0 ± 2	1 ± 7	-13 ± 11	6 ± 5
Diclofenac	-1 ± 4	1 ± 5	2 ± 7	2 ± 11	-10 ± 5	2 ± 4	-9 ± 12	-15 ± 17	-2 ± 6	6 ± 2	27 ± 7	-10 ± 15	-26 ± 13	3 ± 3	1 ± 4
Gabapentin	18 ± 2	3 ± 10	-5 ± 4	6 ± 5[e]	-5 ± 5	8 ± 5	6 ± 0	9 ± 4[i]	12 ± 4i	-4 ± 4	2 ± 6	0 ± 2	17 ± 4[g]	13 ± 6	18 ± 3
Lamotrigine	9 ± 4	0 ± 3	5 ± 3	4 ± 6	-8 ± 5	-2 ± 1[c]	-10 ± 0	-6 ± 3	2 ± 1[g,i]	-6 ± 6	7 ± 1	-5 ± 2	-7 ± 4	-1 ± 6	6 ± 1
Molinate	24 ± 5	3 ± 9	10 ± 5	8 ± 3	0 ± 3	-4 ± 9	2 ± 0	21 ± 3[i]	26 ± 7i	4 ± 12	4 ± 9	12 ± 4	43 ± 17	5 ± 7	7 ± 6
Paracetamol	34 ± 3	14 ± 8[a]	8 ± 2	11 ± 5	6 ± 3[b]	10 ± 3	10 ± 0	23 ± 3[i]	35 ± 4[g,i]	2 ± 5	10 ± 7	18 ± 1[e]	25 ± 5	13 ± 4	0 ± 3[h]
Sucralose	6 ± 4	11 ± 6[a]	3 ± 4	-8 ± 2	-6 ± 4	0 ± 4	-9 ± 0	5 ± 4	-4 ± 9	1 ± 9	-9 ± 6	5 ± 2	3 ± 7	-4 ± 4	-14 ± 7
Tramadol	7 ± 3	-2 ± 3	0 ± 3	-4 ± 1	-7 ± 10	-4 ± 3	-2 ± 1	-4 ± 7	2 ± 2	4 ± 3	2 ± 3	5 ± 3[d]	-6 ± 7	6 ± 5	4 ± 1
Triclosan	100 ± 0	78 ± 7	93 ± 5[d]	71 ± 16	6 ± 32[b]	18 ± 17	9 ± 1	-5 ± 10	78 ± 7[g,i]	78 ± 5	93 ± 1	43 ± 9	58 ± 20[g]	83 ± 7	98 ± 1
Valsartan	40 ± 2	15 ± 3	23 ± 4[d]	23 ± 6	5 ± 2[b]	4 ± 4[c]	5 ± 8	21 ± 7	28 ± 7	5 ± 4	1 ± 7	22 ± 4[d]	24 ± 7	20 ± 3	6 ± 6[h]
Valsartan acid	-6 ± 5	-8 ± 6	-9 ± 6	-8 ± 5	-7 ± 9	-2 ± 4	-3 ± 9	-7 ± 6	-3 ± 7	6 ± 4	0 ± 10	-5 ± 13	-18 ± 12	3 ± 11	-1 ± 6

[a] Values are significantly lower than in experiment C of column #1 (Table S6). [b] Values are significantly lower than in experiment D of column #1 (Table S6). [c] Values are significantly lower than in experiment E of column #1 (Table S6). [d] Values are significantly higher than in experiment C1. [e] Values are significantly higher than in experiment C2.[f] Values are significantly higher than in experiment E1. [g] Values are significantly higher than in experiment J1. [h] Values are significantly lower than in experiment C3. [i] Values are significantly higher than in experiment I.

Table S8. Apparent first-order rate constants k_{app} for the abatement of cyano-metabolites and micropollutants in the sand columns. The determination of k is explained in Text S4.1. Experiments labelled A-D and B/C1/D/E1 were used for the regressions for column #1 and column #2, respectively. Only values from correlations with a $R^2 \geq 0.8$ are reported. Uncertainty corresponds to the 95-confidence interval of the slope. N.D stands for Not Determined and are for correlations with R^2 <0.8.

	**Column #1**			**Column #2**		
**Cyano-metabolites**	Apparent k_{app} (s^{-1})	n	R^2	Apparent k_{app} (s^{-1})	n	R^2
Anabaenopeptins						
Anabaenopeptin A	$(2.0 \pm 0.2) \times 10^{-2}$	8	0.99	$(5.0 \pm 0.5) \times 10^{-3}$	26	0.95
Anabaenopeptin B	$(1.7 \pm 0.2) \times 10^{-2}$	8	0.99	$(2.7 \pm 0.2) \times 10^{-3}$	27	0.96
Anabaenopeptin F	N.D.			$(3.7 \pm 0.6) \times 10^{-3}$	21	0.96
Cyanopeptolins						
Cyanopeptolin 963A	$(9.2 \pm 1.8) \times 10^{-3}$	8	0.96	$(1.3 \pm 0.2) \times 10^{-3}$	27	0.95
Cyanopeptolin A	$(8.3 \pm 1.7) \times 10^{-3}$	8	0.96	$(1.6 \pm 0.2) \times 10^{-3}$	27	0.90
Cyanopeptolin B	$(1.1 \pm 0.1) \times 10^{-2}$	8	0.99	$(1.2 \pm 0.2) \times 10^{-3}$	17	0.92
Cyanopeptolin C	$(1.0 \pm 0.2) \times 10^{-2}$	8	0.96	$(7.9 \pm 1.3) \times 10^{-4}$	27	0.86
Cyanopeptolin D	$(9.6 \pm 1.5) \times 10^{-3}$	8	0.82	N.D.		
Cyclamides						
Aerucyclamide A	$(1.8 \pm 0.1) \times 10^{-3}$	14	0.99	N.D.		
Aerucyclamide B	N.D.			N.D.		
Aerucyclamide C	$(5.2 \pm 1.3) \times 10^{-4}$	14	0.87	N.D.		
Aerucyclamide D	$(2.2 \pm 0.2) \times 10^{-3}$	12	0.97	N.D.		
Microcyclamide 7806A	$(5.0 \pm 1.5) \times 10^{-4}$	14	0.82	N.D.		
Microcyclamide 7806B	$(7.2 \pm 0.8 \times 10^{-4}$	14	0.97	N.D.		
Microcystins						
[D-Asp^3,(E)-Dhb^7]MC-RR	$(2.6 \pm 0.2) \times 10^{-3}$	14	0.99	$(7.0 \pm 0.7) \times 10^{-4}$	27	0.95
[D-Asp^3,Dha^7]MC-RR	$(3.0 \pm 0.7) \times 10^{-3}$	12	0.91	$(1.4 \pm 0.1) \times 10^{-3}$	27	0.99
[D-Asp^3]MC-LR	$(2.2 \pm 0.3) \times 10^{-3}$	14	0.96	$(7.6 \pm 0.6) \times 10^{-4}$	27	0.97
MC-LR	$(9.4 \pm 1.2) \times 10^{-4}$	14	0.96	N.D.		
Unclassified						
Planktocyclin	N.D.			$(2.5 \pm 0.4) \times 10^{-3}$	27	0.89
**Micropollutants**						
Acesulfame	N.D.			N.D.		
Atenolol	$(2.8 \pm 0.5) \times 10^{-4}$	14	0.92	$(5.6 \pm 0.7) \times 10^{-4}$	27	0.91
Carbamazepine	N.D.			N.D.		

Table S8 continued.

	Column #1			Column #2		
**Micropollutants**	Apparent k_{app} (s^{-1})	n	R^2	Apparent k_{app} (s^{-1})	n	R^2
Diclofenac	N.D.			N.D.		
Gabapentin	$(5.4 \pm 2.5) \times 10^{-5}$	6	0.90	N.D.		
Lamotrigine	N.D.			N.D.		
Molinate	$(9.1 \pm 3.3) \times 10^{-5}$	6	0.94	$(2.4 \pm 0.6) \times 10^{-4}$	13	0.88
Paracetamol	$(3.6 \pm 0.5) \times 10^{-4}$	14	0.95	$(3.1 \pm 1.0) \times 10^{-4}$	7	0.87
Sucralose	N.D.			N.D.		
Tramadol	N.D.			N.D.		
Triclosan	N.D.			N.D.		
Valsartan	N.D.			$(4.5 \pm 0.6) \times 10^{-4}$	27	0.92
Valsartan acid	N.D.			N.D.		

Table S9. Activation energies E_a for the abatement of cyano-metabolites and micropollutants in the sand columns. The determination of E_a is explained in Text S4.1. Experiments labelled C/F/G and C3/F/G were used for the regressions for column #1 and column #2, respectively. Only values from correlations with a $R^2 \geq 0.8$ are reported. Uncertainty corresponds to the 95-confidence interval of the slope. N.D stands for Not Determined and are for correlations with $R^2 < 0.8$. The interpretation of the E_a values is given in Text S4.5.

	Column #1			Column #2		
**Cyano-metabolites**	E_a (kJ mol^{-1})	n	R^2	E_a (kJ mol^{-1})	n	R^2
Anabaenopeptins						
Anabaenopeptin A	N.D.			121 ± 8	13	0.99
Anabaenopeptin B	N.D.			97 ± 10	13	0.98
Anabaenopeptin F	N.D.			102 ± 12	13	0.97
Cyanopeptolins						
Cyanopeptolin 963A	N.D.			107 ± 33	13	0.84
Cyanopeptolin A	N.D.			61 ± 14	13	0.90
Cyanopeptolin B	N.D.			87 ± 17	13	0.91
Cyanopeptolin C	N.D.			95 ± 24	13	0.90
Cyanopeptolin D	N.D.			105 ± 24	13	0.90
Cyclamides						
Aerucyclamide A	40 ± 6	10	0.97	62 ± 14	12	0.92
Aerucyclamide B	31 ± 11	10	0.84	N.D.		

Table S9 continued.

	Column #1			Column #2		
**Cyano-metabolite**	E_a (kJ mol^{-1})	n	R^2	E_a (kJ mol^{-1})	n	R^2
Aerucyclamide C	25 ± 4	10	0.96	N.D.		
Aerucyclamide D	37 ± 4	10	0.98	N.D.		
Microcyclamide 7806A	N.D.			N.D.		
Microcyclamide 7806B	56 ± 16	9	0.91	N.D.		
Microcystins						
[D-Asp^3,(E)-Dhb^7]MC-RR	67 ± 11	10	0.97	97 ± 25	11	0.89
[D-Asp^3,Dha^7]MC-RR	60 ± 9	10	0.97	N.D.		
[D-Asp^3]MC-LR	42 ± 11	10	0.94	107 ± 31	13	0.84
MC-LR	N.D.			73 ± 14	12	0.93
Unclassified	N.D.					
Planktocyclin	N.D.			N.D.		
**Micropollutants**	N.D.					
Acesulfame	N.D.					
Atenolol	N.D.			83 ± 23	12	0.86
Carbamazepine	N.D.					
Diclofenac	N.D.					
Gabapentin	N.D.					
Lamotrigine	N.D.					
Molinate	N.D.					
Paracetamol	39 ± 15	10	0.81			
Sucralose	N.D.					
Tramadol	N.D.					
Triclosan	N.D.					
Valsartan	N.D.			N.D.		
Valsartan acid	N.D.					

Table S10a. Comparison of R^2 and *p*-value for first- and zero-order kinetic fits. R^2 is the correlation coefficient for a linear regression of determination of C/C_0 vs time (for zero-order) or $\ln(C/C_0)$ vs time (for first-order). The difference between the zero- and first-order R^2 indicates the better fit. *p*-values are from the two-sided t-test (Table S10b) under the null hypothesis "intercept is significantly different from 1 (for zero-order) or 0 (for first-order)". A high *p*-value means that the hypothesis is more likely rejected, hence the intercept more likely not statistically different from the expected intercept. The ratio between the zero- and first-order *p*-values indicates which is closer to the expected intercept. A ratio << 1 means that first-order gives a better intercept while a ratio >> 1 indicates that zero-order gives a better intercept. N.D stands for Not Determined for correlations with $R^2 < 0.8$.

	$R^2_{zero\text{-}order} - R^2_{first\text{-}order}$		$p\text{-value}_{zero\text{-}order}$ / $p\text{-value}_{first\text{-}order}$	
**Cyano-metabolites**	Column #1	Column #2	Column #1	Column #2
Anabaenopeptins				
Anabaenopeptin A	<0.01	-0.45	6E-012	3E-10
Anabaenopeptin B	<0.01	-0.27	1E-09	4E-07
Anabaenopeptin F	N.D.	-0.03	N.D.	5E-03
Cyanopeptolins				
Cyanopeptolin 963A	<0.01	<0.01	6E-08	1E+03
Cyanopeptolin A	-0.03	-0.10	4E-07	8E-07
Cyanopeptolin B	<0.01	-0.08	2E-010	5E-04
Cyanopeptolin C	<0.01	-0.05	2E-08	3E-02
Cyanopeptolin D	<0.01	N.D.	1E-09	N.D.
Cyclamides				
Aerucyclamide A	-0.23	N.D.	9E-05	N.D.
Aerucyclamide B	N.D.	N.D.	N.D.	N.D.
Aerucyclamide C	-0.21	N.D.	5E-03	N.D.
Aerucyclamide D	-0.05	N.D.	1E-04	N.D.
Microcyclamide 7806A	-0.22	N.D.	6E-03	N.D.
Microcyclamide 7806B	-0.11	N.D.	5E-03	N.D.
Microcystins				
[D-Asp^3,(E)-Dhb^7]MC-RR	-0.12	-0.04	4E-03	1E+00
[D-Asp^3,Dha^7]MC-RR	-0.16	-0.07	1E-03	1E-06
[D-Asp^3]MC-LR	-0.09	-0.02	3E-09	5E+02
MC-LR	-0.05	N.D.	2E-07	N.D.
Unclassified				
Planktocyclin	N.D.	-0.02	N.D.	1E-05

Table S10a continued.

	$R^2_{zero\text{-}order} - R^2_{first\text{-}order}$		$p\text{-value}_{zero\text{-}order} / p\text{-value}_{first\text{-}order}$	
**Micropollutants**	Column #1	Column #2	Column #1	Column #2
Acesulfame	N.D.	N.D.	N.D.	N.D.
Atenolol	-0.07	-0.02	2E-01	6E-03
Carbamazepine	N.D.	N.D.	N.D.	N.D.
Diclofenac	N.D.	N.D.	N.D.	N.D.
Gabapentin	<0.01	N.D.	5E-01	N.D.
Lamotrigine	N.D.	N.D.	N.D.	N.D.
Molinate	-0.01	<0.01	7E-01	2E+00
Paracetamol	-0.09	N.D.	1E-01	N.D.
Sucralose	N.D.	N.D.	N.D.	N.D.
Tramadol	N.D.	N.D.	N.D.	N.D.
Triclosan	N.D.	N.D.	N.D.	N.D.
Valsartan	N.D.	<0.01	N.D.	5E+00
Valsartan acid	N.D.	N.D.	N.D.	N.D.

Table S10b. Result of two-sided *t*-tests assessing the null hypothesis "intercept is significantly different from 1 (for zero-order) or 0 (for first-order)", with a significance level $\alpha = 0.05$. Details are provided in Text S4.3. Experiments labelled A-D and B/C1/D/E1 were used for the statistical test for column #1 and column #2, respectively. N.D stands for Not Determined for correlations with $R^2 < 0.8$.

	**Column #1**		**Column #2**	
**Cyano-metabolites**	Zero-order *t*-test	First-order *t*-test	Zero-order *t*-test	First-order *t*-test
Anabaenopeptins				
Anabaenopeptin A	TRUE	TRUE	TRUE	TRUE
Anabaenopeptin B	TRUE	TRUE	TRUE	TRUE
Anabaenopeptin F	N.D.	N.D.	TRUE	FALSE
Cyanopeptolins				
Cyanopeptolin 963A	TRUE	FALSE	TRUE	TRUE
Cyanopeptolin A	TRUE	TRUE	TRUE	TRUE
Cyanopeptolin B	TRUE	FALSE	TRUE	FALSE
Cyanopeptolin C	TRUE	FALSE	TRUE	FALSE
Cyanopeptolin D	TRUE	FALSE	N.D.	N.D.
Cyclamides				
Aerucyclamide A	TRUE	TRUE	N.D.	N.D.
Aerucyclamide B	N.D.	N.D.	N.D.	N.D.
Aerucyclamide C	TRUE	TRUE	N.D.	N.D.

Table S10b continued.

	Column #1		Column #2	
**Cyano-metabolites**	Zero-order *t*-test	First-order *t*-test	Zero-order *t*-test	First-order *t*-test
Aerucyclamide D	TRUE	TRUE	N.D.	N.D.
Microcyclamide 7806A	TRUE	TRUE	N.D.	N.D.
Microcyclamide 7806B	TRUE	TRUE	N.D.	N.D.
Microcystins				
[D-Asp^3,(E)-Dhb^7]MC-RR	TRUE	FALSE	FALSE	FALSE
[D-Asp^3,Dha^7]MC-RR	TRUE	FALSE	TRUE	FALSE
[D-Asp^3]MC-LR	TRUE	FALSE	TRUE	TRUE
MC-LR	TRUE	TRUE	N.D.	N.D.
Unclassified				
Planktocyclin	N.D.	N.D.	TRUE	FALSE
**Micropollutants**				
Acesulfame	N.D.	N.D.	N.D.	N.D.
Atenolol	TRUE	TRUE	TRUE	FALSE
Carbamazepine	N.D.	N.D.	N.D.	N.D.
Diclofenac	N.D.	N.D.	N.D.	N.D.
Gabapentin	TRUE	TRUE	N.D.	N.D.
Lamotrigine	N.D.	N.D.	N.D.	N.D.
Molinate	FALSE	FALSE	FALSE	TRUE
Paracetamol	TRUE	TRUE	N.D.	N.D.
Sucralose	N.D.	N.D.	N.D.	N.D.
Tramadol	N.D.	N.D.	N.D.	N.D.
Triclosan	N.D.	N.D.	N.D.	N.D.
Valsartan	N.D.	N.D.	TRUE	TRUE
Valsartan acid	N.D.	N.D.	N.D.	N.D.

Table S11. Identified products formed in the sand column. MS^2 fragmentation was used to validate the structures (see SI2).

Name	Precursor(s)	Formula / *Ion detected**	Structure	RT (min)	Confidence level[23]	Reference
Aerucyclamide D-sulfoxide**	Aerucyclamide D	$C_{26}H_{30}N_6O_5S_3$ *$[M+H]^+$* *$[M-H]^-$*	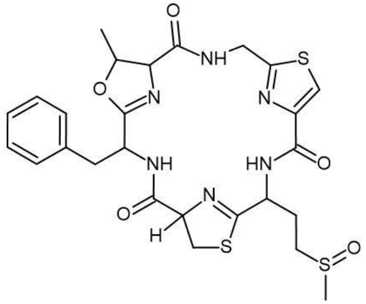	17.5	2b	This study
Ana-TP679	Anabaenopeptin A & B	$C_{35}H_{49}N_7O_7$ *$[M+H]^+$* *$[M-H]^-$*	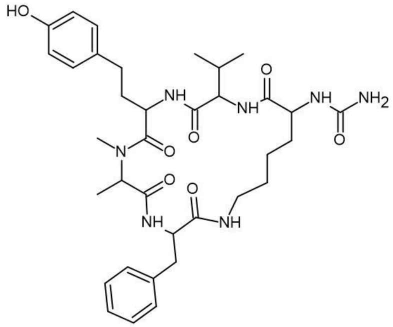	17.1	2b	[21]
Ana-TP636	Anabaenopeptin A & B	$C_{34}H_{48}N_6O_6$ *$[M+H]^+$* *$[M-H]^-$*	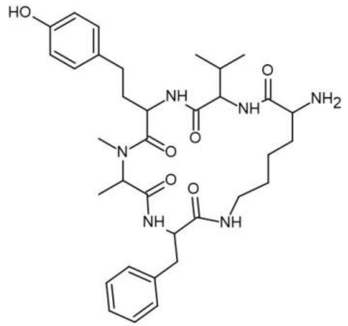	14.6	2b	[21]

Table S11 continued.

Name	Precursor(s)	Formula / *Ion detected**	Structure	RT (min)	Confidence level[23]	Reference
Ana-TP693***	Oscillamide Y	$C_{36}H_{51}N_7O_7$ *$[M+H]^+$*	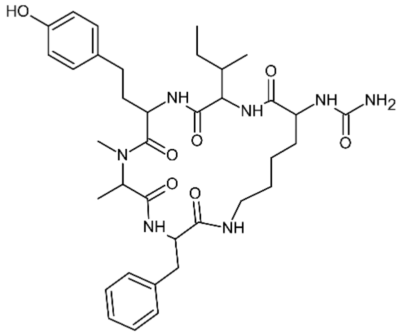	n.a.	5	21
Ana-TP650***	Oscillamide Y	$C_{35}H_{50}N_6O_6$ *$[M+H]^+$*	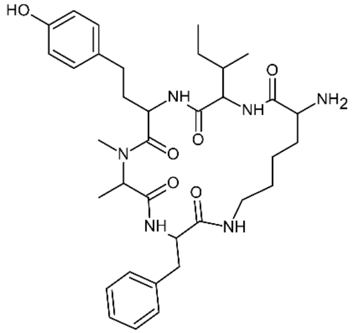	n.a.	5	21
Atenolol acid	Atenolol	$C_{14}H_{21}NO_4$ *$[M+H]^+$*	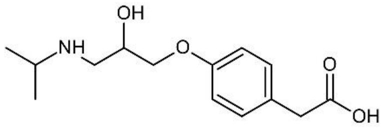	10.2	2a	17

Table S11 continued.

Name	Precursor(s)	Formula / *Ion detected**	Structure	RT (min)	Confidence level[23]	Reference
Cyanopeptolin-TP818	Cyanopeptolin A, B, C and/or D	$C_{40}H_{60}N_6O_{11}$ *$[M-H_2O+H]^+$* *$[M-H_2O-H]^-$* *$[M+H]^+$*	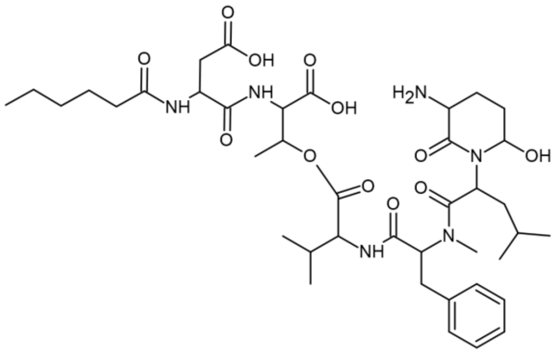	19.1	3	This study
Gabapentin-lactam	Gabapentin	$C_9H_{15}NO$ *$[M+H]^+$*	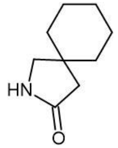	16.4	2a	19
MC-TP460	All identified MC	$C_{25}H_{36}N_2O_6$ *$[M+H]^+$* *$[M-H]^-$*	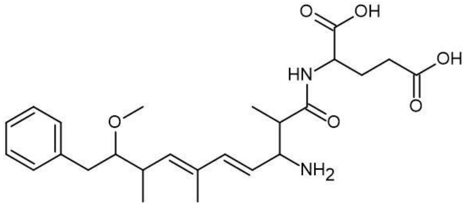	15.8	2b	30
MC-TP543	All identified MC	$C_{54}H_{77}N_5O_{13}$ *$[M+H]^+$* *$[M-H]^-$*	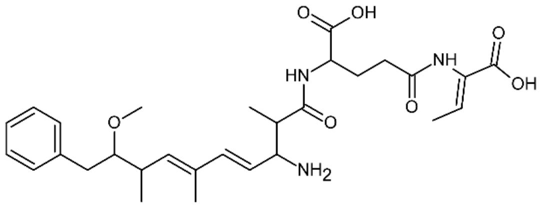	16.9	2b	22

Table S11 continued.

Name	Precursor(s)	Formula / *Ion detected**	Structure	RT (min)	Confidence level[23]	Reference
MC-TP614	All identified MC	$C_{32}H_{46}N_4O_8$<br>*[M+H]*$^+$<br>*[M-H]*$^-$	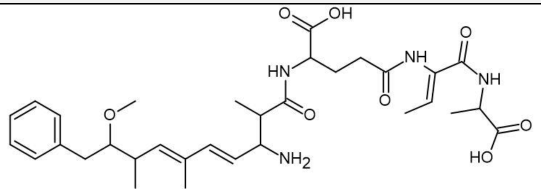	16.3	2b	[15]
MC-TP885	[D-Asp[3],(E)-Dhb[7]]MCRR	$C_{42}H_{63}N_9O_{12}$<br>*[M+2H]*$^{2+}$<br>*[M-H]*$^-$<br>*[M+H]*$^+$	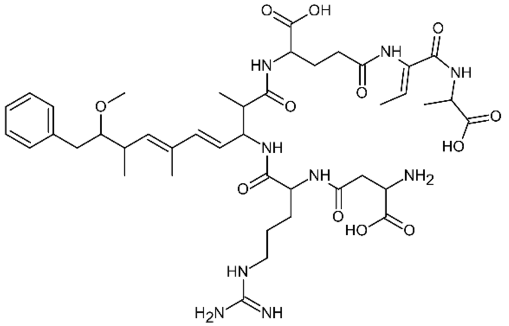	15.6	2b	This study
MC-TP980	[D-Asp[3],(E)-Dhb[7]]MCRR	$C_{47}H_{68}N_{10}O_{13}$<br>*[M+H]*$^+$<br>*[M-H]*$^-$<br>*[M+2H]*$^{2+}$	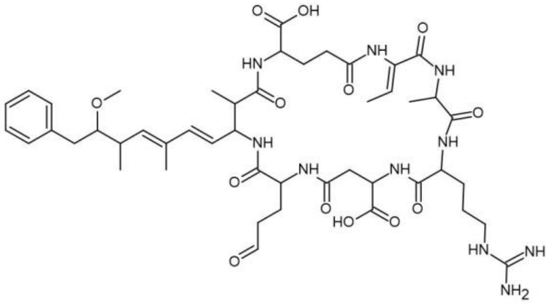	18.1	2b	[21]

Table S11 continued.

Name	Precursor(s)	Formula / *Ion detected**	Structure	RT (min)	Confidence level[23]	Reference
Planktocyclin-sulfoxide**	Planktocyclin	$C_{39}H_{60}N_8O_9S$ *[M+H]$^+$* *[M-H]$^-$*	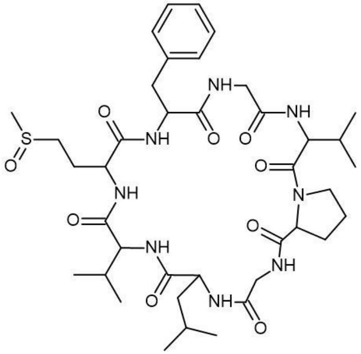	18.6 and 18.7****	2b	This study

* When multiple ion charges were detected, they are ranked by intensity

** Abiotic transformation products

*** Only measured in the DWTP. Oscillamide Y wasn't present in laboratory-scale experiments. The RT are not given as they are from a different elution method and are not comparable to the other bioTPs

**** Two stereoisomers corresponding to R and S sulfoxide enantiomers

Scheme S1. Scheme of the drinking water treatment train Lengg (Zürich, Switzerland). Arrows represent sampling points. Raw water DOC concentration was 1.5 mgC L^{-1}, pH 8.4 ± 0.2 and temperature 7°C. Pre-ozonation and intermediate ozonation dose was 0.5 and 0.3 mgO_3 L^{-1}, respectively. Sand vertical velocity was 1.2 m h^{-1}, resulting in a contact time of about 1.1 h.

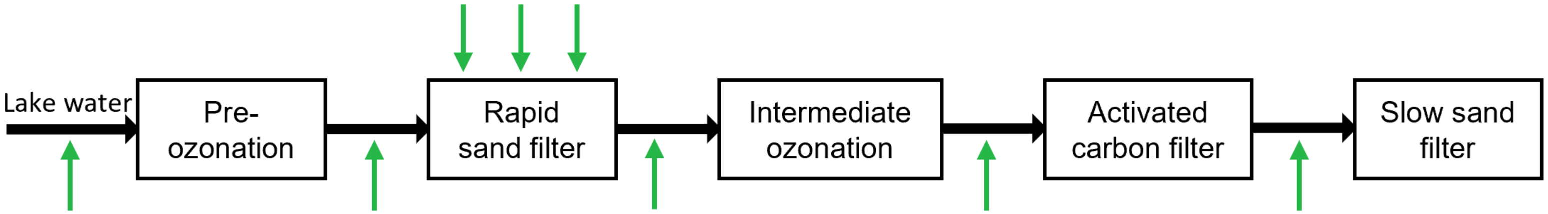

Scheme S2. Structures of identified cyano-metabolites.

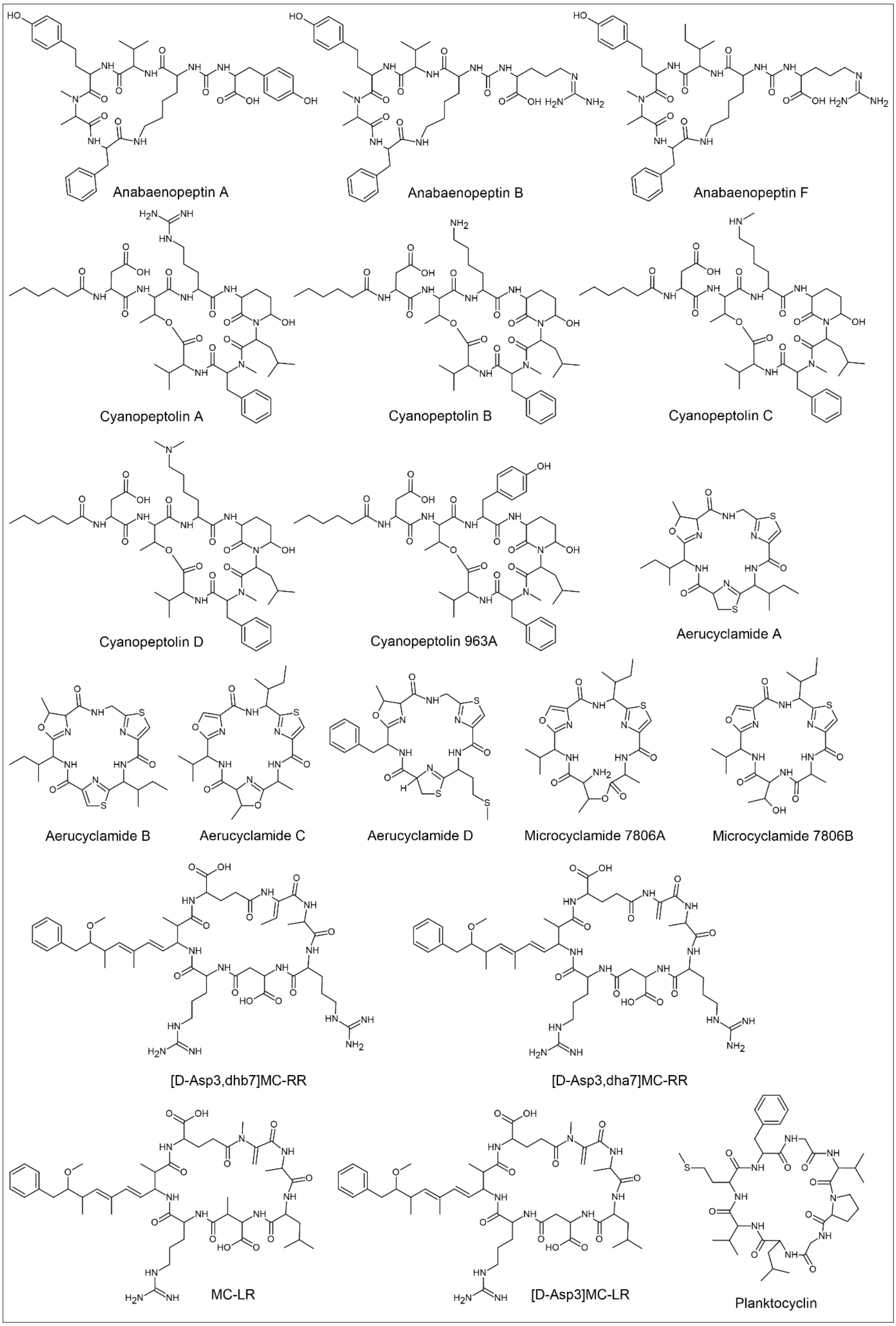

Scheme S3. Structures of the selected micropollutants.

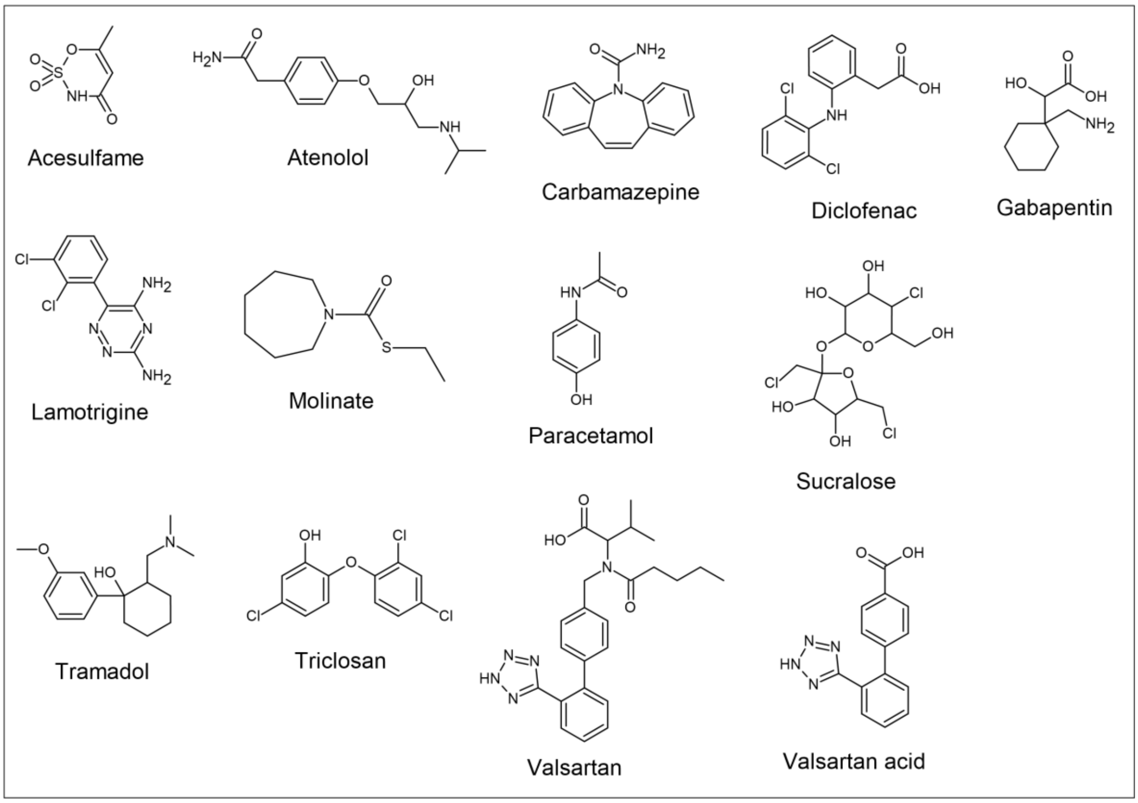

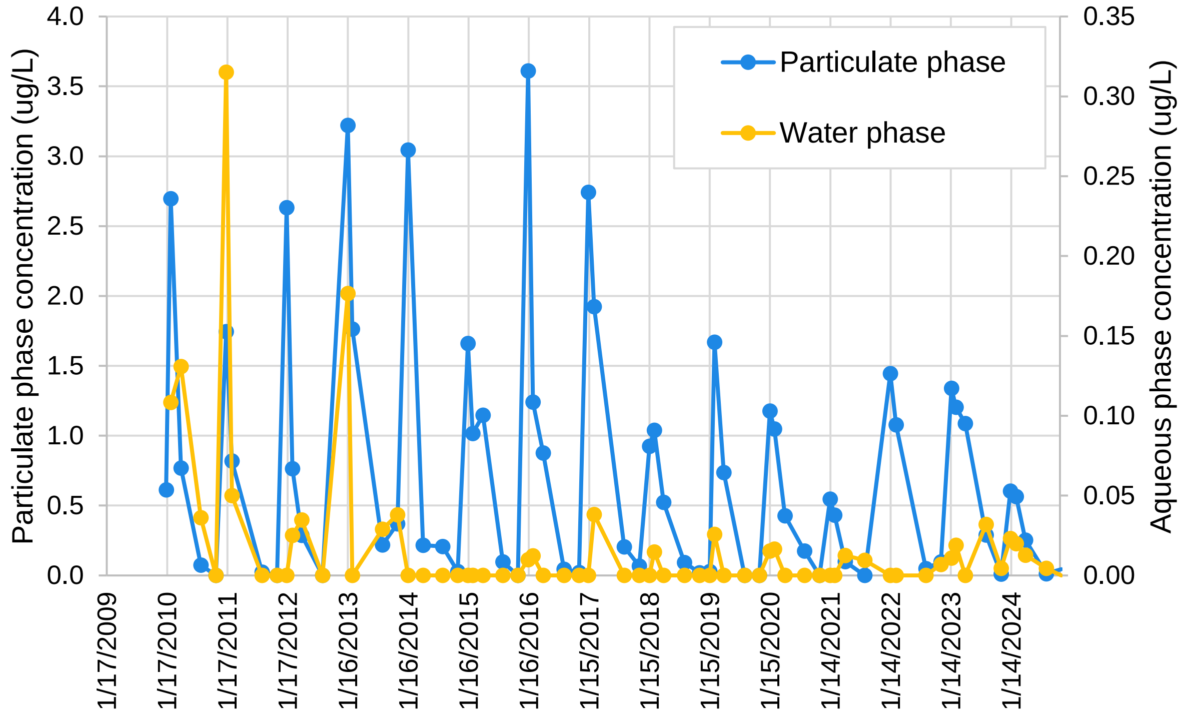

Figure S1. Concentration of [D-Asp^3,(E)-Dhb^7]MC-RR entering the drinking water treatment plant Lengg between 2010 and 2024. Data provided by Water Supply Zürich (Stadt Zürich Wasserversorgung).

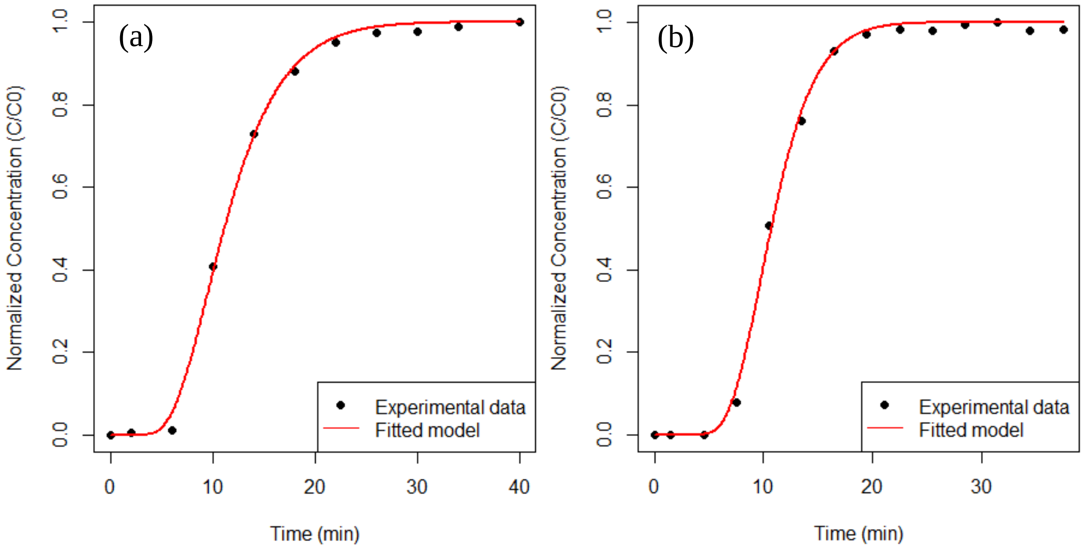

Figure S2. Fitting of the salt tracer breakthrough for (a) column #1 and (b) column #2 (see Text S2). Fitted porosity is 0.45 for column #1 and 0.44 for column #2. Fitted Peclet number is 13 for column #1 and 23 for column #2. Pore volumes, calculated from the porosity, are 11.2 mL for column #1 and 10.7 mL for column #2.

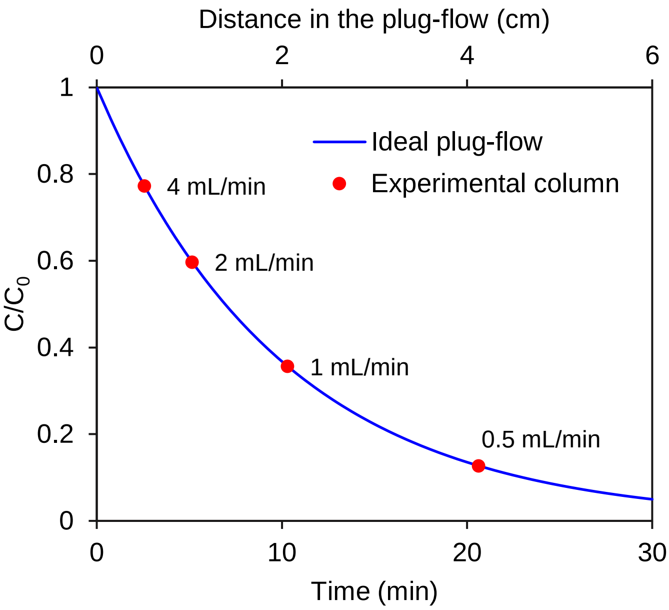

Figure S3. Analogy between an ideal plug-flow reactor and column experiments. In an ideal plug-flow reactor, the concentration decrease across the length of the reactor. In our column experiments, the concentration was not monitored across the column length, but only at the inlet and outlet. The flow rate was therefore varied to obtained various contact times.

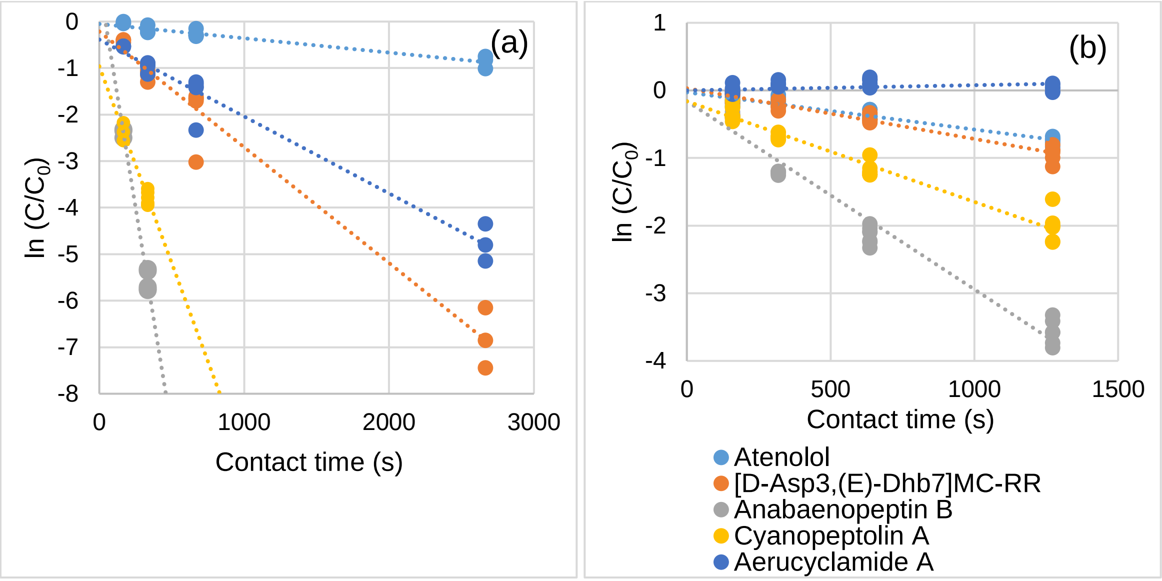

Figure S4. Examples of first-order plots for the elimination of target compounds used to calculate apparent first-order k_{app} in (a) column #1 and (b) column #2 (see text S4.1).

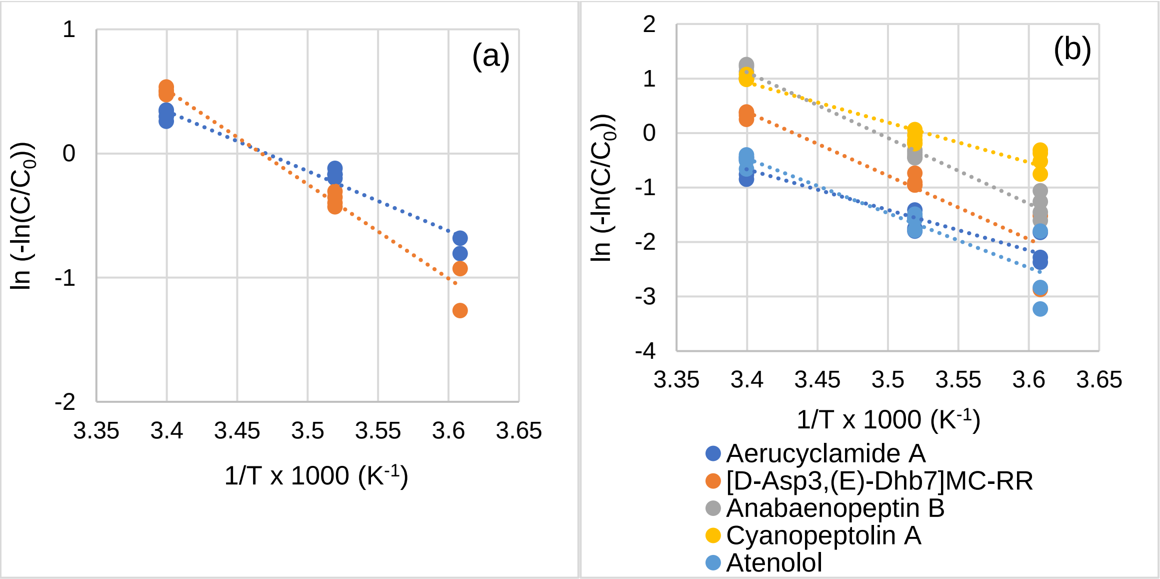

Figure S5. Examples of plots of the Arrhenius equation to calculate E_a in (a) column #1 and (b) column #2 (see text S4.1).

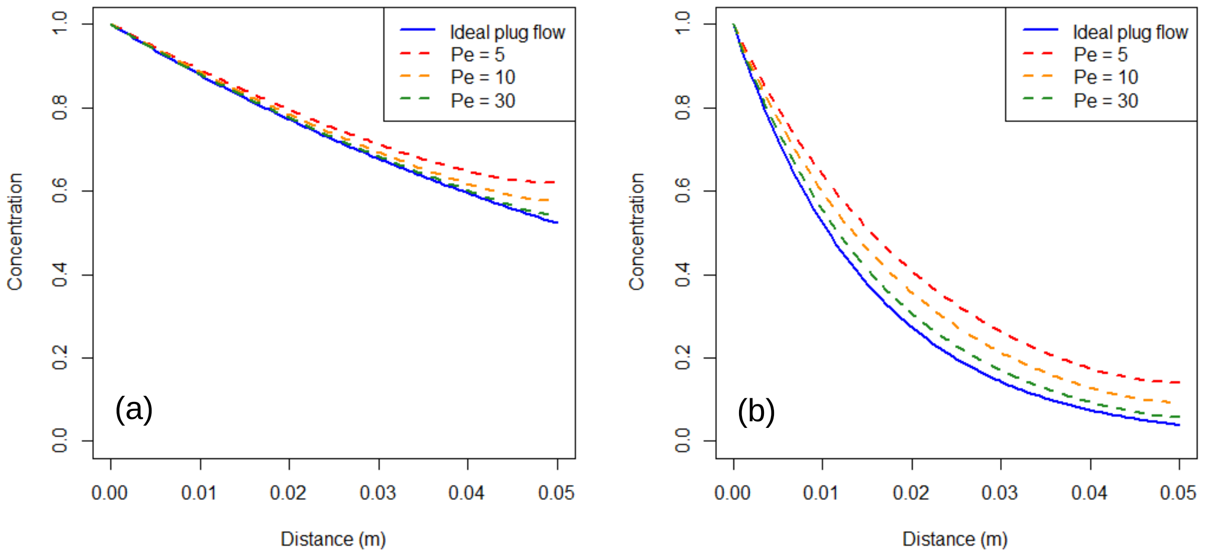

Figure S6. Simulation of first-order concentration decrease across the sand column for ideal and non-ideal plug-flow systems (variation of Peclet number (*Pe*), see Text S4.2). Input: (a) $k = 0.001\ s^{-1}$ and (b) $0.005\ s^{-1}$, porosity = 0.44, flow rate = 1 mL min^{-1}.

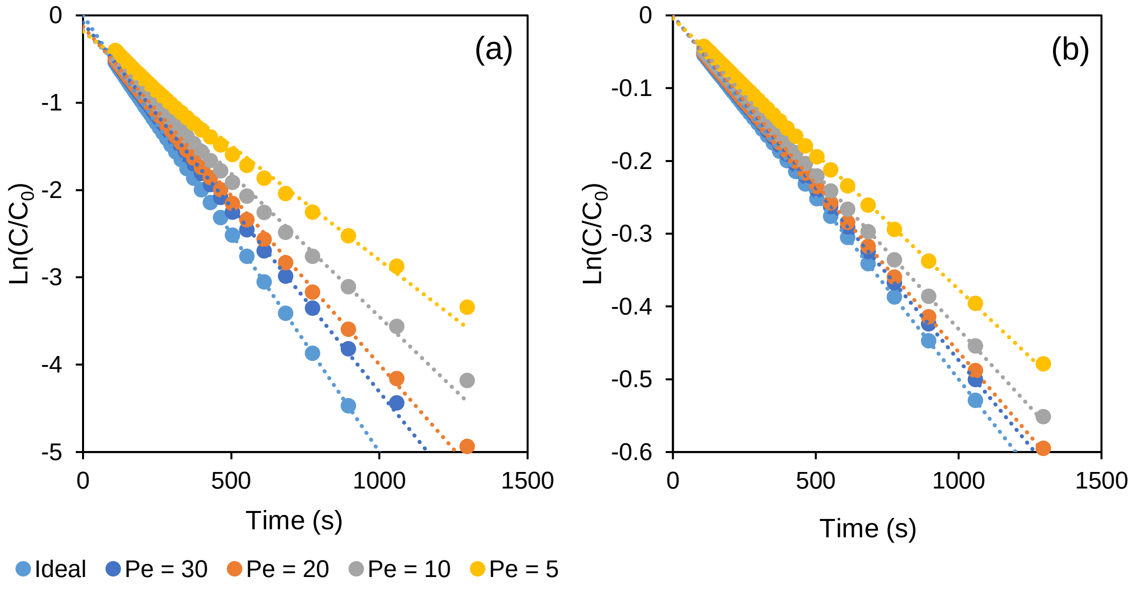

Figure S7. Examples of simulation of $\ln(C/C_0)$ vs time plots for ideal and non-ideal plug-flow systems (variation of Peclet number (*Pe*), see Text S4.2). Input: (a) $k = 0.005\ s^{-1}$ and (b) 0.0005 s^{-1}, porosity = 0.44, flow rate = 0.5-6 mL min^{-1}.

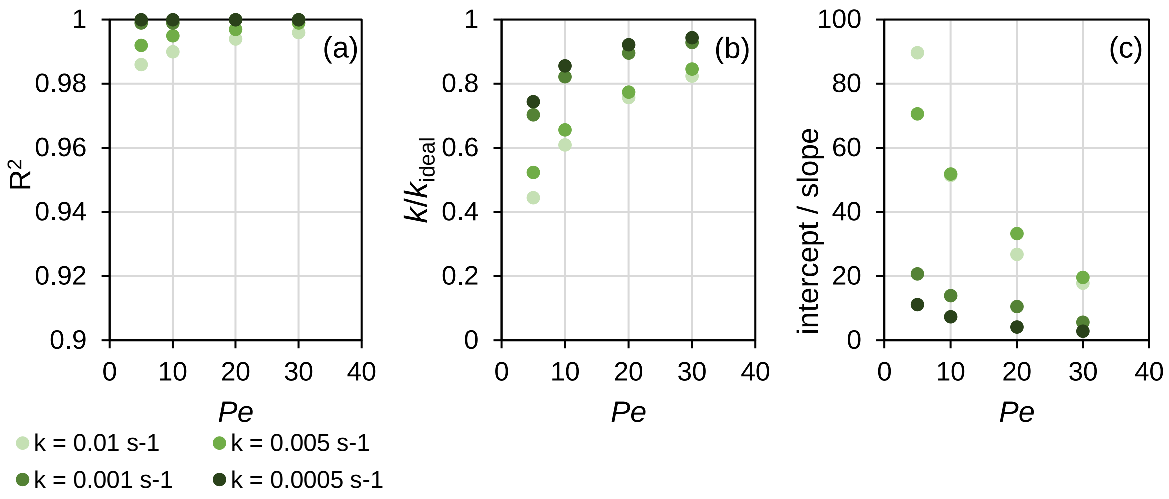

Figure S8. Simulated effect of non-ideal plug flow (variation of Peclet number (*Pe*), see Text S4.2) on first-order linear regressions. (a) Effect on R^2, (b) effect on the ratio between k for non-ideal plug flow and k for ideal plug flow (k_{ideal}), (c) effect on the ratio between ln(C/C_0) vs time y-intercept (ideally = 0) and slope (see Text S4.2). Input: k = 0.0005-0.01 s^{-1}, porosity = 0.44, flow rate = 0.5-6 mL min^{-1}.

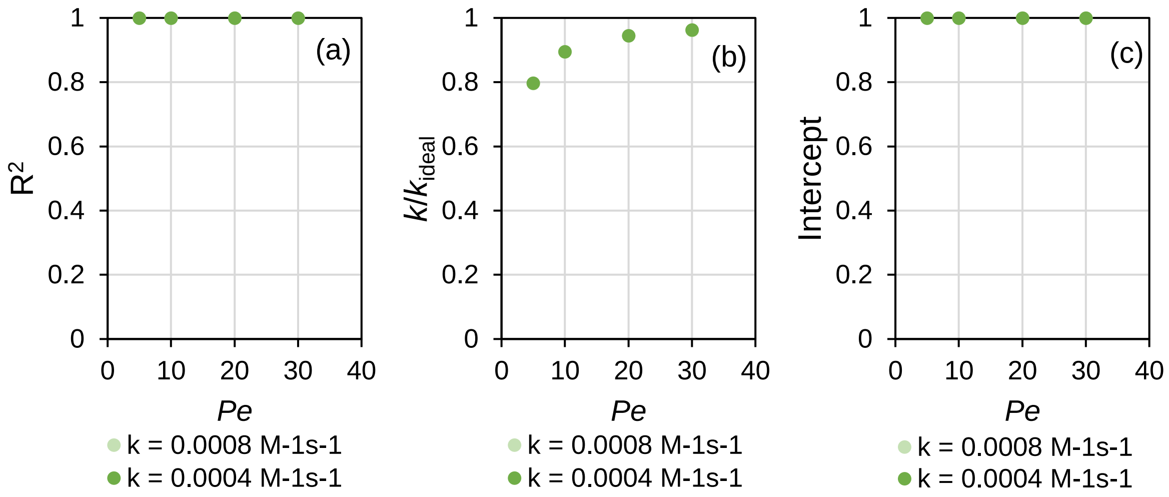

Figure S9. Simulated effect of non-ideal plug flow (variation of Péclet number (*Pe*), see Text S4.2) on zero-order linear regressions. (a) Effect on R^2, (b) effect on the ratio between k for non-ideal plug flow and k for ideal plug flow (k_{ideal}), (c) effect on the C/C_0 vs time y-intercept (ideally = 1) (see Text S4.2). Input: k = 0.0005-0.01 s^{-1}, porosity = 0.44, flow rate = 0.5-6 mL min^{-1}.

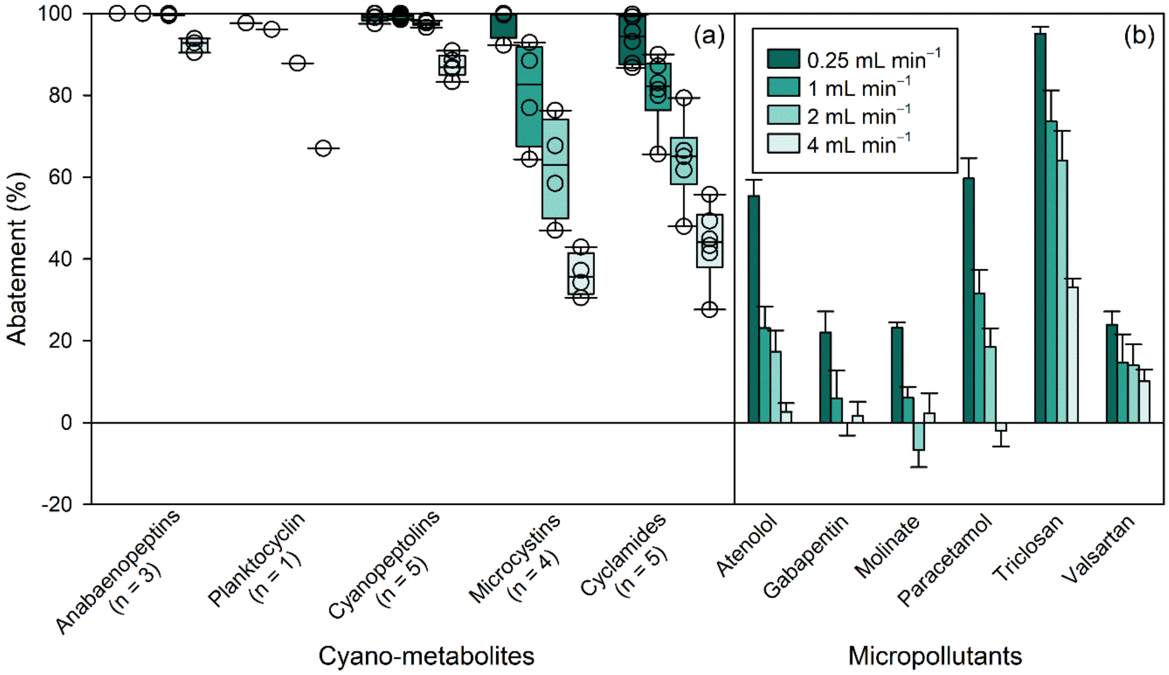

Figure S10. Effect of flow rate on (a) the relative abatement of cyano-metabolites and (b) micropollutants in laboratory sand column #1 (experiments labelled A, C, D and E, see Table S4). Cyano-metabolite abatement is shown as a box plot, with individual cyano-metabolite abatements shown as circles. The full data set is provided in Table S6. Experimental conditions: temperature = 21°C, [cyano-metabolites] = 19.2 $mg_{biomass\text{-}equivalent}$ L^{-1}, [micropollutants] = 0.6-6.3 µg L^{-1}.

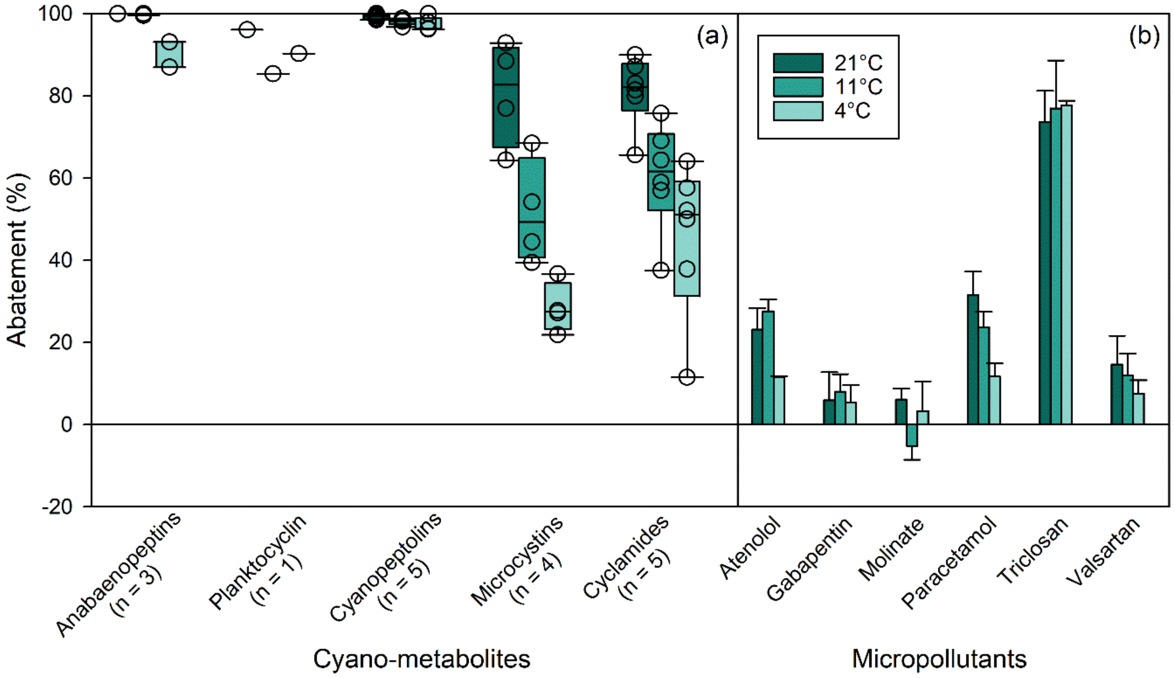

Figure S11. Effect of the temperature on (a) the relative abatement of cyano-metabolites and (b) micropollutants in laboratory sand column #1 (experiments labelled C, F and G, see Table S4). Cyano-metabolite abatement is shown as a box plot, with individual cyano-metabolite abatements shown as circles. The full data set is provided in Table S6. Experimental conditions: flow rate = 1mL min^{-1}, [cyano-metabolites] = 19.2 mg$_{\text{biomass-equivalent}}$ L^{-1}, [micropollutants] = 0.6-6.3 µg L^{-1}.

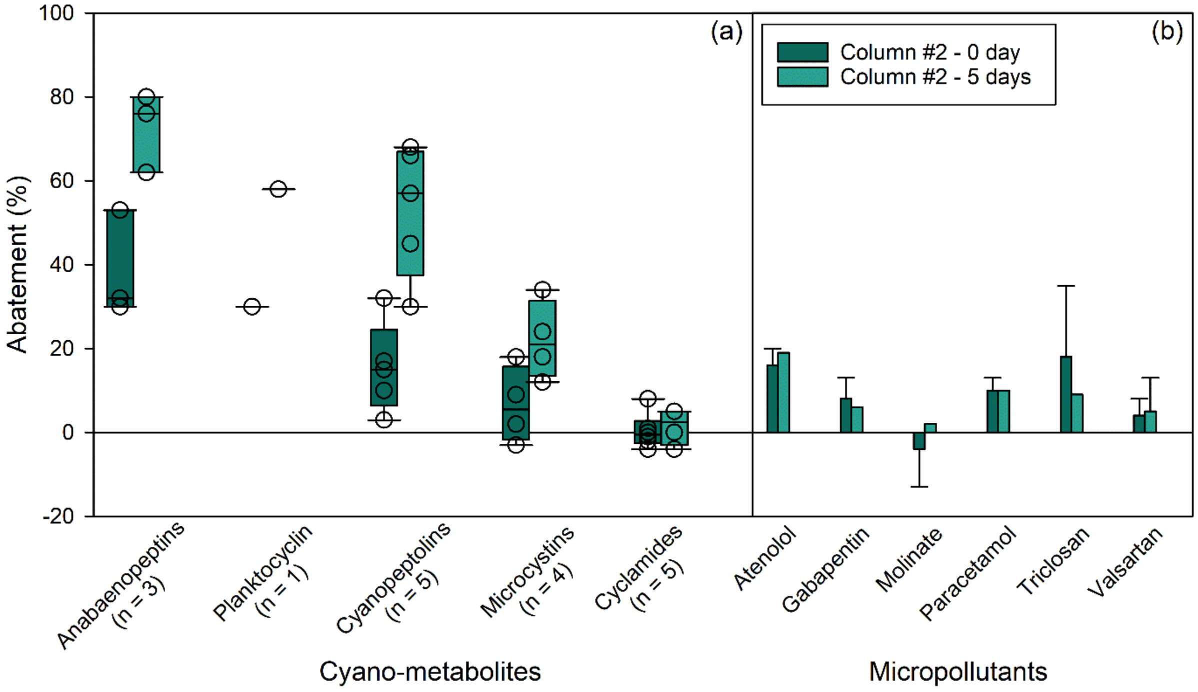

Figure S12. Effect of exposure time on (a) the abatement of cyano-metabolites and (b) micropollutants in column #2 (experiments labelled C in column #1 and C1 to C3 in column #2, see Table S4). The same experiment was run in column #1, after extended pre-exposure to cyano-metabolites and micropollutants, and in column #2 after 1, 4 and 25 days of exposure. Cyano-metabolite abatements are shown as a box plot, with individual cyano-metabolite abatement shown as circles and number of individual compounds (n) marked in each group. The full data set is provided in S6. Experimental conditions: flow rate = 4 mL min^{-1}, [cyano-metabolites] = 19.2 mg$_{\text{biomass-equivalent}}$ L^{-1}, [micropollutants] = 0.6-6.3 µg L^{-1}, T = 21°C.

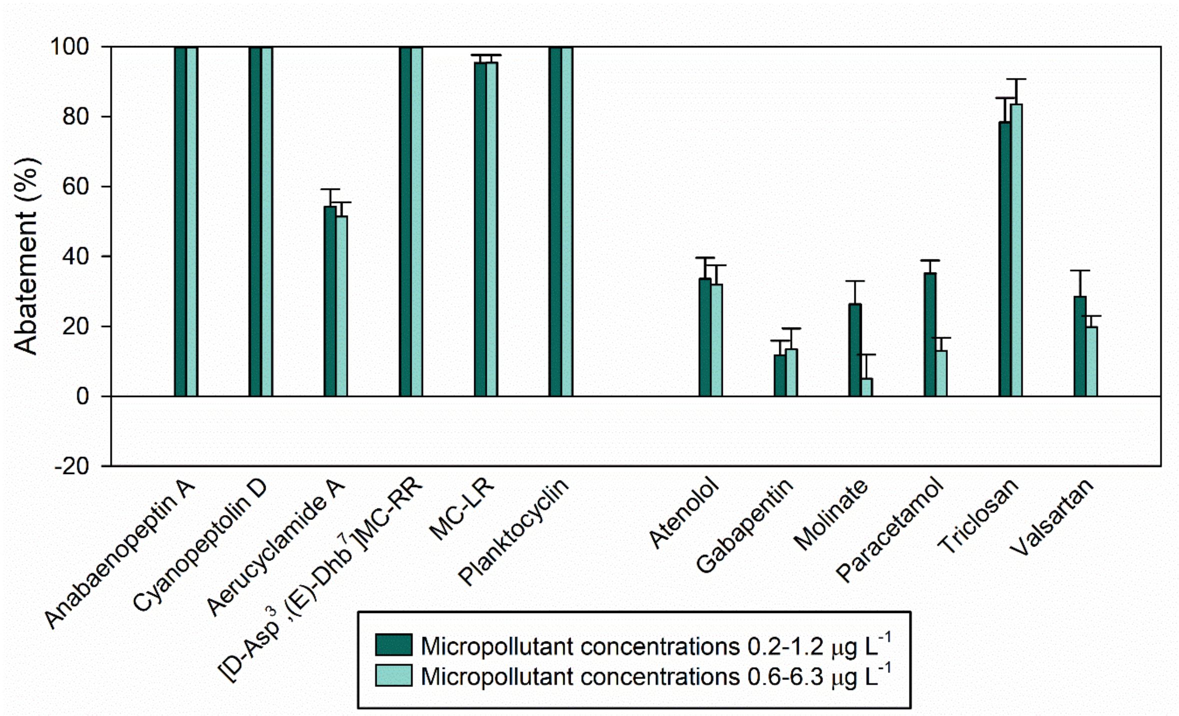

Figure S13. Relative abatement of selected cyano-metabolites and micropollutants at fixed cyano-metabolite concentration and varying micropollutant concentrations (experiments labelled J2 and L, see Table S4). The full data set is provided in Table S6. Experimental conditions: flow rate = 1 mL min^{-1}, [cyano-metabolites] = 3.8 mg$_{\text{biomass-equivalent}}$ L^{-1}, [micropollutants] = 0.2-1.2 or 0.6-6.3 µg L^{-1}, 21°C.

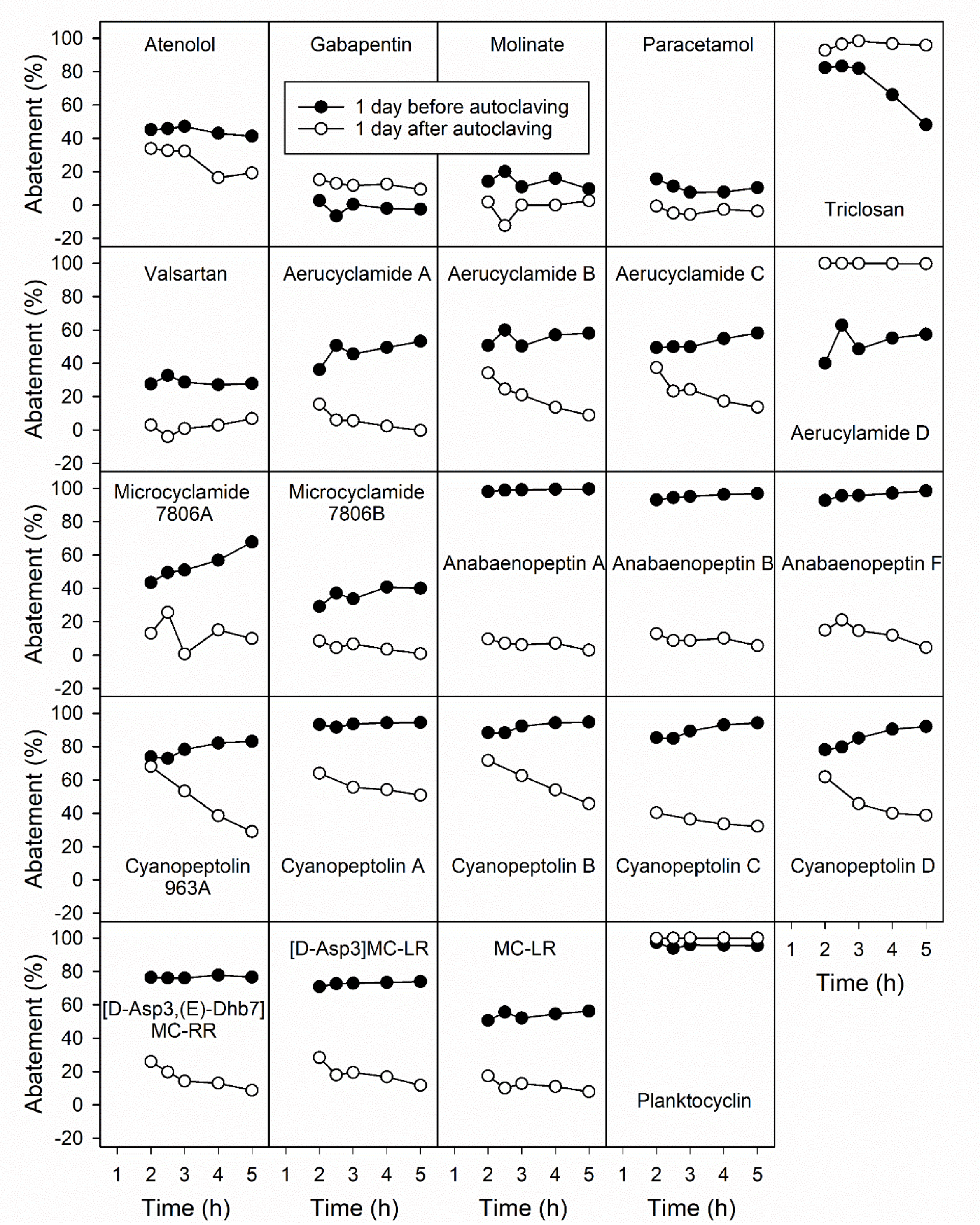

Figure S14. Relative abatement of all compounds with observable abatement as a function of time in column #2. The abatement is shown 1 day before autoclaving the column (closed circles), and 1 day after autoclaving (open circles) (experiments labelled C3 and M, see Table S4). Experimental conditions: flow rate = 1 mL min^{-1}, [cyano-metabolites] = 19.2 mg$_{\text{biomass-equivalent}}$ L^{-1}, [micropollutants] = 0.6-6.3 µg L^{-1}, T = 21°C.

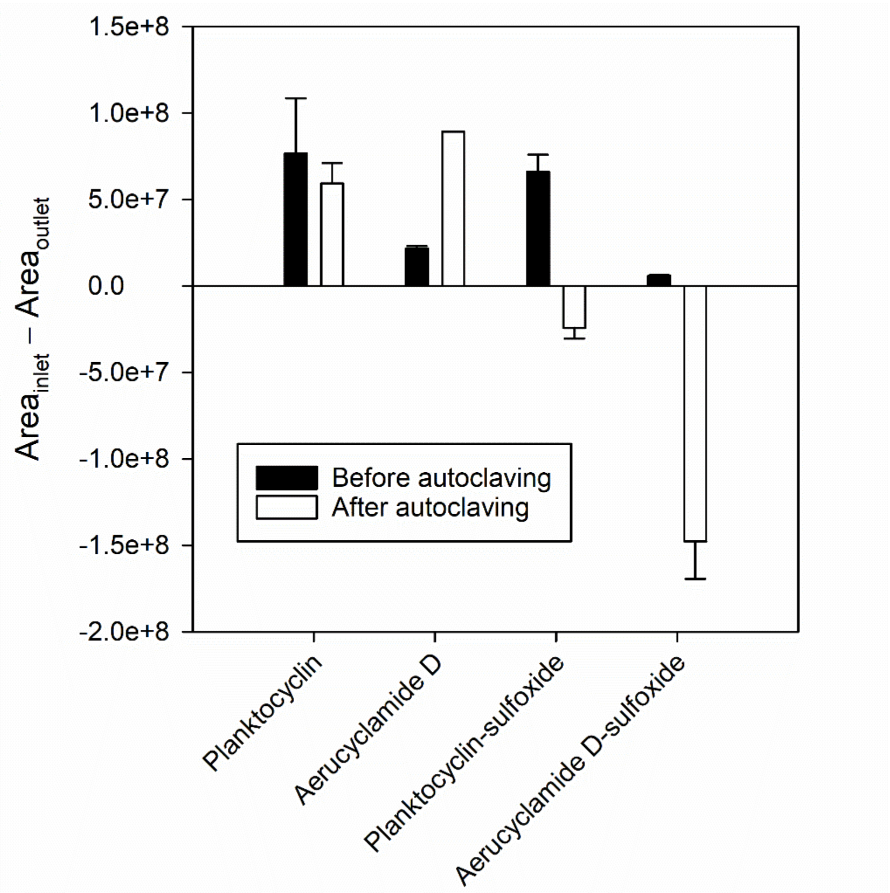

Figure S15. Peak area difference between the column inlet and outlet for planktocyclin, aerucyclamide D and their sulfoxide products. The peak areas are shown before (black bars) and after (white bars) autoclaving (experiments labelled C3 and M in column #2, see Table S4). Experimental conditions: flow rate = 1 mL min^{-1}, [cyano-metabolites] = 19.2 mg$_{\text{biomass-equivalent}}$ L^{-1}, [micropollutants] = 0.6-6.3 µg L^{-1}., T = 21°C.

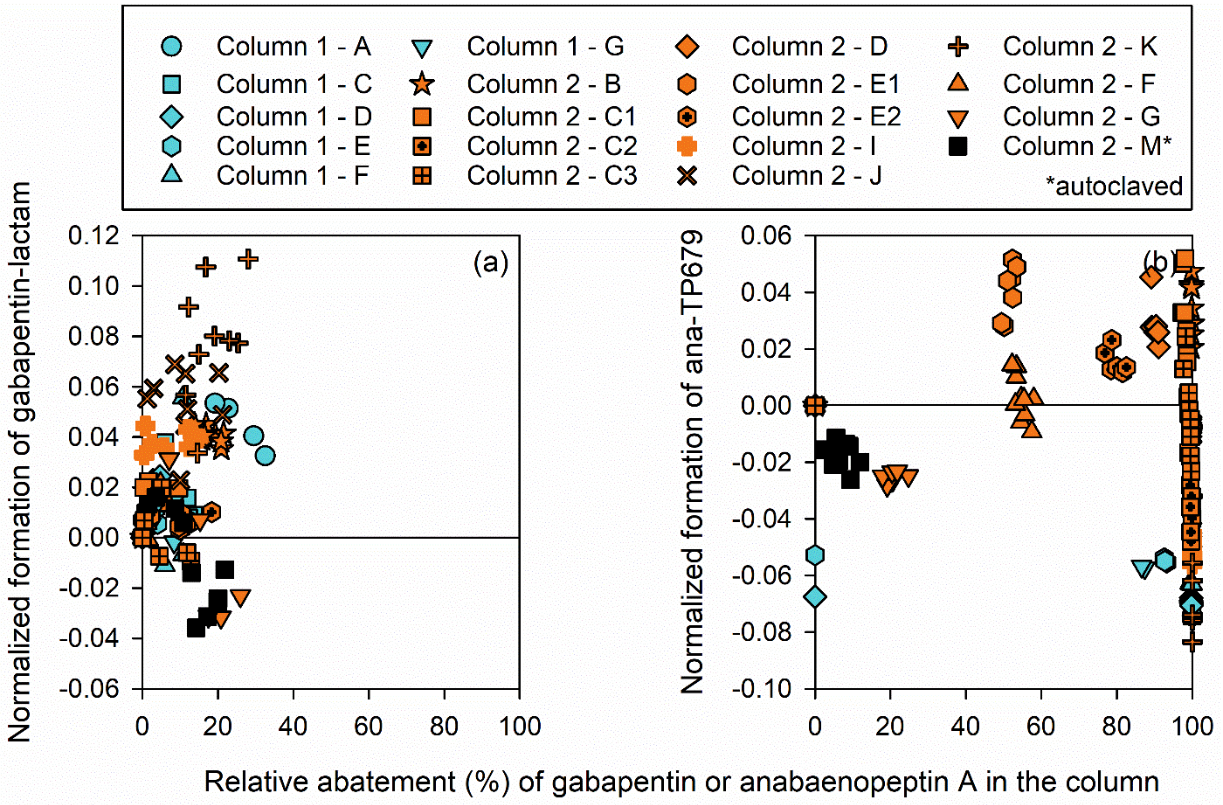

Figure S16. Formation of biological transformation products during the column experiments. Formation of (a) gabapentin-lactam and (b) ana-TP679 as a function of the relative abatement of (a) gabapentin and (b) anabaenopeptin A. The areas of the product are normalized to the area of the precursor before the column. All experiments labelled from A-M are shown as individual data sets (see Table S4 for the details corresponding to each experiment label). Both gabapentin-lactam and ana-TP679 were present in the inlet and were subtracted from the outlet to obtain the formation (or decrease if negative).

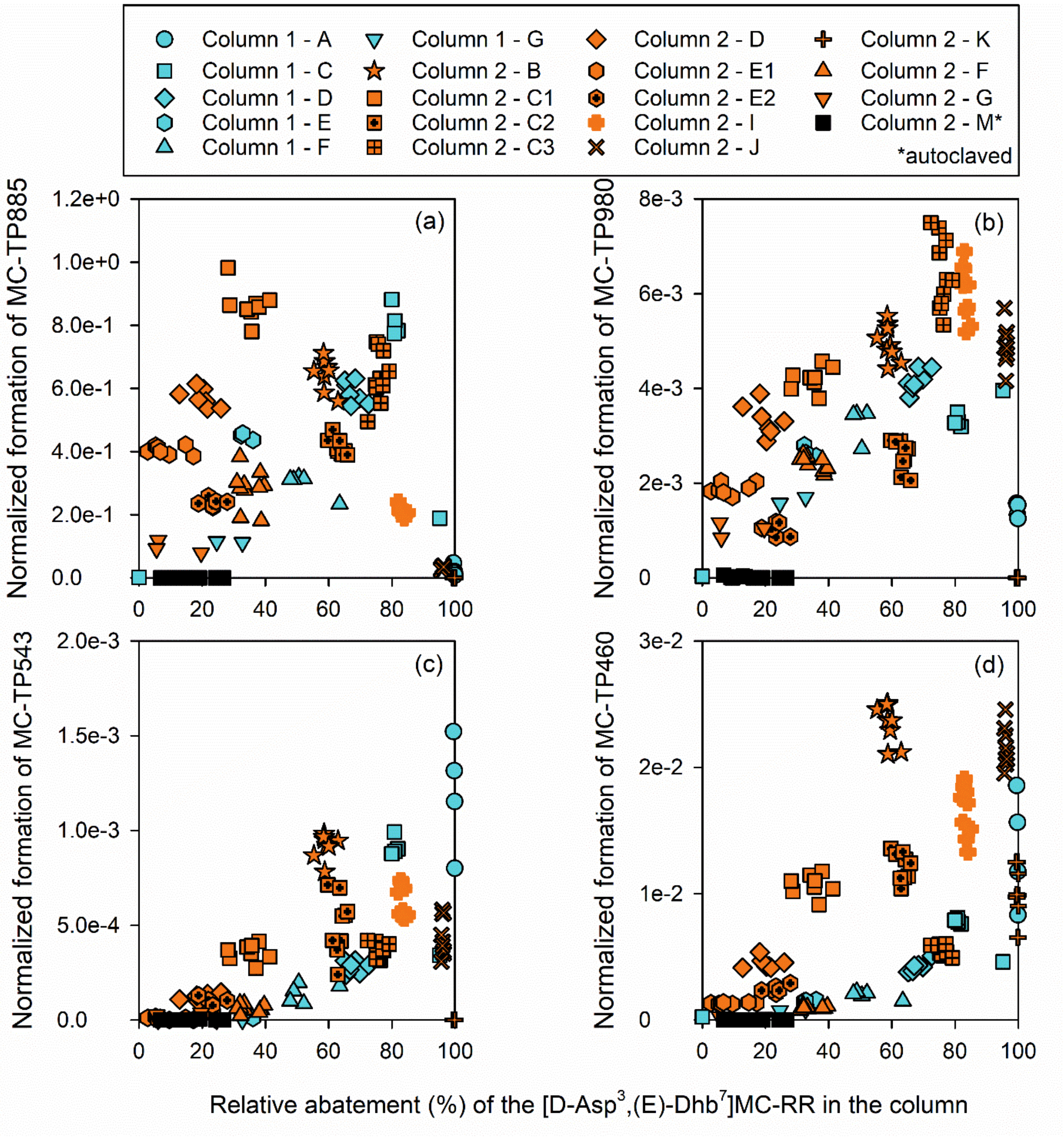

Figure S17. Formation of biological transformation products during the column experiments. Formation of (a) MC-TP885, (b) MC-TP980, (c) MC-TP543 and (d) MC-TP460 as a function of the relative abatement of [D-Asp^{3},(E)-Dhb^{7}]MC-RR. The areas of the product are normalized to the area of the precursor before the column. All experiments labelled from A-M are shown as individual data sets (see Table S4 for the details corresponding to each experiment label).

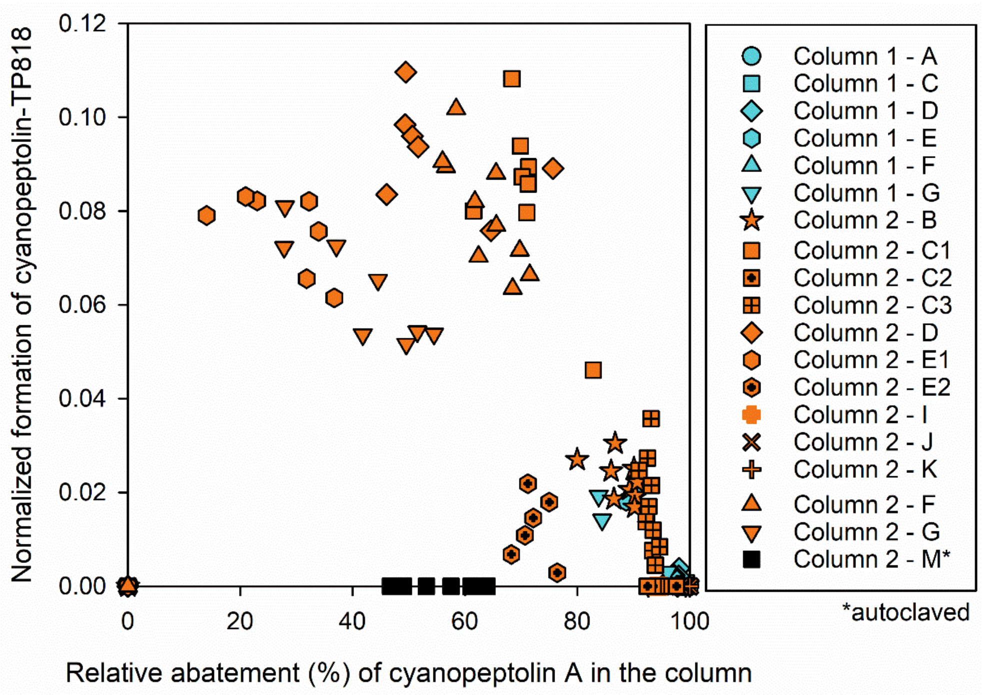

Figure S18. Formation of cyanopeptolin-TP818 as a function of the relative abatement of cyanopeptolin A. The areas of the product are normalized to the area of the precursor before the column. All experiments labelled from A-M are shown as individual data sets (see Table S4 for the details corresponding to each experiment label).

References

(1) Natumi, R.; Marcotullio, S.; Janssen, E. M. L. Phototransformation kinetics of cyanobacterial toxins and secondary metabolites in surface waters. *Environ. Sci. Eur.* **2021**, *33* (1), 26. DOI: 10.1186/s12302-021-00465-3.

(2) Zearley, T. L.; Summers, R. S. Removal of Trace Organic Micropollutants by Drinking Water Biological Filters. *Environ. Sci. Technol.* **2012**, *46* (17), 9412-9419. DOI: 10.1021/es301428e.

(3) Gulde, R.; Clerc, B.; Rutsch, M.; Helbing, J.; Salhi, E.; McArdell, C. S.; von Gunten, U. Oxidation of 51 micropollutants during drinking water ozonation: Formation of transformation products and their fate during biological post-filtration. *Water Res.* **2021**, *207*, 117812. DOI: https://doi.org/10.1016/j.watres.2021.117812.

(4) Margot, J.; Rossi, L.; Barry, D. A.; Holliger, C. A review of the fate of micropollutants in wastewater treatment plants. *WIREs Water* **2015**, *2* (5), 457-487. DOI: https://doi.org/10.1002/wat2.1090.

(5) Sanchez-Huerta, C.; Medina, J. S.; Wang, C.; Fortunato, L.; Hong, P.-Y. Understanding the role of sorption and biodegradation in the removal of organic micropollutants by membrane aerated biofilm reactor (MABR) with different biofilm thickness. *Water Res.* **2023**, *236*, 119935. DOI: https://doi.org/10.1016/j.watres.2023.119935.

(6) Crittenden, J. C.; Trussell, R. R.; Hand, D. W.; Howe, K. J.; Tchobanoglous, G. *MWH's Water Treatment: Principles and Design*; John Wiley & Sons, 2012. DOI: 10.1002/9781118131473.

(7) van Genuchten, M. T.; Alves, W. J. *Analytical solutions of the one-dimensional convective-dispersive solute transport equation*; 1982. https://www.ars.usda.gov/ARSUserFiles/20360500/pdf_pubs/P075.pdf.

(8) Bear, J. *Dynamics of Fluids in Porous Media*; Dover Publications, 1972.

(9) Bisswanger, H. *Enzyme Kinetics: Principles and methods (2nd, revised and updated ed.)*; Wiley-VCH, 2008.

(10) Arcus, V. L.; Mulholland, A. J. Temperature, Dynamics, and Enzyme-Catalyzed Reaction Rates. *Annual Review of Biophysics* **2020**, *49* (Volume 49, 2020), 163-180. DOI: https://doi.org/10.1146/annurev-biophys-121219-081520.

(11) Rittmann, B. E.; McCarty, P. L. *Environmental Biotechnology: Principles and Applications*; McGraw-Hill Education, 2001.

(12) Sneath, P. H. A.; Sokal, R. R. S. *Numerical Taxonomy (by) Peter H.A. Sneath (and) Robert R. Sokal: The Principles and Practice of Numerical Classification*; W.H. Freeman, 1973.

(13) Suzuki, R.; Shimodaira, H. Pvclust: an R package for assessing the uncertainty in hierarchical clustering. *Bioinformatics* **2006**, *22* (12), 1540-1542. DOI: 10.1093/bioinformatics/btl117.

(14) Steinweg, J. M.; Jagadamma, S.; Frerichs, J.; Mayes, M. A. Activation Energy of Extracellular Enzymes in Soils from Different Biomes. *PLOS ONE* **2013**, *8* (3), e59943. DOI: 10.1371/journal.pone.0059943.

(15) Bourne, D. G.; Jones, G. J.; Blakeley, R. L.; Jones, A.; Negri, A. P.; Riddles, P. Enzymatic pathway for the bacterial degradation of the cyanobacterial cyclic peptide toxin microcystin LR. *Appl. Environ. Microbiol.* **1996**, *62* (11), 4086-4094. DOI: 10.1128/aem.62.11.4086-4094.1996.

(16) Zhang, L.; Hu, J.; Zhu, R.; Zhou, Q.; Chen, J. Degradation of paracetamol by pure bacterial cultures and their microbial consortium. *Appl. Microbiol. Biotechnol.* **2013**, *97* (8), 3687-3698. DOI: 10.1007/s00253-012-4170-5.

(17) Helbling, D. E.; Hollender, J.; Kohler, H.-P. E.; Singer, H.; Fenner, K. High-Throughput Identification of Microbial Transformation Products of Organic Micropollutants. *Environ. Sci. Technol.* **2010**, *44* (17), 6621-6627. DOI: https://doi.org/10.1021/es100970m.

(18) Bester, K. Triclosan in a sewage treatment process—balances and monitoring data. *Water Res.* **2003**, *37* (16), 3891-3896. DOI: https://doi.org/10.1016/S0043-1354(03)00335-X.
(19) Henning, N.; Kunkel, U.; Wick, A.; Ternes, T. A. Biotransformation of gabapentin in surface water matrices under different redox conditions and the occurrence of one major TP in the aquatic environment. *Water Res.* **2018**, *137*, 290-300. DOI: https://doi.org/10.1016/j.watres.2018.01.027.
(20) Nunes, O. C.; Lopes, A. R.; Manaia, C. M. Microbial degradation of the herbicide molinate by defined cultures and in the environment. *Appl. Microbiol. Biotechnol.* **2013**, *97* (24), 10275-10291. DOI: 10.1007/s00253-013-5316-9.
(21) Wang, X.; Ingold, A.; Janssen, E. M. L. Biotransformation Dynamics and Products of Cyanobacterial Secondary Metabolites in Surface Waters. *Environ. Sci. Technol.* **2025**, *59* (38), 20726–20737. DOI: 10.1021/acs.est.5c09247.
(22) Salter, C.; Westrick, J. A.; Chaganti, S. R.; Birbeck, J. A.; Peraino, N. J.; Weisener, C. G. Elucidating microbial mechanisms of microcystin-LR degradation in Lake Erie beach sand through metabolomics and metatranscriptomics. *Water Res.* **2023**, *247*, 120816. DOI: https://doi.org/10.1016/j.watres.2023.120816.
(23) Schymanski, E. L.; Jeon, J.; Gulde, R.; Fenner, K.; Ruff, M.; Singer, H. P.; Hollender, J. Identifying Small Molecules via High Resolution Mass Spectrometry: Communicating Confidence. *Environ. Sci. Technol.* **2014**, *48* (4), 2097-2098. DOI: 10.1021/es5002105.
(24) Xiong, L.; Teng, J. L. L.; Botelho, M. G.; Lo, R. C.; Lau, S. K. P.; Woo, P. C. Y. Arginine Metabolism in Bacterial Pathogenesis and Cancer Therapy. *International Journal of Molecular Sciences* **2016**, *17* (3), 363.
(25) Rougé, V.; von Gunten, U.; Janssen, E. M. L. Reactivity of Cyanobacteria Metabolites with Ozone: Multicompound Competition Kinetics. *Environ. Sci. Technol.* **2024**, *58* (26), 11802-11811. DOI: https://doi.org/10.1021/acs.est.4c02242.
(26) Thompson, J. M. *Mass Spectrometry (1st ed.)*; Jenny Stanford Publishing, 2017. DOI: https://doi.org/10.1201/9781351207157.
(27) Steiner, T.; Schanbacher, F.; Lorenzen, W.; Enke, H.; Janssen, E. M. L.; Niedermeyer, T. H. J.; Gademann, K. UV–vis absorbance spectra, molar extinction coefficients and circular dichroism spectra for the two cyanobacterial metabolites anabaenopeptin A and anabaenopeptin B. *Data in Brief* **2024**, *57*, 110914. DOI: https://doi.org/10.1016/j.dib.2024.110914.
(28) S75 | CyanoMetDB | Comprehensive database of secondary metabolites from cyanobacteria (NORMAN-SLE-S75.0.3.0) [Data set]. Zenodo. (accessed 2025/04/02).
(29) Winer, B. J.; Brown, D. R.; Michels, K. M. *Statistical Principles in Experimental Design*; McGraw-Hill, 1991.
(30) Ding, Q.; Song, X.; Yuan, M.; Xu, K.; Huang, J.; Sun, R.; Zhang, J.; Yin, L.; Pu, Y. Microcystin-LR exposure enhances toxin-degrading capacity and reduces metabolic diversity of sediment microbial communities. *Environ. Poll.* **2022**, *311*, 119947. DOI: https://doi.org/10.1016/j.envpol.2022.119947.
